# Discovery of pyrazole-based analogs as CDK2 inhibitors with apoptotic-inducing activity: design, synthesis and molecular dynamics study†

**DOI:** 10.1039/d4ra06500j

**Published:** 2024-10-29

**Authors:** Ghada M. E. Ali, Menna A. Ewida, Amira M. Elmetwali, Heba A. Ewida, Riham F. George, Walaa R. Mahmoud, Nasser S. M. Ismail, Mahmoud S. Ahmed, Hanan H. Georgey

**Affiliations:** a Central Administration of Drug Control, EDA P.O. Box: 29 Cairo Egypt; b Department of Pharmaceutical Chemistry, Faculty of Pharmacy, Future University in Egypt Cairo 11835 Egypt Nasser.saad@fue.edu.eg; c Department of Pharmacology and Biochemistry, Faculty of Pharmacy, Future University in Egypt Cairo 11835 Egypt; d Pharmaceutical Sciences Department, School of Pharmacy, Texas Tech University Health Science Center Amarillo Texas USA mahmoudsalama.ahmed@ttuhsc.edu; e Pharmaceutical Chemistry Department, Faculty of Pharmacy, Cairo University Cairo 11562 Egypt; f Pharmaceutical Chemistry Department, Faculty of Pharmacy, Ain-Shams University Cairo 11566 Egypt; g Pharmaceutical Chemistry Department, Faculty of Pharmacy and Drug Technology, Egyptian Chinese University 11786 Cairo Egypt

## Abstract

The discovery of novel CDK2 inhibitors is crucial for developing targeted anticancer therapies. Thus, in this study, we aimed to design, synthesize, and evaluate a series of novel pyrazole derivatives (2a–g, 7a–d, 8a and b, 9, and 10) for their potential as CDK2/cyclin A2 enzyme inhibitors. The newly synthesized compounds were screened *in vitro* at 50 μM for CDK2 inhibition, followed by IC_50_ profiling of the most promising candidates. Compounds 4, 7a, 7d, and 9 exhibited the strongest inhibition, with IC_50_ values of 3.82, 2.0, 1.47, and 0.96 μM, respectively. To assess their anti-proliferative effects, all target compounds were further screened against a panel of 60 National Cancer Institute (NCI) cell lines representing various carcinoma types. Among them, compound 4 demonstrated exceptional anti-proliferative activity with a mean growth inhibition (GI) of 96.47% across the panel, while compound 9 showed a mean GI of 65.90%. Additionally, compounds 2b and 7c exhibited notable inhibition against MCF7 breast cancer cells, with GI rates of 86.1% and 79.41%, respectively. Compound 4 was selected for further five-dose concentration evaluations, displaying a full-panel GI_50_ value of 3.81 μM, with a subpanel range of 2.36–9.17 μM. Western blot analysis of compounds 4 and 9 in HCT-116 cell lines confirmed their inhibitory effects on CDK2. Furthermore, compound 4 induced significant cell cycle arrest at the G1 phase and promoted apoptosis. *In silico* molecular docking studies revealed that compounds 4, 7a, 7d, and 9 adopt a similar binding mode as AT7519 (I) within the CDK2 binding site. Molecular dynamics simulations further validated the stability of these compounds within the catalytic domain of CDK2. ADME/TOPKAT analyses indicated their favorable pharmacokinetic profiles, which were confirmed by their low toxicity in normal cell lines. Based on these findings, it was concluded that the synthesized pyrazole derivatives, particularly compound 4, show potent CDK2 inhibition and significant anticancer activity, with promising drug-like properties and minimal toxicity. This positions them as strong candidates for further development as CDK2-targeting anticancer agents.

## Abstract 2

The discovery of novel CDK2 inhibitors is crucial for developing targeted anticancer therapies.

## Graphical abstract

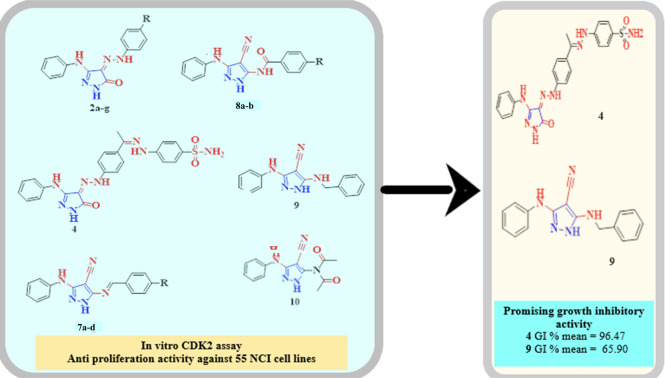

## 1. Introduction

Cell proliferation is regulated by an integrated network of proteins, which dictate the cell cycle events from order and timing perspectives. Cell cycle phases are controlled by a family of related serine/threonine proteins called cyclin-dependent kinases (CDKs), which become active upon association with their respective cyclin regulatory partners.^1^ Cell cycle progression is controlled by the formation of a CDK/cyclin complex, which is responsible for the phosphorylation of target genes, such as the tumor suppressor protein retinoblastoma (Rb). A CDK/cyclin complex is activated by mitogenic signals and inhibited by the activation of cell-cycle checkpoints in response to DNA damage.^2^ Cyclin-dependent kinase inhibitors (CKIs) negatively regulate CDKs/cyclin as the inhibitor of CDK4 (INK4) proteins (p16INK4a, p15INK4b, p18INK4c, and p19INK4d) and CDK2 (p21 and p27).^3^

CDK2-cyclin E is mainly responsible for the complete phosphorylation of retinoblastoma (Rb) in late G1, which allows the initiation of the S phase of the cell cycle, while CDK2-cyclin A eases S/G2 transition. Moreover, CDK2 plays an additional role in apoptosis, cell differentiation, immune response and the repair of normal DNA.^4–7^ Overexpression of CDK2 is predominant in many cancer types such as melanoma, glioblastoma, lymphoid tumor tissues and metastasis of prostate cancer. The irregular expression of CDK2 is often accompanied with the augmentation of its partner cyclins A and E in many human cancers as breast, endometrial, lung and thyroid carcinomas, melanoma, and osteosarcoma.^8,9^

In recent years, computational methods have become invaluable tools in cancer diagnosis and treatment, enabling the identification of potential therapeutic targets and facilitating the design of inhibitors through molecular modeling and dynamic simulation studies. These techniques provide significant insights into the binding interactions and stability of drug candidates, accelerating the drug discovery process.^10–13^

**Fig. 1:**
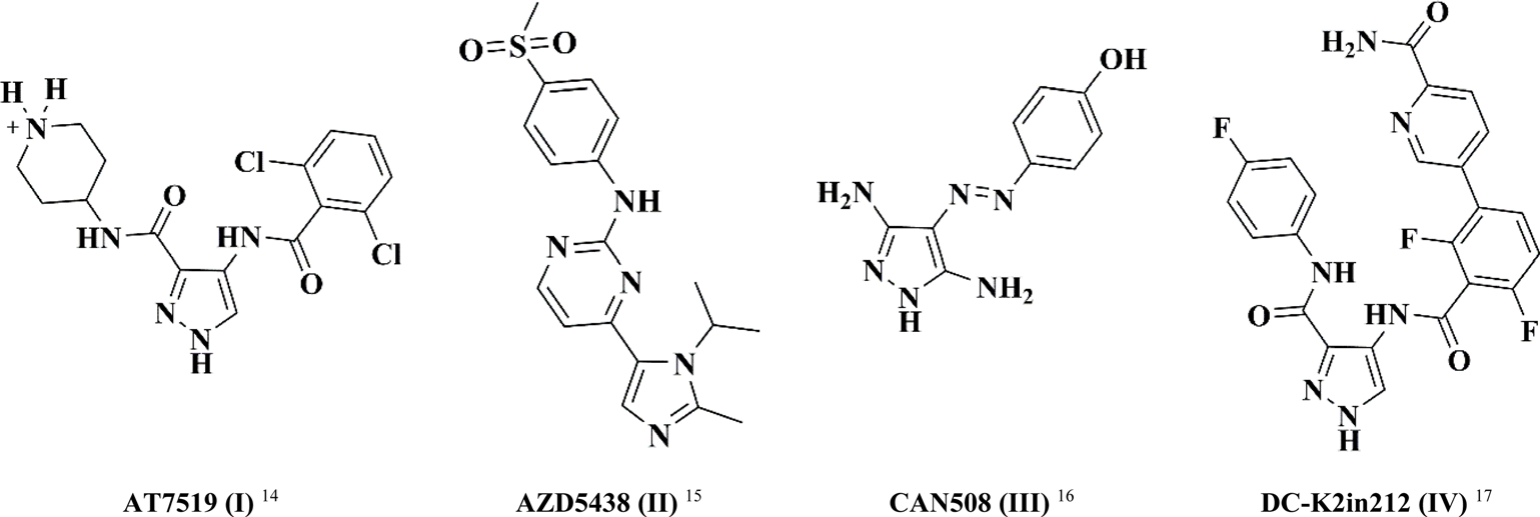
Structure of clinically approved CDK2 inhibitors (I–IV).

In this study, pyrazole was chosen as a fundamental framework for drug exploration. This decision was driven by its molecular diversity, which accounts for the wide range of biological activities observed in different pyrazole derivatives, including antibacterial, anti-inflammatory, and anticancer activities.^14–17^Fig. 1 illustrates a set of differently substituted pyrazole-containing compounds with outstanding CDK inhibitory activity.

AT7519 (I) is a multi CDK inhibitor for CDK 1, 2, 4, 6 and 9 isoforms with IC_50_ values in the range of 10–210 nM.^18^ Interestingly, the imidazo-pyrmidine derivative AZD5438 (II) displayed potent CDK2 inhibition with an IC_50_ value of 6 nM.^19^ Furthermore, the di-amino pyrazole derivative CAN508 (III) exhibited selective inhibition activity against CDK2 with an IC_50_ value of 0.35 μM.^20^ Besides, 4-benzoylamino-1*H*-pyrazole-3-carboxamide derivative DC-K2in212 (IV) displayed 17-fold selectivity against CDK2 over CDK1 with an IC_50_ value of 0.295 μM.^21^

**Fig. 2:**
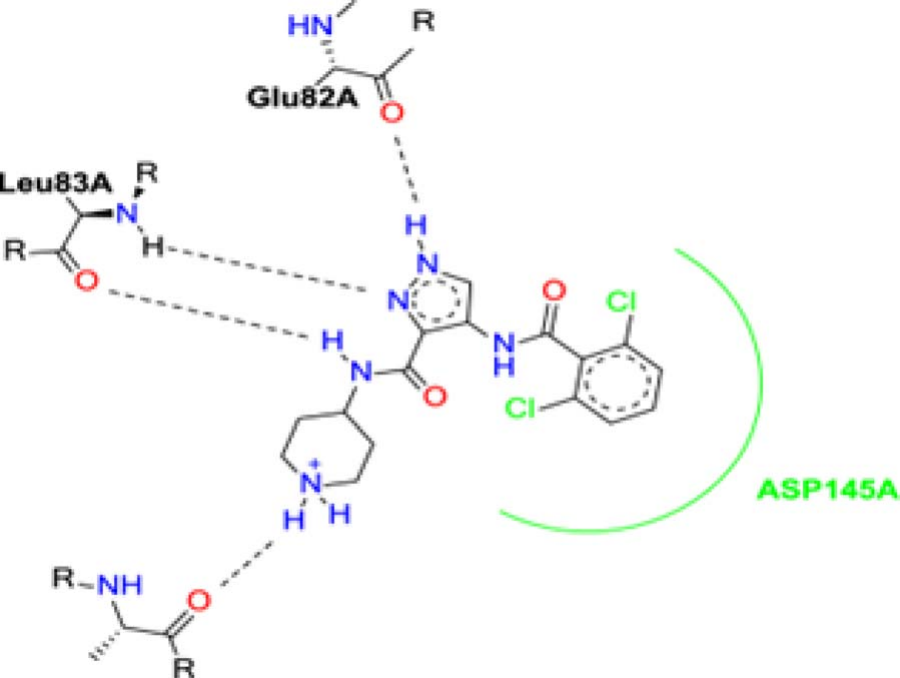
Schematic of the binding mode of the lead compound AT7519 (I) to CDK2 ATP binding site.

The binding mode of AT7519 (I) to the CDK2 ATP binding site disclosed the importance of the pharmacophoric pyrazole nucleus to occupy the adenine region of the ATP binding pocket. The H-bonding interactions mediated by the pyrazole core NH with Glu82 anchored AT7519 (I) tightly into the CDK2 hinge region. Both the carboxamide side chain N atom and pyrazole N displayed two H-bonding with Leu83. The 2,6-dichlorobenzamide moiety exhibited hydrophobic interactions with the Asp145 residue, as shown in Fig. 2.

Herein, the design of the target compounds with a pyrazole core, *i.e.*, 2a–g, 4, 7a–d, 8a–b, 9 and 10, was derived from the structure optimization of the reference compound AT7519 (I) based on its reported structure–activity relationship (SAR), as follows.^18^

**Fig. 3:**
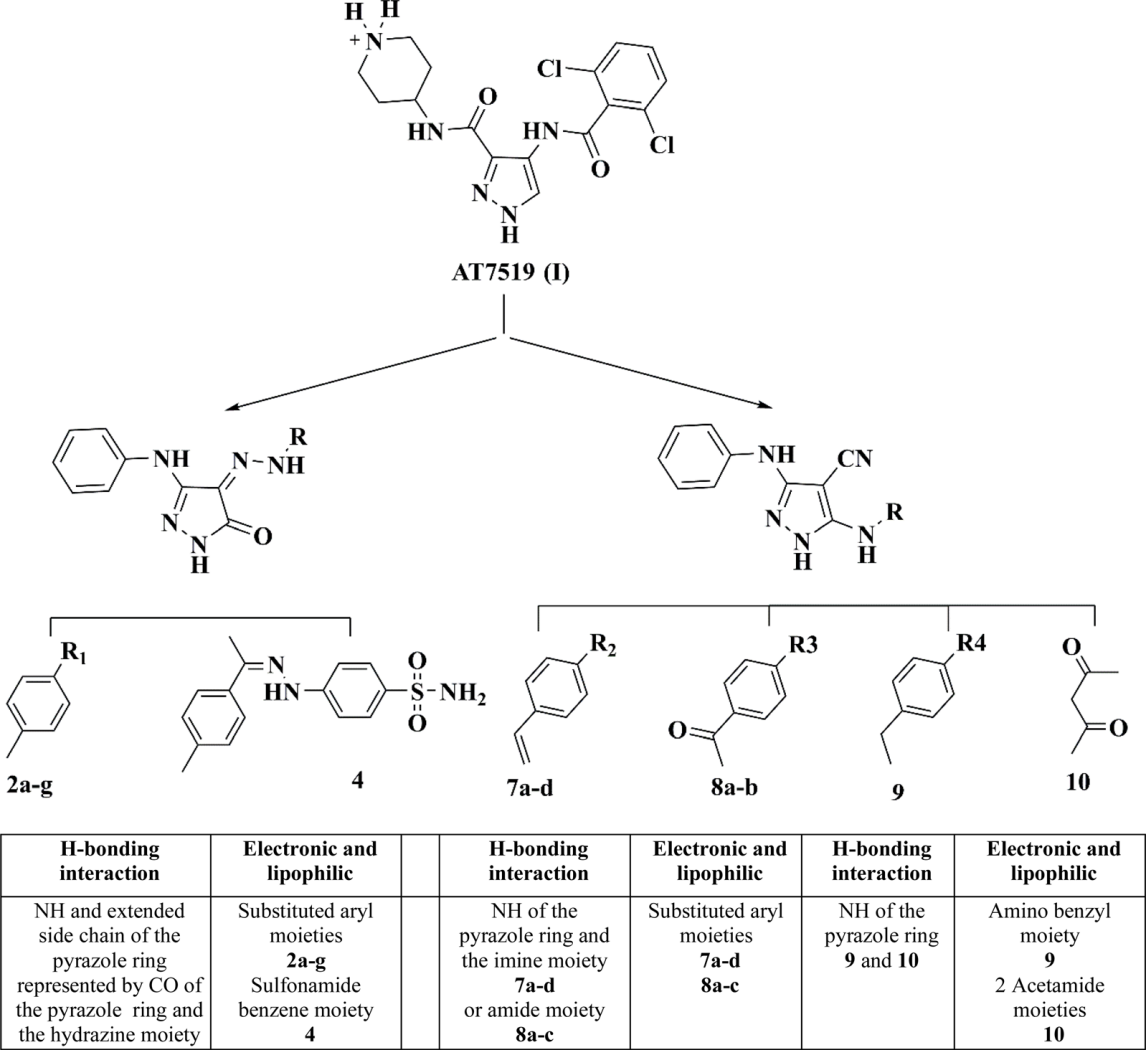
Similarities of the pharmacophoric features between AT7519 (I) and the target compounds.

The pharmacophoric pyrazole core in the lead compound, which fills the adenine region of the ATP binding pocket is preserved. The H-bonding and hydrophobic interaction of our lead compound AT7519 (I) was furnished by the functional groups illustrated in Fig. 3.

Consequently, a nitrile group was introduced at position 4 in compounds 7a–d, 8a and b, 9, and 10 to afford hydrogen bond acceptor functionality and form non-specific dipole interactions with the amino acid residues, promoting their binding with the sterically occluded protein kinases, and consequently enhancing their activity. The phenyl amino group at position 3 of the pyrazole ring acts as a hydrogen bond donor and provides occupation of a small hydrophobic pocket.

Enzyme inhibition *versus* CDK2 was performed for all the compounds. Furthermore, the targeted compounds were screened for their anticancer activity *versus* 60 human cancer cell lines from the NCI-USA. The most promising compounds were subjected to further assessment at five doses against a full NCI 60 cell panel assay. A molecular modeling study was also performed to explore their possible binding modes within the CDK2/ATP binding site. The active analogs were further investigated for their dynamic stability through a dynamic simulation process. Eventually, the ADMET computational parameters were assessed to predict the drug-likeness characteristics of the target compounds.

## 2. Results and discussion

### 2.1. Chemistry

**Scheme 1:**
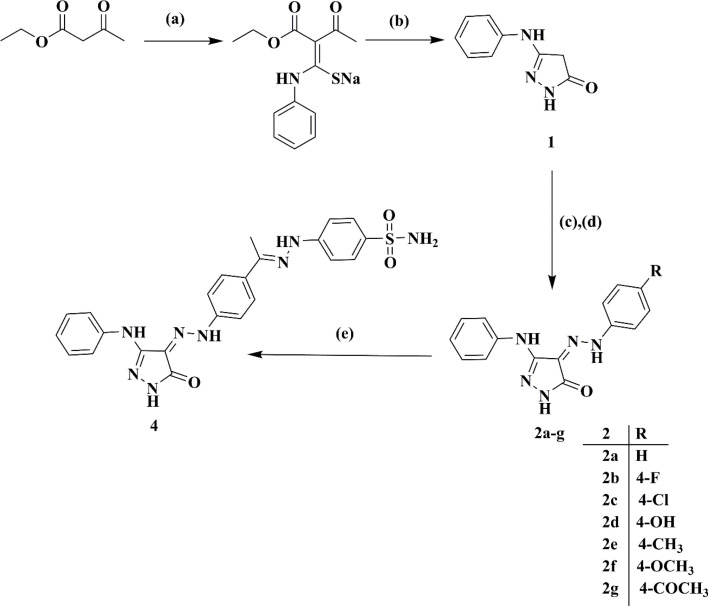
Synthetic route for target compounds 2a–g and 4. Reagents and conditions: (a) Na, CH_3_OH, phenyl isothiocyanate, reflux, 30 min, cool to 50 °C. (b) Hydrazine hydrate, reflux, 1 h. (c) Ar–NH_2_, HCl, sodium nitrite solution, stirring, 0–5 °C, 15 min. (d) Diazonium salt, 2 N HCl, sodium acetate, stirring, RT, 24 h. (e) 2g, 4-hydrazineylbenzenesulfonamide 3, C_2_H_5_OH, acetic acid, stirring, RT, 24 h.

Ethyl acetoacetate was reacted with phenyl isothiocyanate in a solution of sodium methylate, followed by cyclization using hydrazine hydrate, resulting in 5-phenylamino-2,4-dihydro-pyrazol-3-one 1.^22^ Subsequently, the target 4-(2-(4-un/substitutedphenyl)hydrazono)-5-(phenylamino)-2,4-dihydro-3*H*-pyrazol-3-ones 2a–g were obtained *via* the coupling of 1 with the respective diazonium salts of different aromatic amines, as shown in Scheme 1.^23^

The sulfonamide diazonium salt was reduced using a mild reducing agent, stannous chloride, to the corresponding 4-hydrazineylbenzenesulfonamide 3. The latter was condensed with acetyl derivative 2g (generated from Scheme 1) in glacial acetic acid to attain pyrazole benzene sulfonamide derivative 4.^24^

**Scheme 2:**
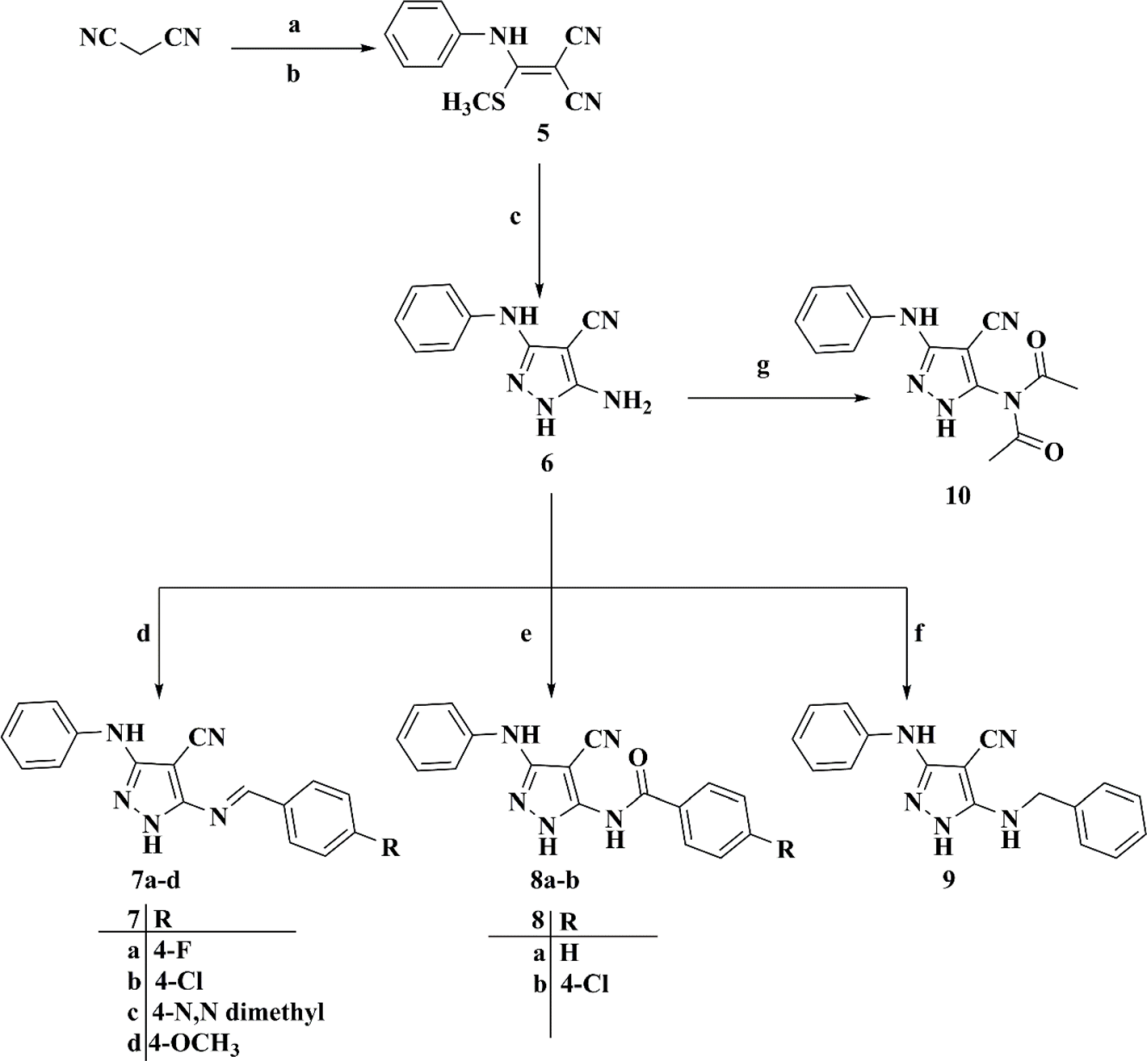
Synthetic route for target compounds 7a–d, 8a and b, 9, and 10. Reagents and reaction conditions: (a) KOH, DMF, phenyl isothiocyanate, stirring, RT, 24 h. (b) Dimethyl sulphate, stirring, RT, 8 h. (c) Hydrazine hydrate, W. B., 3–4 h. (d) Ar–CHO, acetic acid, reflux, 5 h. (e) Ar–CO–Cl, N(CH_2_CH_3_)_3_, dry benzene, stirring, RT, 6 h. (f) Ar–CH_2_–Cl, anhydrous K_2_CO_3_, dry benzene, reflux, 6 h. (g) (CH_3_CO)_2_O, acetic acid, stirring, RT, 24 h.

The synthesis of 5-((4-un/substitutedbenzylidene)amino)-3-(phenylamino)-1*H*-pyrazole-4-carbonitriles 7a–d was initiated *via* the preparation of the key 2-((methylthio)(phenylamino)methylene)malononitrile intermediate 5 by the nucleophilic addition reaction of C2 of malononitrile to phenyl isothiocyanate, followed by the methylation reaction using dimethyl sulfate.^25,26^ Subsequently, the latter was cyclized directly upon the fusion with hydrazine hydrate to afford the key intermediate 5-amino-3-phenylamino-1*H*-pyrazole-4-carbonitrile 6,^27^ which was refluxed with different aromatic aldehydes in glacial acetic acid to afford the final target compounds 7a–d, as illustrated in Scheme 2.^28^

Remarkably, 4-substituted-*N*-(4-cyano-3-(phenylamino)-1*H*-pyrazol-5-yl)benzamides 8a and b were attained by the treatment of 5-amino-3-(phenylamino)-1*H*-pyrazole-4-carbonitrile 6 with the appropriate benzoyl chloride derivative with the aid of triethylamine as a catalyzing agent in dry benzene.^29,30^ Curiously, the reaction of compound 6 with benzyl chloride produced 5-(benzylamino)-3-(phenylamino)-1*H*-pyrazole-4-carbonitrile 9, whereas the reaction of our intermediate of interest 6 with a catalytic amount of acetic anhydride in acetic acid produced *N*-acetyl-*N*-(4-cyano-3-(phenylamino)-1*H*-pyrazol-5-yl)acetamide 10, as illustrated in Scheme 2.^31^

The spectroscopic characteristics of compounds 2a–g, 7a–d, 8a and b, 9, and 10 confirmed their structures. In the case of compounds 2a–g, the IR spectrum of compound 2d revealed a broad OH band at the *v* max of 3479 cm^−1^, while the ^1^H-NMR spectra of compounds 2e, 2f and 2g revealed singlet bands corresponding to the Ar–CH_3_, Ar–OCH_3_ and Ar–COCH_3_ protons at *δ* of 2.31, 3.78, 2.58 ppm, respectively. The ^13^C-NMR spectra of compounds 2f and 2g showed bands located at *δ* of 55.86 and 27.06 for Ar–OCH_3_ and Ar–CH_3_, respectively. The IR spectrum of compound 4 revealed bands for the expected new SO_2_ group at 1338 and 1177 cm^−1^, respectively. The ^1^H-NMR spectrum of compound 4 confirmed the appearance of a singlet band for the aliphatic protons of CH_3_ at *δ* of 4.39 ppm, protons of Ar–CH in the *δ* range of 6.93–7.9 ppm and identical singlet peak at *δ* of 6.94 ppm for the NH_2_ protons. The ^1^H NMR spectra of compounds 7a–d showed a characteristic singlet signal assigned to the (N=CH) proton, which appeared in the *δ* range of 8.75–9.01 ppm. Compound 7c showed a singlet signal at *δ* of 3.88 for OCH_3_, where compound 7d revealed a singlet signal at *δ* of 3.07 ppm for the 6 protons of 2CH_3_. Alternatively, the ^1^H NMR spectra of compounds 8a and b revealed bands for NH and CO. Compound 8b showed a singlet signal at *δ* of 3.89 for the OCH_3_ protons. The ^1^H NMR spectrum of compound 9 showed a singlet signal assigned to the benzyl proton at *δ* of 4.51 ppm. Compound 10 showed 2 extra CO bands. In contrast, compound 10 exhibited 2 singlet signals for the acetyl protons at 2.19 and 2.62 pm. Finally, the mass spectra of all the compounds were harmonized with their calculated molecular weights.

### 2.2. Biological screening

#### 2.2.1. *In vitro* cyclin-dependent kinase 2/cyclin A2 enzyme inhibition assay

**Fig. 4:**
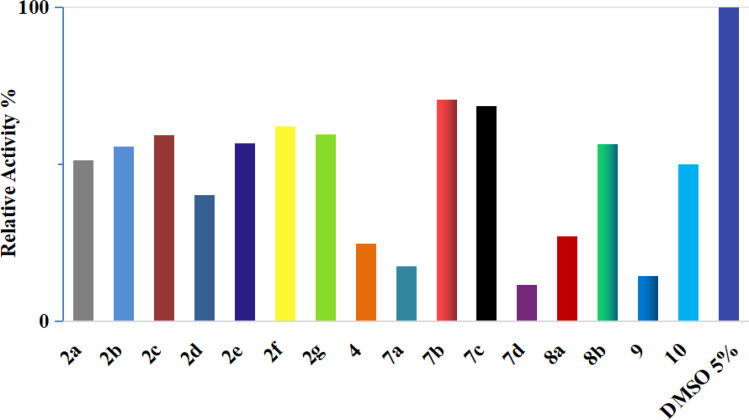
Inhibition of CDK2/cyclin A2 by compounds 2a–g, 4, 7a–d, 8a and b, 9, and 10 at a concentration of 50 μM.

**Fig. 5:**
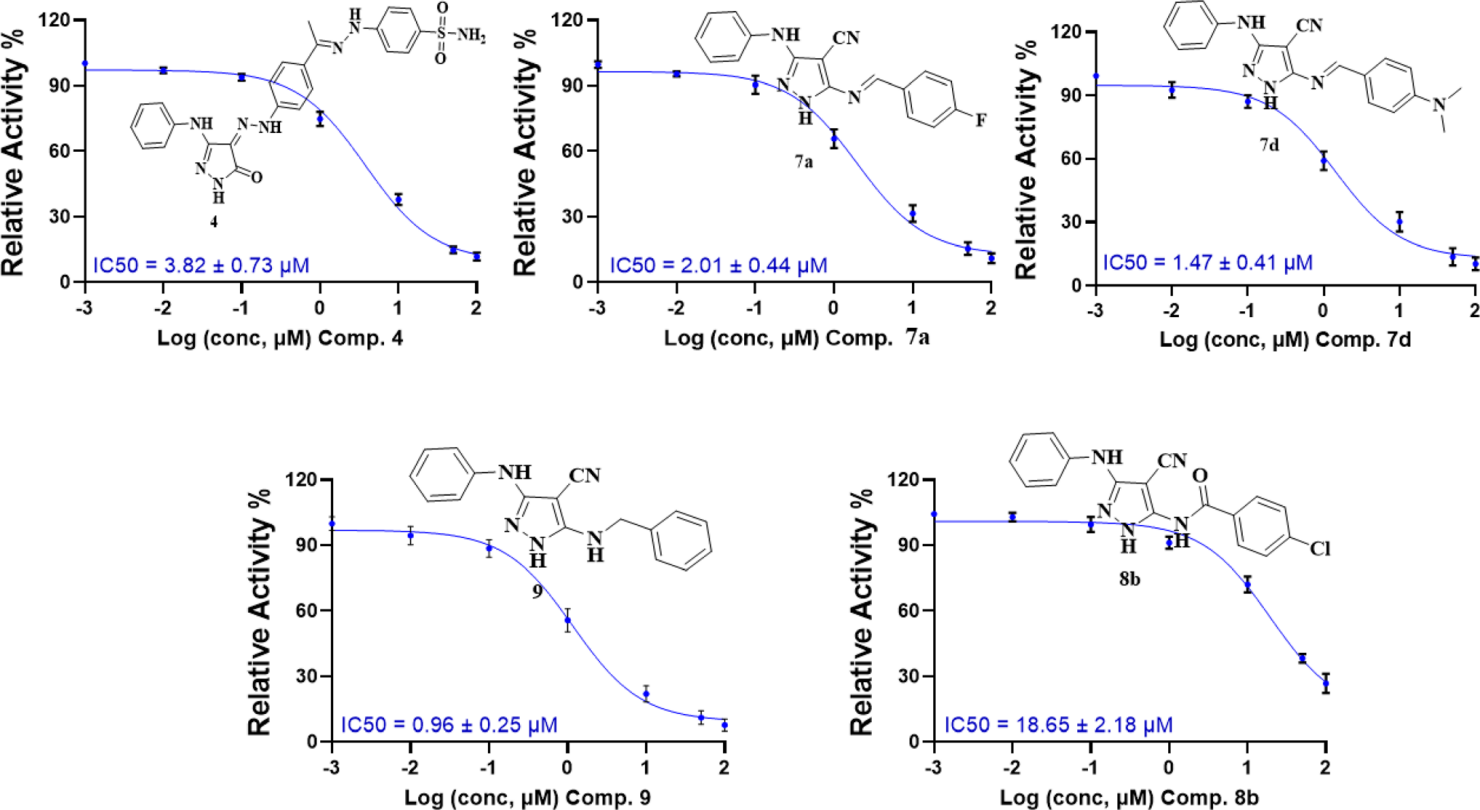
Dose–response curves with the inhibitory concentration at 50% (IC_50_) of compounds 4, 7a, 7d, 8b, and 9 using the CDK2/cyclin A2 protein kinase activity assay.

The studied pyrazoles 2a–g, 4, 7a–d, 8a and b, 9, and 10 were assessed for their *in vitro* CDK2/cyclin A2 enzyme inhibition assay at a concentration of 50 μM using the Promega CDK2/cyclinA2 kinase enzyme system coupled with the ADP-Glo assay.^32,33^ The percentage enzyme activity was the lowest for five compounds, namely 4, 7a, 7d, 8a, and 9 with values of 24.7%, 17.5%, 11.5%, 27.1% and 14.5%, respectively. Following that, the IC_50_ profiling of the promising CDK2/cyclin A2 inhibitors was tested (*n* = 3), which revealed that compound 9 has the best IC_50_ against CDK2 of 0.96 μM. Alternatively, compounds 7a and 7d showed IC_50_ values of 2.01 and 1.47 μM, respectively. Finally, compound 4 showed an IC_50_ value of 3.82 μM and compound 8b exhibited moderate activity with an IC_50_ value of 18.65 μM, as shown in Fig. 4 and 5, respectively.

These results are consistent with the recent CDK2 findings,^10–13^ confirming the anticipated inhibitory potential of the selected pyrazole derivatives. The observed IC_50_ values for compounds 9, 7a, 7d, and 4, particularly their strong activity against CDK2/cyclin A2, validate the structural modifications introduced during the design phase, further supporting their promising role as CDK2 inhibitors.

#### 2.2.2. *In vitro* anticancer activity against 60 cell lines

The newly synthesized pyrazoles 2a–g, 7a–d, 8a and b, 9, and 10 were submitted to the National Cancer Institute (NCI) Developmental Therapeutics Program (http://www.dtp.nci.nih.gov) for assessment of their potential antiproliferative activity against a panel of NCI 60 cancer cell lines. All compounds were approved for screening against the approved 60 cancer cell lines at a single dose of 10 μM. The human cancer cell lines were derived from 9 variant cancer types including leukemia, melanoma, lung, colon, CNS, ovarian, renal, prostate and breast cancers. The results are presented as percentage of growth inhibition (GI%) of the screened compounds against the full panel of cell lines. The mean GI% of the screened compounds against the NCI 60 cell lines showed the remarkable activity of two compounds, 4 and 9, with the mean GI of 96.47% and 65.90%, respectively, as shown in Table 1.

**Table 1:** NCI *in vitro* testing results of compounds 4 and 9 with their corresponding GI% in μM

Panel line	Cell line	GI% compound 4	GI% compound 9
Leukemia	CCRF-CEM	96.41	81.65
	RPMI-8226	96.53	—
Melanoma cell lines	UACC-257	94.37	—
	SK-MEL-2	—	79.33
	MALME-3M	—	78.10
Breast cell lines	T-47D	—	82.76
Non-small cell lung cancer cell lines	NCI-H23	99.80	—
	NCI-H322M	90.78	—
	NCI-H460	91.13	—
Colon cancer cell lines	HT29	95.46	—
	KM12	96.31	—
	HCT-116	99.70	—
CNS cancer cell lines	SF-268	90.67	—
	SF-295	94.88	—

A deep look at the results revealed that some derivatives of 2a–g revealed pronounced anticancer activity against some breast cancer cell lines such as compounds 2a, 2b and 2c, which showed activity against MCF7 with GI% of 73.8%, 86.1% and 77.1%, respectively. Furthermore, analog 2b exhibited comparable anticancer activity against MDA-MB-468 with GI% of 72.25%. Alternatively, compound 7c demonstrated remarkable activity against the MCF7 breast cancer cell line with GI% of 79.41%. The achieved results revealed the influence of electronic characteristics of the side chain substituents on the anticancer activity of sulfonamide candidate 4. Therefore, the electron-withdrawing-substituted derivatives 2b and 2c and unsubstituted analog 2a exhibited pronounced anticancer activity against the breast cancer cell line. Alternatively, changing the electronic environment of the substituents to electron-donating groups led to a decrease in the anticancer activity, as seen in compounds 2d, 2e, and 2f with GI% mean of 27.12%, 6.87% and 10.24%, respectively.

#### 2.2.3. Five dose full NCI 60 cell panel assay

Owing to the promising preliminary screening results, compound 4 was remarkably chosen for assessment at an additional five doses (0.01, 0.1, 1, 10, and 100 μM). The response parameters against several cell lines (GI_50_, TGI and LC_50_) were calculated and presented in Table 2, where GI_50_ indicates the compound concentration that causes a 50% decrease in the net cell growth, TGI (total growth inhibition) represents the cytostatic activity, and LC_50_ (lethal dose 50) represents the compound concentration that causes net 50% loss of the initial cells.^34^

**Table 2:** NCI *in vitro* testing of compound 4 at five dose levels (GI_50_, TGI, and LC_50_ in μM)

Panel line	Cell line	GI_50_ (μM)	TGI (μM)	LC_50_ (μM)
Leukemia	CCRF-CEM	2.63	8.92	>100
	HL-60(TB)	2.86	0.171	60.5
	K-562	2.29	6.37	22.8
	MOLT-4	1.64	5.24	34.9
	RPMI-8226	3.36	28.2	>100
	SR	1.74	5.33	23.6
Non-small cell lung cancer	A549/ATCC	19.4	>100	>100
	EKVX	5.39	26.9	>100
	HOP-62	2.05	6	30.3
	HOP-92	1.3	6.55	30.7
	NCI-H226	3.31	12.3	35.1
	NCI-H23	2.54	6.53	23.10
	NCI-H322M	3.72	15.2	41.9
	NCI-H460	3.21	11.10	45.3
	NCI-H522	2.17	5.25	74.8
Colon cancer	COLO 205	3.28	12	34.6
	HCC-2998	2.29	6.08	21.3
	HCT-116	3.04	10.3	32
	HCT-15	5.29	18.4	42.9
	HT29	3.58	11.6	34
	KM12	2.66	8.95	29.9
	SW-620	3.18	12.6	58.8
CNS cancer	SF-268	2.22	6.23	31.5
	SF-295	3.67	13.6	38.7
	SF-539	1.58	3.68	8.56
	SNB-19	2.59	8.76	29.6
	U251	1.88	3.76	7.51
Melanoma	LOX IMVI	2.13	4.33	8.81
	MALME-3M	1.98	3.86	7.49
	M14	2.83	7.55	35.1
	MDA-MB-435	2.07	5.03	17.8
	SK-MEL-2	2.11	4.38	9.1
	SK-MEL-28	1.91	3.65	6.99
	SK-MEL-5	3.32	12.8	35.8
	UACC-257	3.97	16.7	75.5
	UACC-62	1.96	4.34	9.6
Ovarian cancer	IGROV1	4.5	17.6	43.2
	OVCAR-3	1.87	4.54	12.5
	OVCAR-4	2.84	9.26	40.7
	OVCAR-5	11	24.4	54.2
	OVCAR-8	2.59	7.81	29
	NCI/ADR-RES	>100	>100	>100
	SK-OV-3	2.34	6.47	23.7
Renal cancer	786-0	2.14	6.27	24.7
	A498	11.4	32.8	94.7
	ACHN	5.9	18.5	43
	CAKI-1	12.5	25.1	50.1
	RXF 393	1.1	3.88	21.1
	SN12C	2.38	6.58	23.5
	TK-10	18.6	56.8	>100
	UO-31	19.4	>100	>100
Prostate cancer	PC-3	3.06	11.3	35.8
	DU-145	3.21	10.4	32.2
Breast cancer	MCF7	2.4	7.34	27.4
	MDA-MB-231/ATCC	2	4.07	8.28
	HS 578T	3.31	19.4	>100
	BT-549	2.83	9.66	31.1
	T-47D	2.22	5.92	23.9
	MDA-MB-468	1.4	3	6.41

In addition, the mean graph midpoints (MG-MID) for the subpanel and full panel were calculated for the GI_50_ to demonstrate the average activity parameter for each compound. Commonly, compound 4 revealed obvious anticancer activity against almost the whole panel of cancer cell lines with GI_50_ values in the range of 1.1–5.9 μM. Remarkably, compound 4 displayed noticeable cytostatic activity against the following cell lines: leukemia (HL-60(TB); TGI 0.171 μM), breast cancer (MDA-MB-468; TGI 3 μM), melanoma (SK MEL-28; TGI 3.65 μM, MALME-3M: TGI 3.86 μM), CNS cancer (SF-539; TGI 3.68 μM and U251; TGI 3.76 μM), and renal cancer (RXF 393; TGI 3.88 μM).

Compound 4 exerted obvious antiproliferative activity against almost the whole NCI panel with a full panel GI_50_ (MGMID) value of 3.81 μM and subpanel GI_50_ (MG-MID) range of 2.6–9.17 μM. Among the tested cancer subpanels, breast cancer, CNS, leukemia, melanoma cancer, prostate cancer and colon cancer were the most susceptible subpanels to compound 4 with GI_50_ (MG-MID) values of 2.36, 2.388, 2.42, 2.47, 3.13 and 3.33 μM, respectively, as shown in Table 3.

**Table 3:** Mean graph mid-point values (MG-MID^a^) for the GI_50_ parameter (μM) of the subpanel cancer cell lines

Subpanel type	MG-MID	Selectivity index^c^
Leukemia	2.42	1.574
Non-small cell lung cancer	4.787778	0.795
Colon cancer	3.331429	1.143
CNS cancer	2.388	1.595
Melanoma	2.475556	1.539
Ovarian cancer	4.19	0.909
Renal cancer	9.1775	0.415
Prostate cancer	3.135	1.215
Breast cancer	2.36	1.614
Full panel MG-MID^b^ = 3.81		

a MG-MID is the average activity parameter over individual subpanels/tested compound.

b Full panel MG-MID is the average sensitivity over all cell lines (full panel)/tested compound.

c Selectivity index was attained by dividing the full panel MG-MID (μM) for each compound by its individual subpanel MG-MID (μM).

#### 2.2.4. *In vitro* cytotoxicity against normal cells

The *in vitro* cytotoxicity of the most potent compounds 4 and 9 was evaluated against WI-38 normal cells using staurosporine as the reference standard. Compounds 4 and 9 demonstrated minimal cytotoxic effects on normal cells, with IC_50_ values of 41.16 and 56.78 μM, respectively compared to the reference standard IC_50_ value of 28.87 μM. These findings suggest that the newly synthesized compounds exhibit significantly lower cytotoxicity against normal cells, approximately half that observed in the cancer cell lines.

#### 2.2.5. Flow cytometry cell cycle analysis and apoptosis study

**Fig. 6:**
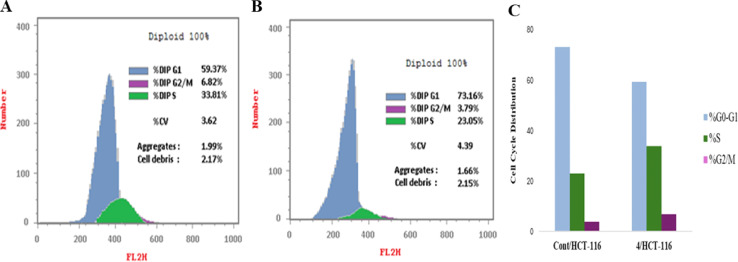
Flow cytometric analysis for cell cycle distribution. (A) Control HCT-116, (B) compound 4, and (C) graphical representation for cell cycle distribution analysis among differently treated cells.

**Fig. 7:**
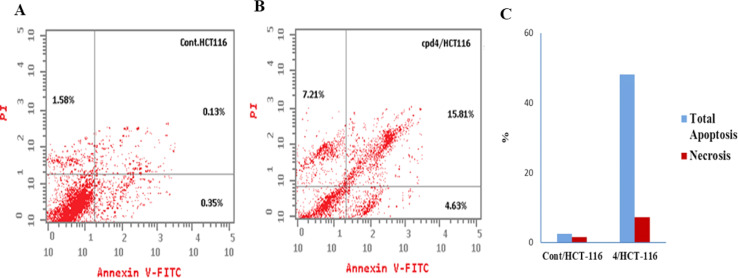
Flow cytometric analysis of apoptosis among treated cells. (A) Control HCT-116, (B) compound 4, and (C) graphical illustration of apoptosis% among differently treated cells.

Cell cycle analysis was conducted to explore the mechanism and the mode of action of the newly synthesized compounds as anticancer agents, typically inducing cytotoxicity by activating signaling pathways that trigger apoptosis.^35^ The MTT cell viability assays were conducted using the mutant HCT-116 cell line, revealing that compounds 4 and 9 displayed potent cytotoxic effects, with IC_50_ values of 1.806 and 10.003 μM, respectively. Compound 4 was further subjected to cell cycle analysis following the established protocols.^35^ The flow cytometry results presented in Table 4 demonstrate that compound 4 decreased the percentage of cells in the G2/M phase from 6.82% to 3.79% and in the S phase from 33.81% to 23.05%, while increasing the percentage of cells in the G0/G1 phase from 59.37% to 73.16% compared to the control. The control cells exhibited a typical pattern of DNA content representing the pre-G1, S, G2/M, and G0/G1 phases of the cell cycle, as shown in Fig. 6. Conversely, the cells treated with compound 4 displayed an apoptosis pattern of DNA content, characterized by G0/G1 and G2/M phases, indicating apoptotic cell growth arrest occurring at the G1 phase, as shown in Table 5 and Fig. 7.

**Table 4:** Flow cytometric analysis for cell cycle distribution of compound 4 on HCT-116 cells

Comp.	DNA content results				
	Conc. μM	%G0/G1	%S	%G2/M	Results
Control	7.022	59.37	33.81	6.82	—
Comp. 4	1.806	73.16	23.05	3.79	Cell growth arrest at G1

**Table 5:** Effect of compound 4 on apoptosis in HCT-116 cells

Comp	Apoptosis				
	Conc. μM	Total	Early	Late	Necrosis
Control	7.022	2.06	0.35	0.13	1.58
Comp. 4	1.806	27.65	4.63	15.81	7.21

#### 2.2.6. Gel electrophoresis and immuno-blot analysis of proteins (western blot)

**Fig. 8:**
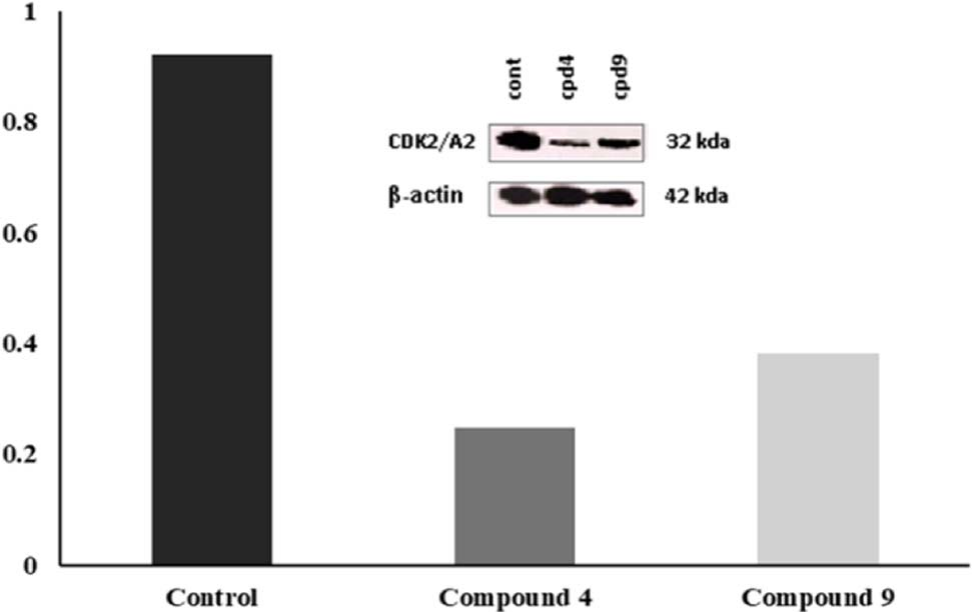
Western blot assay for compounds 4 and 9.

The method of sodium dodecyl sulfate polyacrylamide gel electrophoresis (SDS-PAGE) is employed to separate proteins based on their size. When combined with western blotting (immunoblotting), both techniques are commonly used to identify the presence and/or relative abundance of a specific protein within a sample containing a complex mixture of proteins.^36^ In this process, each sample's total protein content is loaded onto a gel and separated by electrophoresis, allowing proteins to migrate through the gel matrix under the influence of an electric current. To facilitate protein migration, the proteins are initially denatured and given a negative charge by exposure to a detergent like SDS. A molecular weight marker with known-sized bands assists in identifying proteins of interest. Once the protein components are adequately separated, they can be transferred onto a polyvinylidene fluoride (PVDF) membrane *via* electrophoresis, where they migrate out of the gel and onto the membrane. To detect a specific protein on the membrane, a primary antibody targeting that protein is added to form a protein–antibody complex, followed by the addition of a secondary antibody that binds to the complex *via* its antibody side. Typically, the secondary antibody is linked to an enzyme that generates luminescence upon reacting with its substrate. The intensity of the luminescence, directly correlating with the amount of protein that interacted with the antibody, is captured by a Bio-Rad Imager.^36^ HCT-116 cells were treated with either 3 μM of compound 4 and 1 μM of compound 9 and incubated 48 h. Treatment with compounds 4 and 9 resulted in inhibitions in the expression of CDK-2 compared to the vehicle treated cells that suggest their potential cellular inhibitory effect Fig. 8.

### 2.3. *In silico* studies

#### 2.3.1. Molecular docking study

The docking patterns of compounds 4, 7a, and 9 were explored in detail, revealing the topmost CDK2 inhibition activity with IC_50_ values of 4.2, 3.98, and 0.97 μM, respectively. Therefore, the X-ray crystal structure of CDK2 co-crystallized with AT7519 (I) (ref. 19) was downloaded from PDB (PDB code: 2VU3) (http://www.rcsb.org/).

**Fig. 9:**
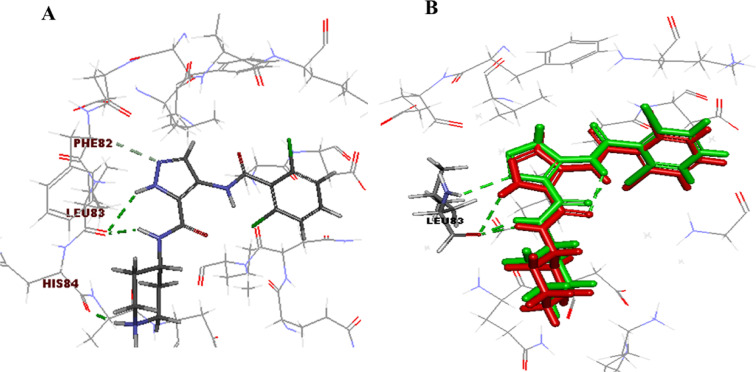
2D ligand AT7519 (I) interaction diagram (A) and 3D representation (B) of superimposition of the native ligand (green) and its re-docked pose (red) in CDK2 ATP binding site.

The initial step of docking protocol was re-docking of the co-crystallized ligand to assess validation parameters as RMSD = 0.5227 Å, CDOCKER energy = – 12.8047 kcal mol^−1^. Furthermore, the following step was the identification of binding mode of the native ligand which confirmed the validity of the applied docking protocol. Two H bonds with the essential Leu83 residue were mediated *via* the pyrazole core N atom and side chain NH. In addition to an extra hydrogen bond formed between His84 residue and NH piperidine. The 2,6-dichlorobenzamide moiety occupied almost all of the ATP binding region, whereby the pyrazole moiety accommodated the adenine region, as shown in Fig. 9.

Successively, we applied the same docking protocol for our compounds of interest. Most of the docked compounds presented a similar binding pattern to that of the reference AT7519 (I).

**Fig. 10:**
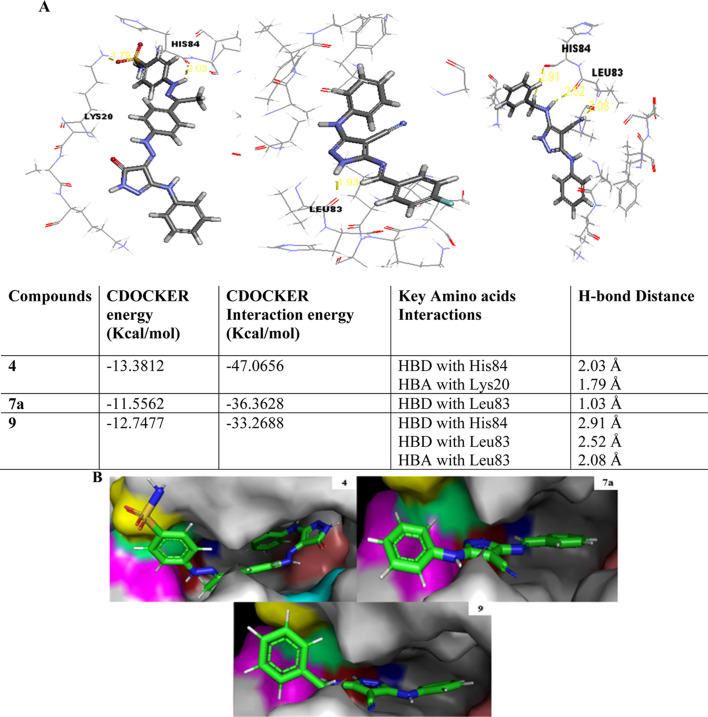
Interaction diagrams presenting the best synthesized compounds 4, 7a and 9 in the binding site of the CDK2. (A) 2D representation showing H-bonding and bond distance with CDOCKER and CDOCKER interaction energies values and (B) 3D representation where yellow: Lys20, blue: Glu82, red: Leu83, pink: His84, green: Phe82, cyan: Gln131, and salmon: Asp145.

As shown in Fig. 10, the most potent CDK2 inhibitors 4 (IC_50_ of 4.2 μM), 7a (IC_50_ of 3.98 μM), and 9 (IC_50_ of 0.97 μM) could mediate the essential interactions with the enzyme binding site, in particular the H-bonding with the Leu83 residue *via* the C≡N group, phenylamino NH groups and the pyrazole NH; an observation that can rationalize their obtained activity. In contrast, docking of compound 4 (IC_50_ of 4.2 μM) was significantly different from the reference AT7519 (I), given that it demonstrated H-bonding between Lys20 and NH_2_SO_2_. The aforementioned binding pattern for compound 4 can explain its observed inhibitory activity.

#### 2.3.2. Molecular dynamic simulation

**Fig. 11:**
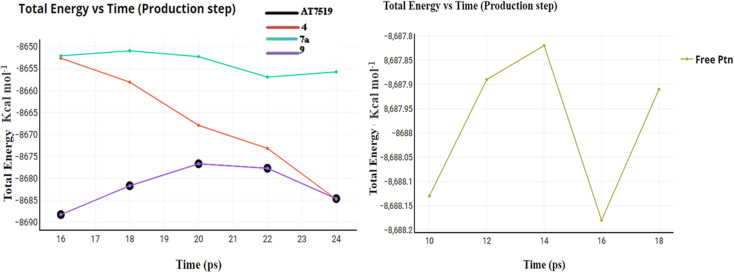
Total energy *versus* time for compounds AT7519 (I), 4, 7a and 9 and the free protein itself.

**Fig. 12:**
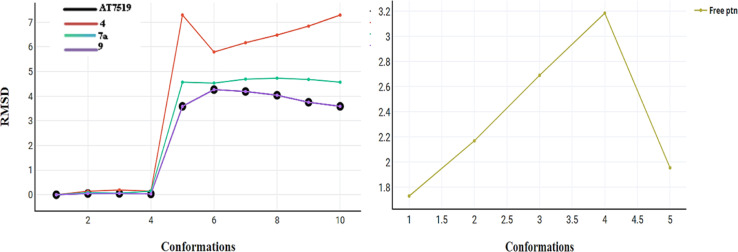
RMSD for compounds AT7519 (I), 4, 7a and 9 and the free protein itself.

**Fig. 13:**
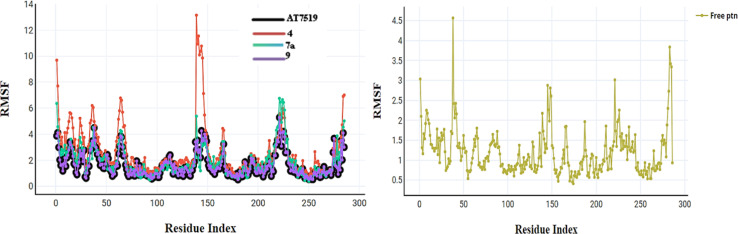
RMSF for compounds AT7519 (I), 4, 7a, and 9 and the free protein itself.

The primary goal of the dynamic simulation and trajectory analysis is to determine whether the interaction between the docked molecules and CDK2 is stable or not, which affects their inhibition. The best active conformer of reference AT7519 (I) obtained from the docking study was selected for its dynamic stability inside the CDK2 active site and compared to that of the best active compounds 4, 7a, and 9. Fig. 11 shows the total energy *versus* the time in the range of 16–24 ps for the compounds upon interaction. The energy levels fluctuate between −8.820 and −8.785 (kcal mol^−1^), presenting their stable and preferred mode of interaction. The root mean square deviation (RMSD) of the docked complex measures how frequent the positions of the atoms in the drug-CDK2 complex change over time compared to their initial positions, as shown in Fig. 12. Here, the RMSD values are in the range of 0–4.25, except for compound 4 from 0–7.5, suggesting that the complex remains relatively stable with only minor deviations. This is a sign of a well-preserved interaction. Furthermore, the root mean square fluctuations (RMSF) were used to assess the flexibility during this simulation, as shown in Fig. 13. In this case, low RMSF values indicate stringent binding, which means that the interaction between the docked molecules and CDK2 is tight and inhibitory. In summary, in this study, dynamics simulation and various analyses were performed to demonstrate that the newly synthesized compounds form a stable and preferred interaction with CDK2, which inhibits its activity, suggesting their potential therapeutic implications.

#### 2.3.3. Ramachandran plot

**Fig. 14:**
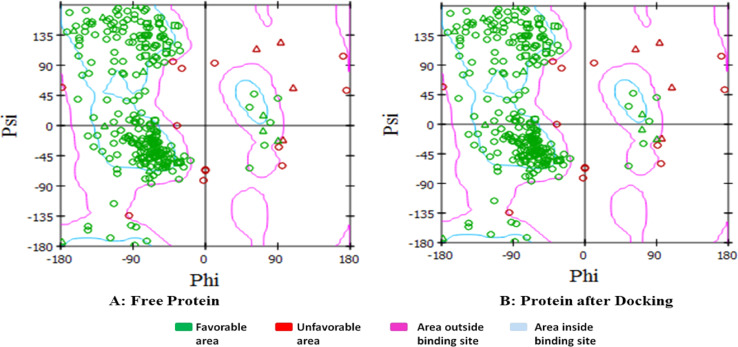
Ramachandran plot presenting the torsional energy conformations for interaction between the newly synthesized molecules and CDK2, where (A) represents the free protein before docking while (B) represents the protein after docking.

The verification of the predicted torsion angles within the targeted receptor was done using the Ramachandran plot. Discovery Studio 4.1 was used before and after docking to authorize the interaction of our newly synthesized compounds in the correct binding sites and reveal the topological changes applied to the CDK2 protein. The conventional terms used to represent the torsion angles on either sides of the α carbons in peptides could be represented by the low energy conformations for *φ* (phi) and *ψ* (psi). The graphical representation in Fig. 14 displays the same number of favorable green areas during interaction with the docked molecules compared to the free protein, and thus ease of multiple conformations within the binding sites of the protein.

#### 2.3.4. *In silico* ADMET study

##### 2.3.4.1 Pharmacokinetic and drug-likeness prediction

Herein, we used the SwissADME web server (http://www.swissadme.ch/) to predict the drug-likeness and pharmacokinetics parameters of the novel compounds 2a–g, 7a–d, 8a and b, 9 and 10. In this work, we aimed to define the relationship between the chemical structure of our compounds of interest and definite parameters including blood–brain barrier (BBB) permeation, human gastrointestinal absorption (HIA), substrate or non-substrate for the permeability glycoprotein (P-gp), log kp and interaction of molecules with cytochrome P450 isomers (CYP). The results are presented using the “BOILED-EGG chart (2D plot between the calculated TPSA and log *P* properties of the target molecule). Thus, the GIT passive absorption probability, BBB penetration, effluxing by P-gp (PGP+) and no effluxing *via* P-gp (PGP−) are indicated as white area, yellow region, blue dots, and red dot, respectively.^37^

**Fig. 15:**
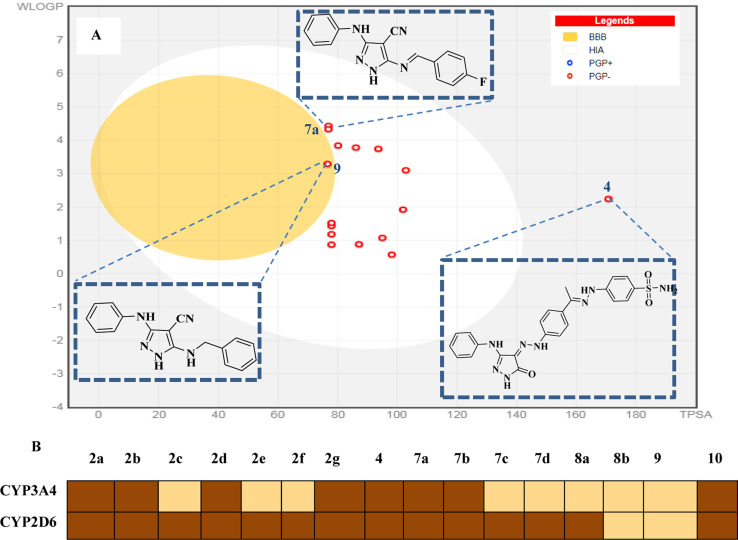
Pharmacokinetic profile. (A) Boiled-egg diagram of all the synthesized compounds. White region: high probability of passive absorption by the gastrointestinal tract; yellow region (yolk): high probability of brain penetration. Yolk and the white region are not mutually exclusive. Bluepoint: P-gp substrate. Redpoint: no P-gp substrate. (B) Inhibitory capacity on the set of CYP450 enzymes.

Most of the target compounds are predicted to have no drug–drug interactions upon administration with no activity on cytochrome P450 isomers (CYP3A2 and CYP2D6).^38^ The BOILED-Egg charts of the target compounds exhibited minimal CNS adverse effects, GIT passive absorption probability and no BBB permeability predicting. In addition, there is no possibility of tumor cell lines resistance through the efflux mechanism to the target compounds given that they may not be substrates for P-gp (PGP−).^39^ Additionally, the target molecules are expected to have good bioavailability with score = 0.55 according to their five rule-based filters compliance^40^ including Lipinski,^41^ Ghose,^42^ Veber,^43^ Egan^44^ and Muegge rules,^45^ as shown in Fig. 15.

##### 2.3.4.2 Toxicity prediction

The probable toxicities such as mutagenicity, carcinogenicity, tumorigenicity and teratogenicity were predicted by subjecting our compounds of interest to another virtual filter (Osiris Property Explorer (http://www.organic-chemistry.org/prog/peo/)). This online program compares the target compounds with *in vitro* and *in vivo* studied compounds within its database based on their functional group similarity. The color-coded results are red, green and yellow. Red color predicts high probability of toxicity, while yellow indicates moderate toxicity and green color means low toxic potential, as shown in Table 6.^46,47^ The results exhibited that some of the studied compounds are predicted to be safe and displayed low or no toxicity concerning tumorigenicity, mutagenicity, irritant effect and effect on the reproductive system.

**Table 6:** Computer-aided Osiris property explorer of all the studied compounds

Comp.	Probable toxicities
2a	High risk for irritant effect
	High risk of mutagenicity
	Moderate toxic effects on the reproductive system
2b	High risk for irritant effect
	Moderate toxic effects on the reproductive system
2c	High risk for irritant effect
	Moderate toxic effects on the reproductive system
2d	High risk for irritant effect
	Moderate toxic effects on the reproductive system
2e	High risk for irritant effect
	Moderate toxic effects on the reproductive system
2f	High risk for irritant effect
	Moderate tumorigenicity
	Moderate toxic effects on the reproductive system
2g	High risk of mutagenicity
	High risk for irritant effect
	Moderate toxic effects on the reproductive system
4	High risk for irritant effect
	Moderate toxic effects on the reproductive system
7a	Moderate risk of mutagenicity
7b	Moderate risk of mutagenicity
7c	Moderate risk of mutagenicity
7d	Moderate risk of mutagenicity
	High tumorigenicity
8a	Moderate risk of mutagenicity
8b	Moderate risk of mutagenicity
9	Moderate risk of mutagenicity
10	Moderate risk of mutagenicity
	Moderate tumorigenicity

## 3. Conclusion

The newly synthesized pyrazole derivatives 2a–g, 7a–d, 8a and b, 9, and 10 were assessed for their CDK2 inhibitory activity, and among them compounds 4, 7a, and 9 were found to be promising CDK2 inhibitors (IC_50_ of 4.2, 3.98, and 0.97 μM, respectively). All the compounds were subjected to full panel screening for their anticancer activity against 60 cancer cell lines by NCI/USA. Two compounds, 4 and 9, displayed promising growth inhibitory activity with mean GI% of 96.47 and 65.90, respectively. The NCI five dose assay of compound 4 exhibited its remarkable anticancer activity against almost the full panel (GI_50_ range: 1.1–5.9 μM) and (full panel GI_50_ (MG-MID) of 3.81 μM). Comparable binding interactions with the co-crystallized ligand AT7519 (I) (PDB code: 2VU3) were noticed for the docked compounds in the CDK2 ATP binding site, especially binding with the Leu83 residue. Finally, the synthesized compounds showed good ADMET properties and toxicity profiles. As a future plan, these proposed compounds will serve as targeted scaffolds that require further optimization to enhance their therapeutic potential, including improving their selectivity, efficacy, and pharmacokinetic properties.

## 4. Experimental

### 4.1. Chemistry

The starting materials, reagents and solvents were purchased from Sigma-Aldrich (USA) or Alfa-Aesar Organics and used as received without further purification. The reactions were monitored by analytical thin layer chromatography (TLC), which was performed on pre-coated (0.25 mm) silica gel GF254 plates (E. Merck, Germany), and the compounds were detected with 254 nm UV lamp. Silica gel (60–230 mesh) was employed for routine column chromatography separation. Melting points (°C) were determined using the open capillary tube method on a Stuart SMP30 apparatus and are uncorrected. Mass spectroscopy was carried out on a direct inlet part to the mass analyzer in a Thermo Scientific GCMS model ISQ at the Regional Center for Mycology and Biotechnology (RCMB), Al-Azhar University, Nasr City, Cairo. ^1^H NMR and ^13^C NMR spectra were recorded using a Bruker AVANCE III 400 MHz High-performance Digital FT-NMR spectrometer (Bruker Corporation, Germany) at the Microanalytical Unit, Faculty of Pharmacy, Cairo University and Mansoura University. ^1^H/^13^C NMR spectra were run at 400/100 MHz, respectively, in DMSO-*d*6 as the solvent, and chemical shifts are quoted in *δ* as parts per million (ppm) downfield from tetramethylsilane (TMS) as the internal standard. Infrared spectra were determined using a Shimadzu Fourier-transform infrared spectrometer (IR-470, Shimadzu, Kyoto, Japan). All spectra were expressed as *v* cm^−1^. Compounds 1,^22^5,^25,26^3 (ref. 48) and 6 (ref. 27) were synthesized according to the reported methods.

#### 4.1.1. General procedure for the synthesis of 4-(2-(4-un/substitutedphenyl)hydrazono)-5-(phenylamino)-2,4-dihydro-3*H*-pyrazol-3-ones 2a–g

A solution of the appropriate aromatic amines (2.56 mmol) in 2 N hydrochloric acid (3 mL) was cooled to 0–5 °C in an ice bath. Subsequently, a solution of sodium nitrite (0.18 g, 2.56 mmol) in water (3 mL) was added dropwise while stirring. This mixture was stirred for 15 min at the same temperature. Next, a solution of 5-phenylamino-2,4-dihydro-pyrazol-3-one 1 (0.44 g, 2.56 mmol) in hydrochloric acid (2 N; 6 mL) was added while stirring. The solution was adjusted to neutral pH using sodium acetate and left to stir for 18–24 h at room temperature. The separated solid was collected, dried and crystallized from ethanol to give compounds 2a–g.^23^

##### 4.1.1.1 5-(Phenylamino)-4-(2-phenylhydrazono)-2,4-dihydro-3*H*-pyrazol-3-one 2a

Dark-brick red powder; yield (83%); M. P.: 215–217 °C; IR: (*v* max, cm^−1^): 3460 (NH), 3150 (NH), 3050 (NH), 1598 (CO); ^1^H-NMR (400 MHz, DMSO-*d*_6_) *δ*: 6.91 (t, 1H, *J* = 8, Ar–H), 7.17 (t, 1H, *J* = 8, Ar–H), 7.29 (t, 2H, *J* = 6, Ar–H), 7.44 (t, 2H, *J* = 8, Ar–H), 7.69 (d, 2H, *J* = 8, Ar–H), 7.76 (d, 2H, *J* = 8, Ar–H), 8.81 (s, 1H, NH, exchangeable by D_2_O), 11.10 (s, 1H, NH, exchangeable by D_2_O), 13.06 (s, 1H, NH, exchangeable by D_2_O); MS: (M. wt: 279.30): *m*/*z*, 279.11 (M^+^, [100%]); anal. calcd for C_15_H_13_N_5_O; C, 64.51; H, 4.69; N, 25.07; found: C, 64.21; H, 4.75; N, 25.3.

##### 4.1.1.2 4-(2-(4-Fluorophenyl)hydrazono)-5-(phenylamino)-2,4-dihydro-3*H*-pyrazol-3-one 2b

Dark-brown powder; yield (82%); M. P.: 239–240 °C; IR: (*v* max, cm^−1^): 3417 (NH), 3178 (NH), 3163 (NH), 1662 (CO); ^1^H-NMR (400 MHz, DMSO-*d*_6_) *δ*: 6.92 (t, 1H, *J* = 6, Ar–H), 7.30 (dd, 4H, *J* = 6.6, Ar–H), 7.73–7.78 (m, 4H, Ar–H), 8.80 (s, 1H, NH, exchangeable by D_2_O), 11.08 (s, 1H, NH, exchangeable by D_2_O), 13.05 (s, 1H, NH, exchangeable by D_2_O); MS: (M. wt: 297.29): *m*/*z*, 297.1 (M^+^, [100%]); anal. calcd for C_15_H_12_FN_5_O; C, 60.60; H, 4.07; N, 23.56; found: C, 60.43; H, 4.29; N, 23.72.

##### 4.1.1.3 4-(2-(4-Chlorophenyl)hydrazono)-5-(phenylamino)-2,4-dihydro-3*H*-pyrazol-3-one 2c

Dark-brick red powder; yield (86%); M. P.: 249–251 °C; IR: (*v* max, cm^−1^): 3450 (NH), 3250 (NH), 3190 (NH), 1672 (CO); ^1^H-NMR (400 MHz, DMSO-*d*_6_) *δ*: 6.93 (t, 1H, *J* = 8, Ar–H), 7.31 (t, 2H, *J* = 6, Ar–H), 7.49 (d, 2H, *J* = 12, Ar–H), 7.75 (d, 4H, *J* = 8, Ar–H), 8.82 (s, 1H, NH, exchangeable by D_2_O), 11.10 (s, 1H, NH, exchangeable by D_2_O), 13.00 (s, 1H, NH, exchangeable by D_2_O); MS: (M. wt: 313.74): *m*/*z*, 313.39 (M^+^, [25.3%]); anal. calcd for C_15_H_12_ClN_5_O; C, 57.42; H, 3.86; N, 17.91; found: C, 57.60; H, 3.97; N, 22.18.

##### 4.1.1.4 4-(2-(4-Hydroxyphenyl)hydrazono)-5-(phenylamino)-2,4-dihydro-3*H*-pyrazol-3-one 2d

Dark-red powder; yield (83%); M. P.: 257–259 °C; IR: (*v* max, cm^−1^): 3479 (OH), 3417 (NH), 3259 (NH), 3147 (NH), 1666 (CO); ^1^H-NMR (400 MHz, DMSO-*d*_6_) *δ*: 6.83 (d, 2H, *J* = 8, Ar–H), 6.89 (t, 1H, *J* = 6, Ar–H), 7.28 (t, 2H, *J* = 8, Ar–H), 7.52 (d, 2H, *J* = 12, Ar–H), 7.73 (d, 2H, *J* = 8, Ar–H), 8.71 (s, 2H, NH, exchangeable by D_2_O), 9.59 (s, 1H, OH, exchangeable by D_2_O), 11.00 (s, 1H, NH, exchangeable by D_2_O); MS: (M. wt: 295.30): *m*/*z*, 295.88 (M^+^, [104.6%]); anal. calcd for C_15_H_13_N_5_O_2_; C, 61.01; H, 4.44; N, 23.72; found: C, 60.89; H, 4.67; N, 23.9.

##### 4.1.1.5 5-(Phenylamino)-4-(2-(*p*-tolyl)hydrazono)-2,4-dihydro-3*H*-pyrazol-3-one 2e

Reddish-brown powder; yield (86%); M. P.: 251–253 °C; IR: (*v* max, cm^−1^): 3302 (NH), 3151 (NH), 3024 (NH), 1670 (CO);^1^H-NMR (400 MHz, DMSO-*d*_6_) *δ*: 2.31 (s, 3H, CH_3_), 6.89 (d, 1H, *J* = 3.86, Ar–H), 7.23–7.30 (m, 3H, Ar–H), 7.49 (d, 1H, *J* = 7.86, Ar–H), 7.58 (d, 1H, *J* = 8, Ar–H), 7.75 (d, 1H, *J* = 8, Ar–H), 8.78 (s, 1H, Ar–H), 9.17 (s, 1H, Ar–H), 10.37 (s, 1H, NH, exchangeable by D_2_O), 11.07 (s, 1H, NH, exchangeable by D_2_O), 13.08 (s, 1H, NH, exchangeable by D_2_O); MS: (M. wt: 293.32): *m*/*z*, 293.13 (M^+^, [100%]); anal. calcd for C_16_H_15_N_5_O; C, 65.52; H, 5.15; N, 23.88; found: C, 65.7; H, 5.34; N, 24.09.

##### 4.1.1.6 4-(2-(4-Methoxyphenyl)hydrazono)-5-(phenylamino)-2,4-dihydro-3*H*-pyrazol-3-one 2f

Dark-brick red powder; yield (86%); M. P.: 240–242 °C; IR: (*v* max, cm^−1^): 3302 (NH), 3143 (NH), 3055 (NH), 1666 (CO); ^1^H-NMR (400 MHz, DMSO-*d*_6_) *δ*: 3.78 (s, 3H, OCH_3_), 6.90 (t, 1H, *J* = 8, Ar–H), 7.02 (d, 2H, *J* = 8, Ar–H), 7.28 (t, 2H, *J* = 6, Ar–H), 7.65 (d, 2H, *J* = 8, Ar–H), 7.75 (d, 2H, *J* = 8, Ar–H), 8.75 (s, 1H, NH, exchangeable by D_2_O), 11.03 (s, 2H, NH, exchangeable by D_2_O); ^13^C-NMR (100 MHz, DMSO-*d*_6_) *δ*: 55.86, 115.12, 115.45, 117.53, 117.61, 117.91, 118.15, 120.93, 121.69, 129.1, 129.35, 135.76, 141.33, 146.22, 157.25, 158.71; MS: (M. wt:309.32): *m*/*z*, 309.12 (M^+^, [100%]); anal. calcd for C_16_H_15_N_5_O_2_; C, 65.3; H, 4.79; N, 19.04; found: C, 62.35; H, 5.01; N, 22.87.

##### 4.1.1.7 4-(2-(4-Acetylphenyl)hydrazono)-5-(phenylamino)-2,4-dihydro-3*H*-pyrazol-3-one 2g

Dark-reddish brown powder; yield (85%); M. P.: 265–267 °C; IR: (*v* max, cm^−1^): 3363 (NH), 3163 (NH), 3055 (NH), 1666 (CO), 1566 (CO);^1^H-NMR (400 MHz, DMSO-*d*_6_) *δ*: 2.58 (s, 3H, Ar–COCH_3_), 6.91 (t, 1H, *J* = 6, Ar–H), 7.17 (t, 2H, *J* = 8, Ar–H), 7.29 (t, 2H, *J* = 6, Ar–H), 7.44 (t, 2H, *J* = 8, Ar–H), 7.69 (d, 1H, *J* = 8, Ar–H), 7.76 (d, 1H, *J* = 8, Ar–H), 8.91 (s, 1H, NH, exchangeable by D_2_O), 11.18 (s, 1H, NH, exchangeable by D_2_O), 13.06 (s, 1H, NH, exchangeable by D_2_O);^13^C-NMR (100 MHz, DMSO-*d*6) *δ*: 27.06, 115.63, 115.82, 118.15, 118.65, 121.31, 125.4, 125.82, 129.17, 129.5, 130.41, 133.02, 141.01, 146.05, 146.14, 158.15, 197; MS: (M. wt: 321.33): *m*/*z*, 321.72 (M^+^, [21.8%]); anal. calcd for C_17_H_15_N_5_O_2_; C, 63.54; H, 4.71; N, 21.79; found: C, 63.72; H, 4.89; N, 22.04.

#### 4.1.2. Synthesis of 4-(2-(1-(4-(2-(5-oxo-3-(phenylamino)-1,5-dihydro-4*H*-pyrazol-4-ylidene)hydrazinyl)phenyl)ethylidene)hydrazinyl)benzene sulfonamide 4

To a solution of 4-(2-(4-acetylphenyl)hydrazono)-5-(phenylamino)-2,4-dihydro-3*H*-pyrazol-3-one 2g (1.55 g, 10 mmol) in absolute ethanol (30 mL), a solution of 4-hydrazineylbenzenesulfonamide 3 (0.164 g, 10 mmol) in glacial acetic acid was added and the reaction mixture was stirred for 24 h at room temperature. Subsequently, the mixture was concentrated under reduced pressure and poured onto ice-cold water. The separated solid was filtered, washed with water, dried and crystallized from ethanol to give the title compound 4.^24^

Dark-brick red powder; yield (86%); M. P.: 249–251 °C; IR: (*v* max, cm^−1^): 3420 (NH), 3320 (NH), 3190 (NH), 1663 (CO), 1177 and 1338 (SO_2_); ^1^H-NMR (400 MHz, DMSO-*d*_6_) *δ*: 4.39 (t, 1H, *J* = 4.8, CH_3_), 6.93 (t, 1H, Ar–H), 6.94 (s, 2H, NH_2_, exchangeable by D_2_O), 7.30 (d, 2H, *J* = 8.4, Ar–H), 7.34 (d, 2H, *J* = 5.6, Ar–H), 7.69 (d, 2H, *J* = 8.8, Ar–H), 7.73 (d, 2H, *J* = 8.8, Ar–H), 7.77 (d, 2H, *J* = 8, Ar–H), 7.90 (d, 2H, *J* = 8.8, Ar–H), 8.84 (s, 1H, NH, exchangeable by D_2_O), 9.77 (s, 1H, NH, exchangeable by D_2_O), 11.12 (s, 1H, NH, exchangeable by D_2_O), 13.14 (s, 1H, NH, exchangeable by D_2_O); ^13^C-NMR (100 MHz, DMSO-*d*6) *δ*:13.56, 112.45, 115.97, 118.05, 121.13, 123.48, 127.03, 127.75, 129.15, 134.03, 135.58, 141.21, 141.98, 143.19, 146.15, 149.00, 158.54; MS: (M. wt: 490.54): *m*/*z*, 490.13 (M^+^, [50%]); anal. calcd for C_23_H_22_N_8_O_3_S; C, 56.31; H, 4.52; N, 22.84; S, 6.54; found: C, 56.47; H, 4.65; N, 23.19; S, 6.62.

#### 4.1.3. General procedure for the synthesis of 5-((4-un/substitutedbenzylidene)amino)-3-(phenylamino)-1*H*-pyrazole-4-carbonitriles 7a–d

A mixture of 5-amino-3-(phenylamino)-1*H*-pyrazole-4-carbonitrile 6 (0.39 g, 2 mmol) and the appropriate aromatic aldehyde (2 mmol) in glacial acetic acid (5 mL) was refluxed for 4–5 h, the solvent was removed under vacuum and the residue was crystallized from ethanol^28^ to give the titled compounds 7a–d.

##### 4.1.3.1 5-((4-Fluorobenzylidene)amino)-3-(phenylamino)-1*H*-pyrazole-4-carbonitrile 7a

Dark-yellow powder; yield (81%); M. P.: 265–267 °C; IR: (*v* max, cm^−1^): 3317 (NH), 3190 (NH), 2222 (CN); ^1^H-NMR (400 MHz, DMSO-*d*_6_) *δ*: 6.88–6.90 (m, 1H, Ar–H), 7.27 (t, 3H, *J* = 8, Ar–H), 7.43 (t, 3H, *J* = 8, Ar–H), 8.06–8.09 (m, 2H, Ar–H), 9.00 (s, 1H, imine-CH), 13.13 (s, 2H, NH, exchangeable by D_2_O); ^13^C-NMR (75 MHz, DMSO-*d*6) *δ*:114.66, 116.73, 116.95, 117.1, 120.96, 129.37, 132.24, 132.33, 142.24, 164.05, 166.55, 217.59; MS: (M. wt: 305.31): *m*/*z*, 305.11 (M^+^, [100%]); anal. calcd for C_17_H_12_FN_5_; C, 66.88; H, 3.96; N, 22.94; found: C, 66.71; H, 4.15; N, 23.17.

##### 4.1.3.2 5-((4-Chlorobenzylidene)amino)-3-(phenylamino)-1*H*-pyrazole-4-carbonitrile 7b

Orange powder; yield (80%); M. P.: 273–275 °C; IR: (*v* max, cm^−1^): 3344 (NH), 3190 (NH), 2218 (CN); ^1^H-NMR (400 MHz, DMSO-*d*_6_) *δ*: 6.89–6.91 (m, 1H, Ar–H), 7.27 (t, 3H, *J* = 6, Ar–H), 7.67 (d, 2H, *J* = 8, Ar–H), 8.02 (d, 2H, *J* = 8, Ar–H), 9.01 (s, 2H, imine-CH + Ar–H), 13.18 (s, 2H, NH, exchangeable by D_2_O); ^13^C-NMR (100 MHz, DMSO-*d*6) *δ*: 114.63, 117.12, 129.4, 129.81, 131.34, 131.68, 134.24; MS: (M. wt: 321.76): *m*/*z*, 321.08 (M^+^, [100%]), 323.08 (M^+2^, [33.9%]); anal. calcd for C_17_H_12_ClN_5_; C, 63.46; H, 3.76; N, 21.77; found: C, 63.19; H, 3.84; N, 21.98.

##### 4.1.3.3 5-((4-Methoxybenzylidene)amino)-3-(phenylamino)-1*H*-pyrazole-4-carbonitrile 7c

Yellow powder; yield (85%); M. P.: 233–235 °C; IR: (*v* max, cm^−1^): 3332 (NH), 3190 (NH), 2214 (CN); ^1^H-NMR (400 MHz, DMSO-*d*_6_) *δ*: 3.88 (s, 3H, OCH_3_), 6.86–6.88 (m, 1H, Ar–H), 7.14 (d, 3H, *J* = 8, Ar–H), 7.26 (t, 2H, *J* = 8, Ar–H), 7.49 (s, 1H, NH, exchangeable by D_2_O), 7.95 (d, 2H, *J* = 8, Ar–H), 8.91 (s, 2H, imine-CH + Ar–H), 13.05 (s, 1H, NH, exchangeable by D_2_O); ^13^C-NMR (100 MHz, DMSO-*d*6) *δ*: 56.13, 114.94, 115.14, 115.73, 116.52, 116.98, 120.78, 127.97, 129.04, 129.37, 130.01, 131.9, 132.37, 142.24, 143.02, 163.66, 164.71, 192.02; MS: (M. wt: 317.34): *m*/*z*, 317.03 (M^+^, [11.3%]); anal. calcd for C_18_H_15_N_5_O; C, 68.13; H, 4.76; N, 22.07; found: C, 68.4; H, 4.91; N, 22.33.

##### 4.1.3.4 5-((4-(Dimethylamino)benzylidene)amino)-3-(phenylamino)-1*H*-pyrazole-4-carbonitrile 7d

Pale yellow powder; yield (83%); M. P.: 284–285 °C; IR: (*v* max, cm^−1^): 3317 (NH), 3205 (NH), 2214 (CN); ^1^H-NMR (400 MHz, DMSO-*d*_6_) *δ*: 3.07 (s, 6H, 2CH_3_), 6.83 (d, 3H, *J* = 8, Ar–H), 7.23–7.25 (m, 2H, Ar–H, NH, exchangeable by D_2_O), 7.51–7.52 (m, 2H, Ar–H), 7.78 (d, 2H, *J* = 8, Ar–H), 8.71–8.75 (m, 1H, imine-CH + Ar–H), 12.85 (s, 1H, NH, exchangeable by D_2_O); ^13^C-NMR (100 MHz, DMSO-*d*6) *δ*: 70.42, 111.54, 112.04, 115.33, 116.57, 116.76, 120.00, 122.33, 128.93, 129.08, 131.88, 142.82, 152.93, 154.01, 154.97, 164.71; MS: (M. wt: 330.39): *m*/*z*, 330.16 (M^+^, [100%]); anal. calcd for C_19_H_18_N_6_; C, 69.07; H, 5.49; N, 25.44; found: C, 68.79; H, 5.62; N, 25.63.

#### 4.1.4. General procedure for the synthesis of 4-substituted-*N*-(4-cyano-3-(phenylamino)-1*H*-pyrazol-5-yl)benzamides 8a and b

A mixture of 5-amino-3-(phenylamino)-1*H*-pyrazole-4-carbonitrile 6 (0.39 g, 2 mmol) and the appropriate benzoyl chloride derivative (2 mmol) and triethylamine (2 mmol) in dry benzene (5 mL) was stirred at room temperature for 6 h, and then the solvent was removed under vacuum, finally crystalizing the residue from ethanol to give the title compounds 8a and b.^29,30^

##### 4.1.4.1 4-Chloro-*N*-(4-cyano-3-(phenylamino)-1*H*-pyrazol-5-yl)benzamide 8a

Faint-yellow powder; yield (84%); M. P.: 169–171 °C; IR: (*v* max, cm^−1^): 3429 (NH), 3309 (NH), 3244 (NH), 2214 (CN), 1689 (CO); ^1^H-NMR (400 MHz, DMSO-*d*_6_) *δ*: 6.88 (t, 1H, *J* = 6, Ar–H), 7.22 (t, 2H, *J* = 8, Ar–H), 7.54 (d, 2H, *J* = 8, Ar–H), 7.65 (d, 2H, *J* = 8, Ar–H), 8.06 (d, 2H, *J* = 8, Ar–H), 8.19 (s, 2H, NH, exchangeable by D_2_O), 8.98 (s, 1H, NH, exchangeable by D_2_O); MS: (M. wt: 337.76): *m*/*z*, 337.07(M^+^, [100%]), 339.07 (M^2+^, [33.9%]); anal. calcd for C_17_H_12_ClN_5_O; C, 60.45; H, 3.58; N, 20.73; found: C, 60.74; H, 3.8; N, 20.99.

##### 4.1.4.2 *N*-(4-Cyano-3-(phenylamino)-1*H*-pyrazol-5-yl)-4-methoxybenzamide 8b

Yellow powder; yield (85%); M. P.: 233–235 °C; IR: (*v* max, cm^−1^): 3390 (NH), 3302 (NH), 3244 (NH), 2214 (CN), 1666 (CO); ^1^H-NMR (400 MHz, DMSO-*d*_6_) *δ*: 3.89 (s, 3H, OCH_3_), 6.89 (t, 1H, *J* = 8, Ar–H), 7.10 (d, 2H, *J* = 8, Ar–H), 7.24 (d, 2H, *J* = 8, Ar–H), 7.59 (d, 2H, *J* = 8, Ar–H), 8.12–8.15 (m, 4H, Ar–H + 2NH, exchangeable by D_2_O), 8.96 (s, 1H, NH, exchangeable by D_2_O); ^13^C-NMR (100 MHz, DMSO-*d*6) *δ*: 56.06, 56.14, 65.06, 113.66, 113.86, 114.24, 116.57, 118, 121.22, 123.62, 124.95, 129.08, 129.43, 133.61, 134.13, 134.23, 141.45, 151.38, 156.73, 163.24, 168.47; MS: (M. wt: 333.34): *m*/*z*, 333.03 (M^+^, [68.9%]); anal. calcd for C_18_H_15_N_5_O_2_; C, 64.86; H, 4.54; N, 21.01; found: C, 64.7; H, 4.72; N, 21.25.

#### 4.1.5. Synthesis of 5-(benzylamino)-3-(phenylamino)-1*H*-pyrazole-4-carbonitrile 9

To a mixture of 5-amino-3-phenylamino-1*H*-pyrazole-4-carbonitrile 6 (0.39 g, 2 mmol) and anhydrous potassium carbonate (0.27 g, 2 mmol) in dry benzene (5 mL), an equimolar amount (0.25 g, 2 mmol) of benzyl chloride was added. The reaction mixture was directly heated at 80 °C for 6 h.^31^ After cooling to room temperature and upon pouring on ice-cold, a dark off white precipitate developed which was separated by filtration, then washed with water and recrystallized from ethanol to give the titled compound 9.

Dark-off white powder; yield (85%); M. P.: 136–137 °C; IR: (*v* max, cm^−1^): 3282 (NH), 3186 (NH), 3028 (NH), 2210 (CN); ^1^H-NMR (400 MHz, DMSO-*d*_6_) *δ*: 4.52 (s, 2H, CH_2_), 5.10 (s, 1H, NH, exchangeable by D_2_O), 6.84–6.90 (m, 2H, Ar–H), 7.05 (d, 1H, *J* = 8, Ar–H), 7.22–7.33 (m, 8H; 7Ar–H, 1H, NH, exchangeable by D_2_O), 8.75 (s, 1H, NH, exchangeable by D_2_O); ^13^C-NMR (100 MHz, DMSO-*d*6) *δ*: 51.2, 52.51, 71.29, 115.26, 116.54, 121.23, 127.47, 127.52, 127.84, 128.25, 128.81, 128.86, 129.67, 137.01, 138.48, 142.65, 147.15, 156.57, 217.59; MS: (M. wt: 289.33): *m*/*z*, 289.69 (M^+^, [106.6%]); anal. calcd for C_17_H_15_N_5_; C, 70.57; H, 5.23; N, 24.21; found: C, 70.68; H, 5.40; N, 24.39.

#### 4.1.6 Synthesis of *N*-acetyl-*N*-(4-cyano-3-(phenylamino)-1*H*-pyrazol-5-yl)acetamide 10

A mixture of 5-amino-3-phenylamino-1*H*-pyrazole-4-carbonitrile 6 (0.39 g, 2 mmol), and catalytic amount of acetic anhydride in acetic acid (2 mL) was stirred at room temperature for 24 h and poured on ice/water.^31^ The separated solid was filtered, washed with water and recrystallized from ethanol to give the titled compound 10.

Off-white powder; yield (87%); M. P.: 173–175 °C; IR: (*v* max, cm^−1^): 3400 (NH), 3310 (NH), 2229 (CN), 1585 (CO), 1558 (CO); ^1^H-NMR (400 MHz, DMSO-*d*_6_) *δ*: 2.19 (s, 3H, CH_3_), 2.62 (s, 3H, CH_3_), 6.89–6.97 (m, 1H, Ar–H), 7.25–7.32 (m, 1H, Ar–H), 7.67 (d, 1H, *J* = 8, Ar–H), 7.99 (s, 1H, Ar–H), 8.92 (s, 1H, Ar–H), 9.12 (s, 1H, NH, exchangeable by D_2_O); 10.80 (s, 1H, NH, exchangeable by D_2_O); ^13^C-NMR (100 MHz, DMSO-*d*6) *δ*: 23.7, 24.1, 65.07, 115.26, 116.54, 121.23, 127.47, 127.52, 127.84, 128.25, 128.81, 128.86, 129.67, 137.01, 138.48, 142.65, 147.15, 156.57, 217.59; MS: (M. wt: 283.29): *m*/*z*, 283.93 (M^+^, [21.4%]); anal. calcd for C_14_H_13_N_5_O_2_; C, 59.36; H, 4.63; N, 24.72; found: C, 59.54; H, 4.75; N, 24.98.

### 4.2. Biological assays

#### 4.2.1. CDK2/cyclin A2 assay

The *in vitro* assay of CDK2/cyclin A2 protein kinase was carried out on all the synthesized compounds. The kinase assay was performed in a 96-well white plate with a reaction volume of 50 μL. The reaction was performed in four steps as follows: 2.5 μL of the test compounds or 5% DMSO and 5 μL of CDK2/cyclin A2 enzyme (1.6 ng) was added, and then incubated at room temperature for 30 min. Then, 5 μL of 50 μM ATP. 0.1 μg μL^−1^ histone H1 was added and incubated for 10 min. The reaction was stopped by adding 12.5 μL of ADP-Glo™ Reagent and incubated for 40 min. Then 25 μL of kinase detection reagent was added to each well and incubated for 60 min before detection with luminescence (Integration time 0.5–1 s).^25,32,33^

#### 4.2.2. *In vitro* anti-proliferative activity

Under sterile conditions, the cell lines were grown in RPMI 1640 media (Gibco, NY, USA) supplemented with 10% fetal bovine serum (Biocell, CA, USA), and 5–10^5^ cell/mL was used to test the growth inhibition activity of the synthesized compounds. The compounds with concentrations ranging from 0.01 to 100 μM were prepared in phosphate buffer saline. Each compound was initially solubilized in dimethyl sulfoxide (DMSO); however, each final dilution contained less than 1% DMSO. Solutions of different concentrations (0.2 mL) were pipetted into the separate well of a microtiter tray in duplicate. Cell culture (1.8 mL) containing a cell population of 6–104 cells per mL was pipetted into each well. Controls, containing only phosphate buffer saline and DMSO at identical dilutions, were also prepared in the same manner. These cultures were incubated in a humidified incubator at 37 °C. The incubator was supplied with 5% CO_2_ atmosphere. After 48 h, the cells in each well were diluted 10 times with saline and counted using a Coulter counter. The counts were corrected for the dilution.^34^

#### 4.2.3. Flow cytometry cell cycle analysis

The cell cycle analysis protocol was performed on compound 4 on HCT-116 cells. This test is based on the content of DNA measured by staining using propidium iodide. Initially, the cells were washed in PBS before being kept at 4 °C for 3 min dropwise using the vortex addition of cold 70% ethanol to avoid cell clumping and to ensure fixation as well. Then, 50 mL of a stock of 100 mg mL^−1^ ribonuclease was added to selectively stain only DNA. Finally, 200 mL of a stock solution of 50 mg mL^−1^ of propidium iodide was added.^35^

#### 4.2.4. Flow cytometric analysis of apoptosis

For the detection of apoptosis in the treated cells, the Annexin V-FITC-apoptosis detection kit (PN IM3546) was used, followed by flow cytometric analysis according to the manufacturer's protocol. In this assay, HCT-116 cells were allowed to grow in a 25 cm^3^ flask until 70–80% confluence. Then, the HCT-116 cells were treated with compound 4 for 48 h, followed by washing with PBS, and suspended in binding buffer. To 100 mL of the cell suspension, 1 mL of annexin V-FITC solution and 5 mL of dissolved PI were added and incubated for 15 min in the dark. Then 400 mL of ice-cold binding buffer was added and mixed gently. The flow cytometric analysis of the percentage of apoptotic cells was performed on a COULTER^®^ EPICS^®^ XL™ Flow Cytometer (USA).^35^

#### 4.2.5. Western blotting

HCT-116 cells were treated with either 3 μM of compound 4 and 1 μM of compound 9 and incubated 48 h. The cells were lysed by 1× RIPA buffer containing protease and phosphatase inhibitors to quantify the protein concentration by BCA protein assay. 20 μg per well was loaded 12% SDSPAGE and the protein was transferred into nitrocellulose membrane 0 and blocked with 5% BSA after that was propped against CDK2 [catalogue no. PA1547] overnight at 4 °C. The membrane was subjected to the corresponding IR-conjugated secondary antibodies. B-actin (catalogue no. P7718S) was used as a loading control. LiCOR Odyssey imager was utilized to take pictures to membrane using ImageJ software to analyze the membrane picture.^36^

#### 4.2.6. Molecular modeling studies

The molecular docking study was carried out using the CDOCKER protocol in the Discovery Studio 4.1 Software. The targeted compounds were docked into the CDK2 active site. The X-ray crystallographic structure of CDK2 complexed with AT7519 (I) (PDB ID: 2VU3) was downloaded from the PDB.^19^ The binding mode of the designed compounds was studied to explain their biological results and detect the essential hydrogen bonding with Leu83. The best pose out of ten for each compound was selected compared to the ligand binding mode. *In silico* ADMET studies using the Discovery Studio 4.1 Software and drug-likeness applying the Boiled-egg chart using https://www.swissadme.ch/index.php (ref. 37–45) were carried out to predict the pharmacokinetic properties of the targeted compounds, which helped in the structure requirement prediction of the observed anticancer activity.

## Data availability

The data supporting this article have been included as part of the ESI.†

## Conflicts of interest

The authors declare no conflict of interest.

## Supplementary Material

RA-014-D4RA06500J-s001
