# Supplementary material for: Discovery of pyrazole-based analogs as CDK2 inhibitors with apoptotic-inducing activity: design, synthesis and molecular dynamics study
Source: RSC Adv. 2024 Oct 29;14(47):34537–55. doi: 10.1039/d4ra06500j (PMC11520566; doi:10.1039/d4ra06500j)

This journal is © The Royal Society of Chemistry 2024

Supplementary:

Supplementary material index:

S1: Table of relative activity of the synthesized compounds
S2: Dose response curve of compound **4** at five dose assay against all cell lines at NCI
S3: Summary of five doses results of compound **4** from NCI
S4: Total energy versus time (production step) for the free protein
S5: Total energy versus time (production step) for compounds **AT7519 (I)**, **4**, **7c** and **9**
S6: RMSD free protein
S7: RMSD compounds **AT7519 (I)**, **4**, **7c** and **9**
S8: RMSF free protein
S9: RMSF compounds **AT7519 (I)**, **4**, **7c** and **9**
S10: ^{1}H-NMR of compound **2a**
S11: ^{13}C-NMR of compound **2a**
S12: ^{1}H-NMR of compound **2b**
S13: ^{13}C-NMR of compound **2b**
S14: ^{1}H-NMR of compound **2c**
S15: ^{13}C-NMR of compound **2c**
S16: ^{1}H-NMR of compound **2d**
S17: ^{13}C-NMR of compound **2d**
S18: ^{1}H-NMR of compound **2e**
S19: ^{13}C-NMR of compound **2e**
S20: ^{1}H-NMR of compound **2f**
S21: ^{13}C-NMR of compound **2f**
S22: ^{1}H-NMR of compound **2g**
S23: ^{13}C-NMR of compound **2g**
S24: ^{1}H-NMR of compound **4**
S25: ^{13}C-NMR of compound **4**
S26: ^{1}H-NMR of compound **7a**
S27: ^{13}C-NMR of compound **7a**
S28: ^{1}H-NMR of compound **7b**
S29: ^{13}C-NMR of compound **7b**
S30: ^{1}H-NMR of compound **7c**
S31: ^{13}C-NMR of compound **7c**
S32: ^{1}H-NMR of compound **7d**
S33: ^{13}C-NMR of compound **7d**
S34: ^{1}H-NMR of compound **8a**
S35: ^{13}C-NMR of compound **8a**
S36: ^{1}H-NMR of compound **8b**
S37: ^{1}H-NMR of compound **9**
S38: ^{13}C-NMR of compound **9**
S39: ^{1}H-NMR of compound **10**
S40: ^{13}C-NMR of compound **10**

S41: IR of compound **2a**
S42: IR of compound **2b**
S43: IR of compound **2e**
S44: IR of compound **2f**
S45: IR of compound **2g**
S46: Inhibition of CDK2/CyclinA2 by compounds **2a-g**, **4**, **7a-d**, **8a-b**, **9** &**10** at 50 μM.
S47: The dose–response curves show the Inhibitory Concentration at 50% (IC_{50}) curves of compounds **4**, **7c**, **7d**, **8b and 9** in CDK2/Cyclin A2 protein kinase activity assay.
S48: Flow cytometric analysis for cell cycle distribution. (A) Control HCT-116, (B) Compound **4**, and (C) graphical representation for cell cycle distribution analysis among differently treated cells.
S49: Flow cytometric analysis of apoptosis among treated cells. (A) Control HCT-116, (B) Compound **4**, (C) Graphical illustration of apoptosis % among differently treated cells.
S50: Cell cycle and apoptosis raw data
S51: Western Blot assay for compounds **4**, **9**
S52: Mass Results compound **4**
S53: Mass Results compound **7d**
S54: Mass results compound **8a**
S55: Mass Results compound **9**
S56: Mass Results compound **10**

S1Table of relative activity of the synthesized compounds

Sample no.	Relative activity
2a	51.4
2b	55.6
2c	59.3
2d	40.2
2e	56.6
2f	62.1
2g	59.5
4	24.7
7a	42.3
7b	70.7
7c	68.6
7d	11.5
8a	56.4
8b	27.1
9	14.5
10	49.9
DMSO	100

S2: Dose response curve of compound **4** at five dose assay against all cell lines at NCI

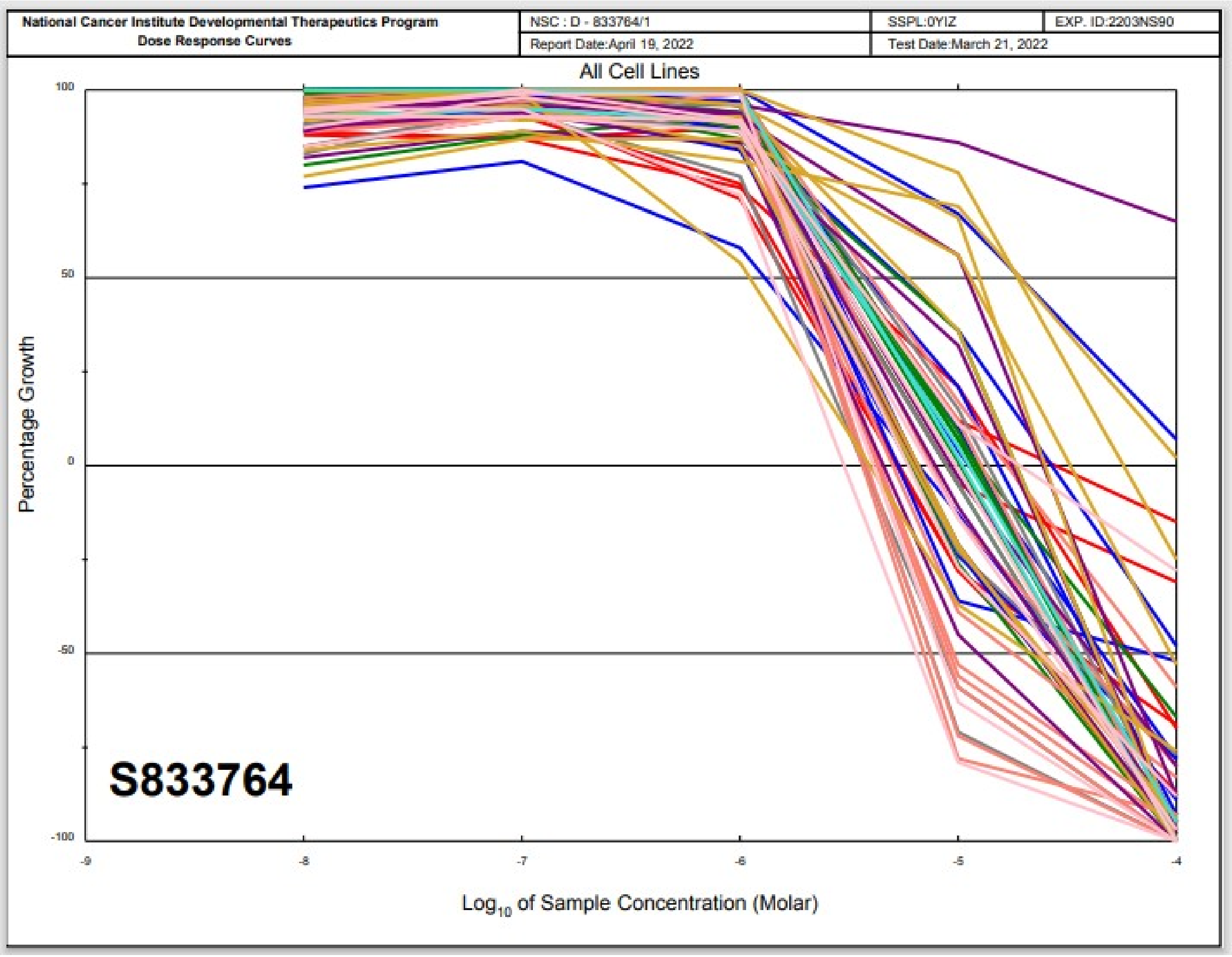

S3: Summary of five doses results of compound **4** from NCI

National Cancer Institute Developmental Therapeutics Program
In-Vitro Testing Results

NSC : D - 833764 / 1	Experiment ID : 2203NS90	Test Type : 08	Units : Molar
Report Date : April 19, 2022	Test Date : March 21, 2022	QNS :	MC :
COMI : h	Stain Reagent : SRB Dual-Pass Related	SSPL 0YIZ	

	Log10 Concentration														
	Time		Mean Optical Densities					Percent Growth							
Panel/Cell Line	Zero	Ctrl	-8.0	-7.0	-6.0	-5.0	-4.0	-8.0	-7.0	-6.0	-5.0	-4.0	GI50	TGI	LC50
Leukemia															
CCRF-CEM	1.043	3.064	3.018	3.061	2.854	0.994	0.716	98	100	90	-5	-31	2.63E-6	8.92E-6	> 1.00E-4
HL-60(TB)	0.485	1.427	1.326	1.309	1.185	0.684	0.146	89	87	74	21	-70	2.86E-6	1.71E-5	6.05E-5
K-562	0.270	1.741	1.571	1.548	1.599	0.211	-0.068	88	87	90	-22	-100	2.29E-6	6.37E-6	2.28E-5
MOLT-4	0.690	2.430	2.352	2.350	1.928	0.499	0.216	96	95	71	-28	-69	1.64E-6	5.24E-6	3.49E-5
RPMI-8226	0.822	2.690	2.705	2.714	2.537	1.053	0.698	101	101	92	12	-15	3.36E-6	2.82E-5	> 1.00E-4
SR	0.440	1.625	1.444	1.545	1.326	0.317	0.058	85	93	75	-28	-87	1.74E-6	5.33E-6	2.36E-5
Non-Small Cell Lung Cancer															
A549/ATCC	0.329	1.692	1.690	1.806	1.690	1.247	0.430	100	108	100	67	7	1.94E-5	> 1.00E-4	> 1.00E-4
EKVX	0.814	1.567	1.555	1.580	1.483	1.084	0.427	98	102	89	36	-48	5.39E-6	2.69E-5	> 1.00E-4
HOP-62	0.727	2.254	2.115	2.171	2.003	0.554	0.159	91	95	84	-24	-78	2.05E-6	6.00E-6	3.03E-5
HOP-92	1.287	1.716	1.603	1.632	1.537	1.118	0.143	74	81	58	-13	-89	1.30E-6	6.55E-6	3.07E-5
NCI-H226	0.741	2.104	2.044	2.135	2.013	0.878	-0.042	96	102	93	10	-100	3.31E-6	1.23E-5	3.51E-5
NCI-H23	0.509	1.650	1.702	1.708	1.644	0.394	0.011	105	105	99	-23	-98	2.54E-6	6.53E-6	2.31E-5
NCI-H322M	0.962	2.473	2.511	2.462	2.308	1.273	0.069	103	99	89	21	-93	3.72E-6	1.52E-5	4.19E-5
NCI-H460	0.248	2.503	2.543	2.568	2.445	0.334	0.054	102	103	97	4	-78	3.21E-6	1.11E-5	4.53E-5
NCI-H522	0.913	2.572	2.393	2.522	2.466	0.582	0.439	89	97	94	-36	-52	2.17E-6	5.25E-6	7.48E-5
Colon Cancer															
COLO 205	0.481	1.784	1.801	1.851	1.708	0.592	-0.049	101	105	94	9	-100	3.28E-6	1.20E-5	3.46E-5
HCC-2998	0.450	1.760	1.499	1.606	1.662	0.335	-0.232	80	88	93	-26	-100	2.29E-6	6.08E-6	2.13E-5
HCT-116	0.248	2.090	2.047	2.158	2.008	0.270	-0.019	98	104	96	1	-100	3.04E-6	1.03E-5	3.20E-5
HCT-15	0.159	0.999	0.993	0.979	0.888	0.461	-0.024	99	98	87	36	-100	5.29E-6	1.84E-5	4.29E-5
HT29	0.205	1.302	1.307	1.490	1.341	0.280	-0.071	100	117	104	7	-100	3.58E-6	1.16E-5	3.40E-5
KM12	0.483	2.454	2.409	2.477	2.261	0.461	-0.058	98	101	90	-5	-100	2.66E-6	8.95E-6	2.99E-5
SW-620	0.332	2.569	2.603	2.615	2.414	0.497	0.109	102	102	93	7	-67	3.18E-6	1.26E-5	5.88E-5
CNS Cancer															
SF-268	0.752	2.403	2.151	2.314	2.214	0.580	0.171	85	95	89	-23	-77	2.22E-6	6.23E-6	3.15E-5
SF-295	0.519	1.680	1.487	1.631	1.630	0.690	0.025	83	96	96	15	-95	3.67E-6	1.36E-5	3.87E-5
SF-539	0.682	2.085	1.925	2.008	1.764	0.278	-0.093	89	94	77	-59	-100	1.58E-6	3.68E-6	8.56E-6
SNB-19	0.825	2.472	2.329	2.369	2.293	0.780	-0.161	91	94	89	-5	-100	2.59E-6	8.76E-6	2.96E-5
U251	0.313	1.619	1.551	1.686	1.565	0.092	-0.072	95	105	96	-71	-100	1.88E-6	3.76E-6	7.51E-6
Melanoma															
LOX IMVI	0.298	2.411	2.369	2.446	2.479	0.123	-0.058	98	102	103	-59	-100	2.13E-6	4.33E-6	8.81E-6
MALME-3M	0.702	1.338	1.268	1.321	1.348	0.199	0.002	89	97	102	-72	-100	1.98E-6	3.86E-6	7.49E-6
M14	0.470	1.753	1.657	1.770	1.793	0.403	0.096	93	101	103	-14	-80	2.83E-6	7.55E-6	3.51E-5
MDA-MB-435	0.594	2.799	2.575	2.761	2.603	0.364	0.099	90	98	91	-39	-83	2.07E-6	5.03E-6	1.78E-5
SK-MEL-2	1.108	2.591	2.569	2.653	2.606	0.483	0.042	98	104	101	-56	-96	2.11E-6	4.38E-6	9.10E-6
SK-MEL-28	0.742	2.181	2.126	2.305	2.178	0.167	0.051	96	109	100	-78	-93	1.91E-6	3.65E-6	6.99E-6
SK-MEL-5	0.777	2.827	2.718	2.835	2.648	1.024	-0.162	95	100	91	12	-100	3.32E-6	1.28E-5	3.58E-5
UACC-257	1.043	2.587	2.423	2.486	2.574	1.306	0.425	89	93	99	17	-59	3.97E-6	1.67E-5	7.55E-5
UACC-62	0.696	2.421	2.238	2.293	2.290	0.330	0.051	89	93	92	-53	-93	1.96E-6	4.34E-6	9.60E-6
Ovarian Cancer															
IGROV1	0.550	2.438	2.257	2.327	2.149	1.146	0.018	90	94	85	32	-97	4.50E-6	1.76E-5	4.32E-5
OVCAR-3	0.365	1.636	1.410	1.491	1.452	0.202	-0.083	82	89	86	-45	-100	1.87E-6	4.54E-6	1.25E-5
OVCAR-4	0.612	1.772	1.699	1.753	1.704	0.592	0.123	94	98	94	-3	-80	2.84E-6	9.26E-6	4.07E-5
OVCAR-5	0.554	1.673	1.615	1.630	1.594	1.179	0.064	95	96	93	56	-88	1.10E-5	2.44E-5	5.42E-5
OVCAR-8	0.348	1.795	1.746	1.864	1.698	0.309	0.018	97	105	93	-11	-95	2.59E-6	7.81E-6	2.90E-5
NCI/ADR-RES	0.585	2.189	2.141	2.210	2.119	1.959	1.627	97	101	96	86	65	> 1.00E-4	> 1.00E-4	> 1.00E-4
SK-OV-3	0.924	1.958	1.847	1.979	1.872	0.727	0.022	89	102	92	-21	-98	2.34E-6	6.47E-6	2.37E-5
Renal Cancer															
786-0	0.653	2.376	2.239	2.305	2.126	0.511	0.041	92	96	85	-22	-94	2.14E-6	6.27E-6	2.47E-5
A498	1.761	2.578	2.391	2.475	2.475	2.218	0.836	77	87	87	56	-53	1.14E-5	3.28E-5	9.47E-5
ACHN	0.437	1.705	1.626	1.751	1.653	0.898	-0.127	94	104	96	36	-100	5.90E-6	1.85E-5	4.30E-5
CAKI-1	0.751	2.386	2.329	2.426	2.314	1.837	-0.026	96	102	96	66	-100	1.25E-5	2.51E-5	5.01E-5
RXF 393	1.039	1.583	1.598	1.580	1.331	0.650	0.247	103	99	54	-37	-76	1.10E-6	3.88E-6	2.11E-5
SN12C	0.440	1.727	1.658	1.630	1.631	0.350	-0.140	95	92	93	-21	-100	2.38E-6	6.58E-6	2.35E-5
TK-10	1.040	1.969	1.943	1.986	1.975	1.761	0.777	97	102	101	78	-25	1.86E-5	5.68E-5	> 1.00E-4
UO-31	0.717	2.277	2.028	2.103	1.986	1.800	0.747	84	89	81	69	2	1.94E-5	> 1.00E-4	> 1.00E-4
Prostate Cancer															
PC-3	0.592	2.296	2.178	2.220	2.162	0.684	0.033	93	95	92	5	-95	3.06E-6	1.13E-5	3.58E-5
DU-145	0.401	1.793	1.799	1.872	1.785	0.425	-0.215	100	106	99	2	-100	3.21E-6	1.04E-5	3.22E-5
Breast Cancer															
MCF7	0.656	2.780	2.634	2.639	2.547	0.566	0.022	93	93	89	-14	-97	2.40E-6	7.34E-6	2.74E-5
MDA-MB-231/ATCC	0.580	1.470	1.423	1.464	1.458	0.213	-0.198	95	99	99	-63	-100	2.00E-6	4.07E-6	8.28E-6
HS 578T	1.456	2.946	2.724	2.849	2.827	1.622	1.053	85	93	92	11	-28	3.31E-6	1.94E-5	> 1.00E-4
BT-549	1.070	1.879	1.829	1.921	1.818	1.055	-0.206	94	105	92	-1	-100	2.83E-6	9.66E-6	3.11E-5
T-47D	0.806	1.883	1.772	1.864	1.781	0.591	0.094	90	98	91	-27	-88	2.22E-6	5.92E-6	2.39E-5

S4: Total energy versus time (production step) for the free protein

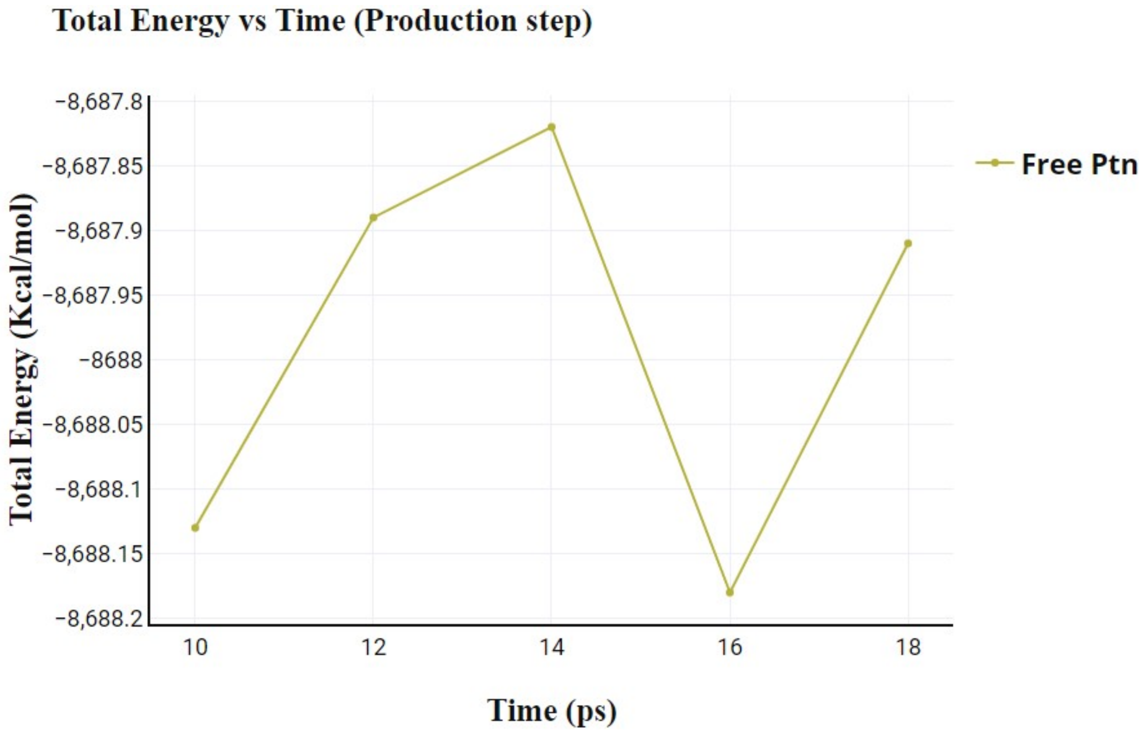

S5: Total energy versus time (production step) for compounds **AT7519 (I)**, **4**, **7a**, and **9**

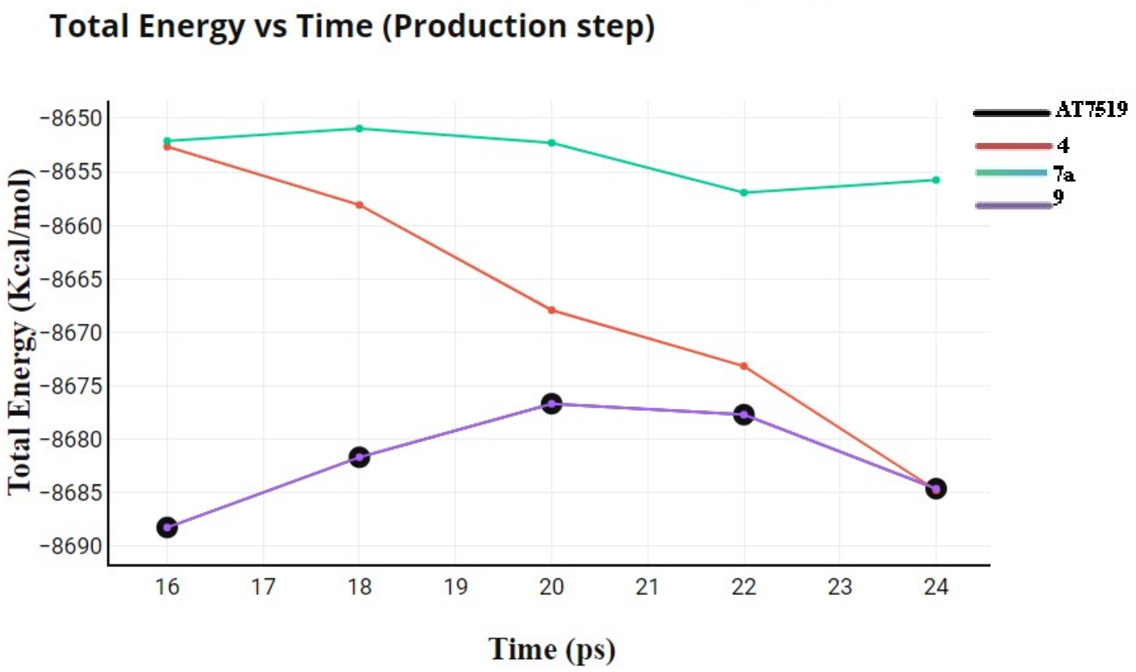

S6: RMSD free protein

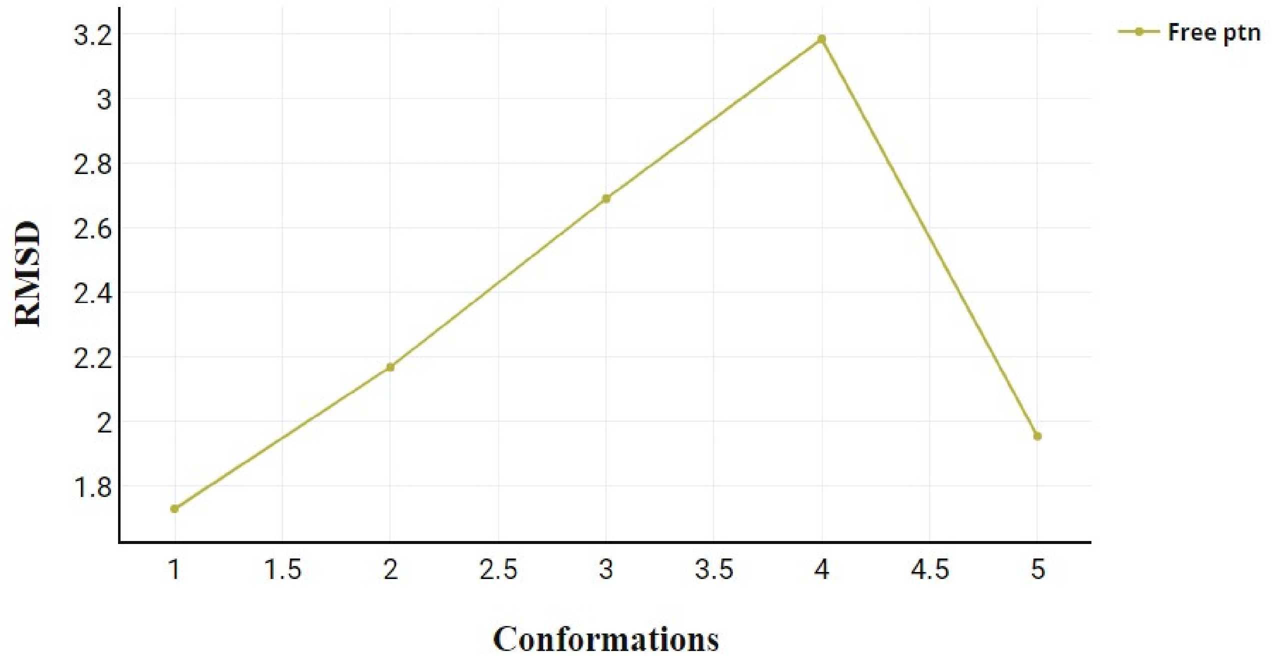

S7: RMSD compounds **AT7519 (I)**, **4**, **7a** and **9**

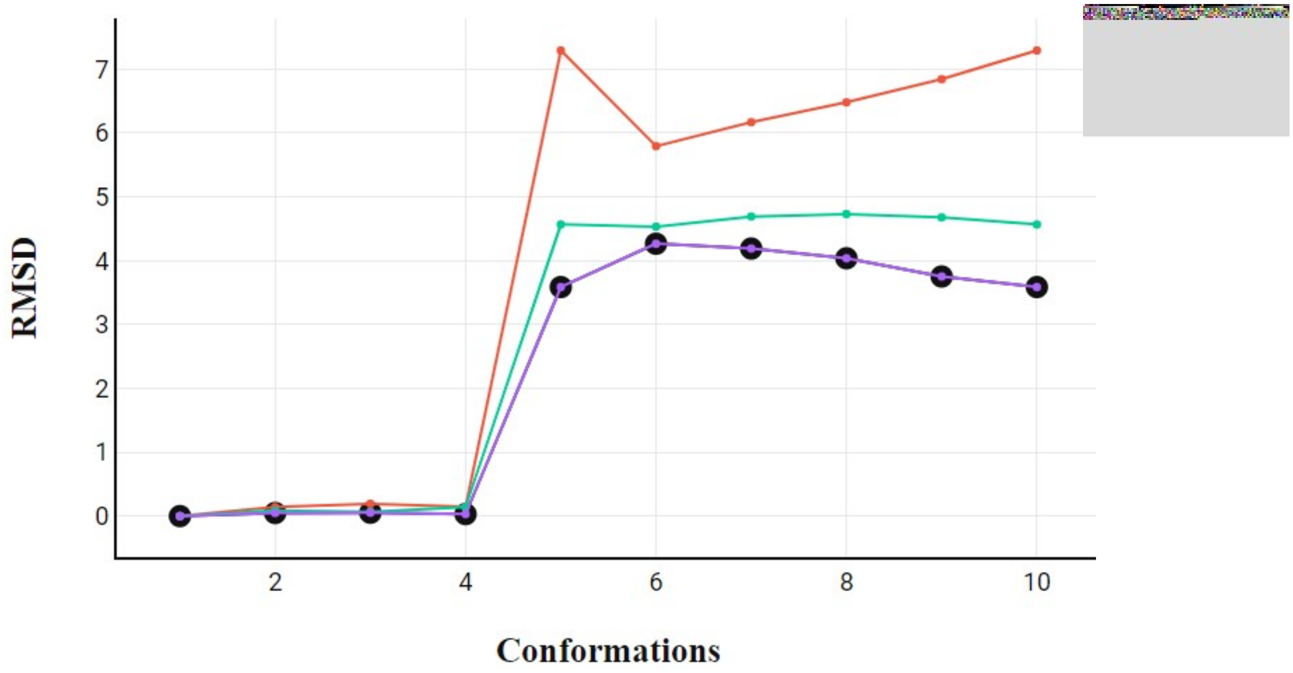

S8: RMSF free protein

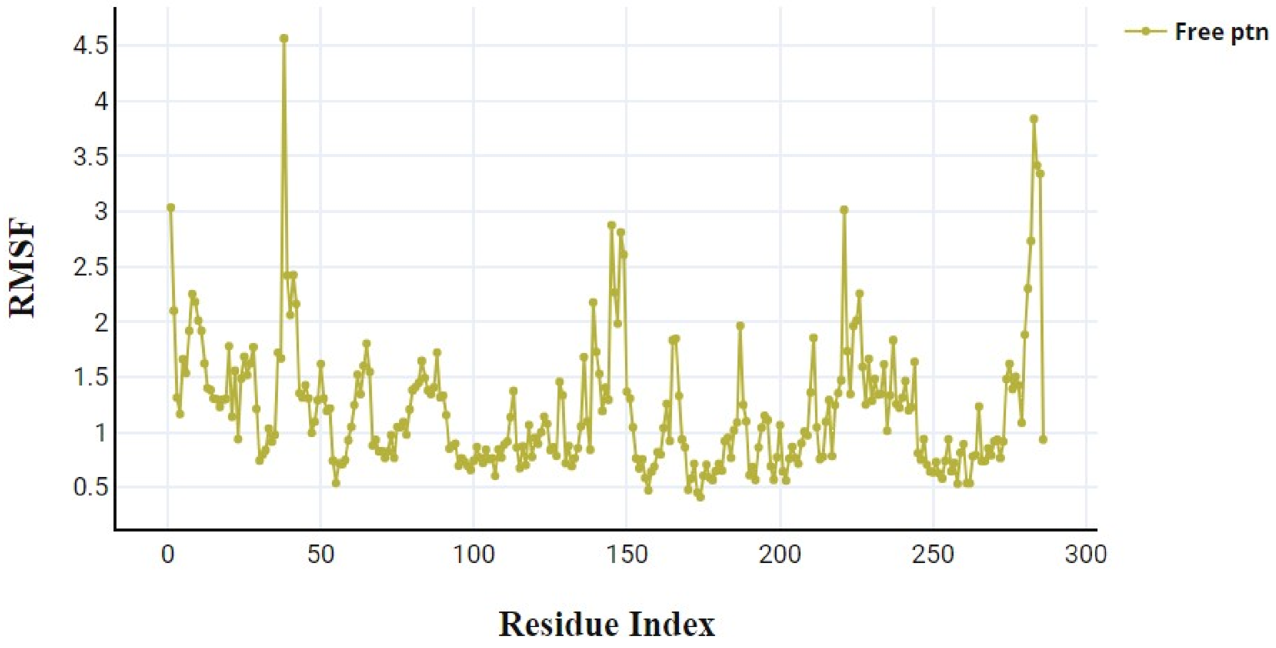

S9: RMSF compounds **AT7519 (I)**, **4**, **7a** and **9**

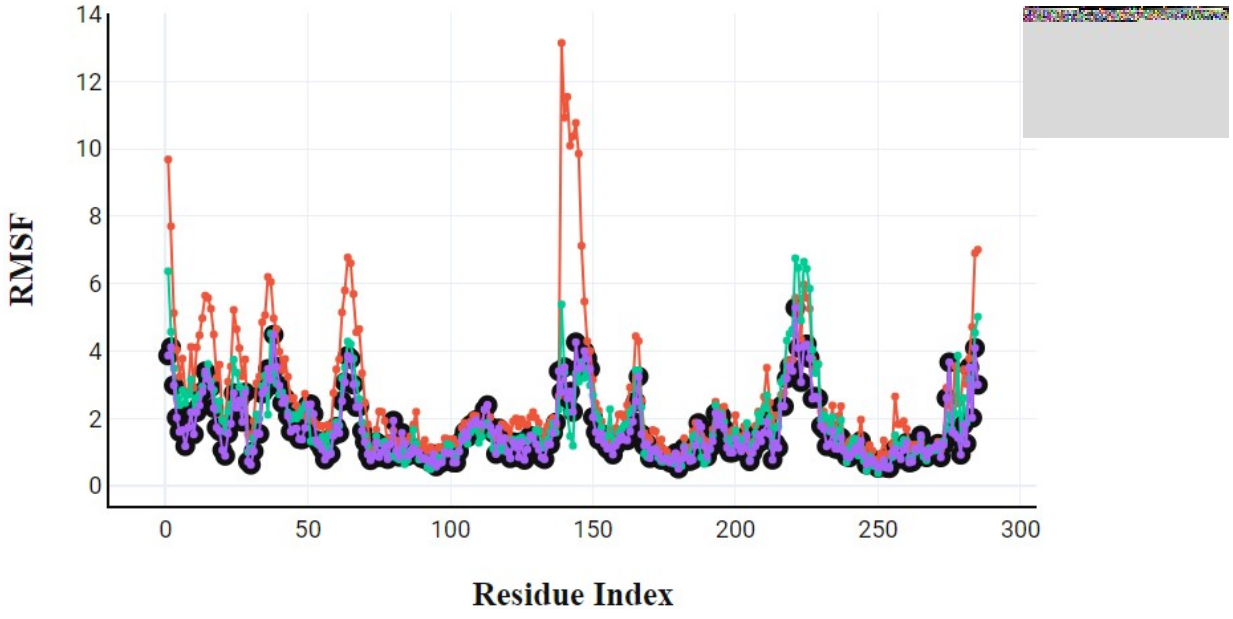

S10: ^{1}H-NMR of compound **2a**

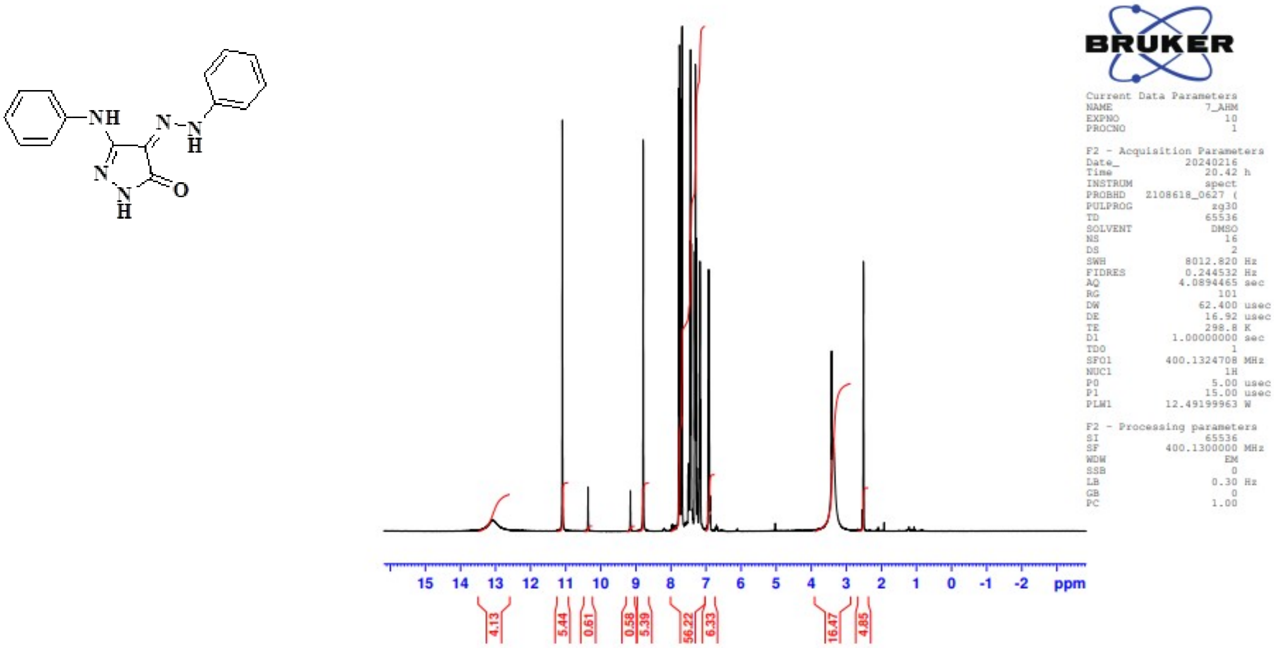

S11: ^{13}C-NMR of compound **2a**

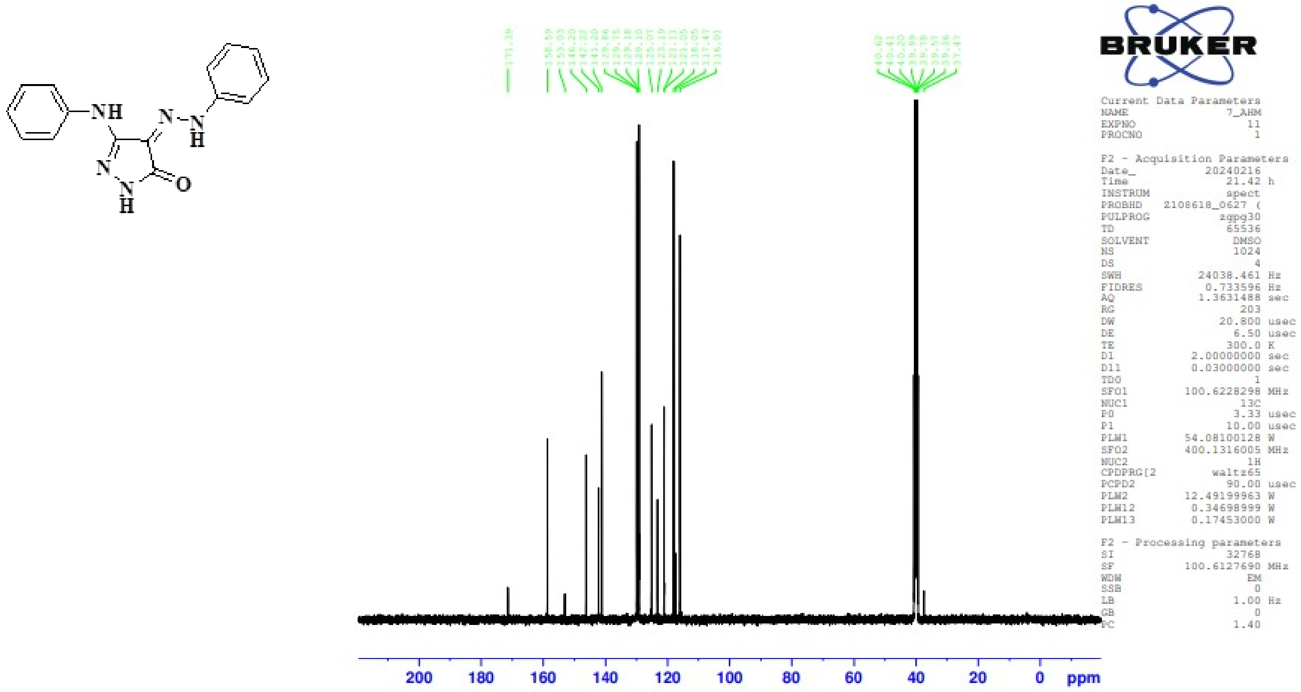

S12: ^{1}H-NMR of compound **2b**

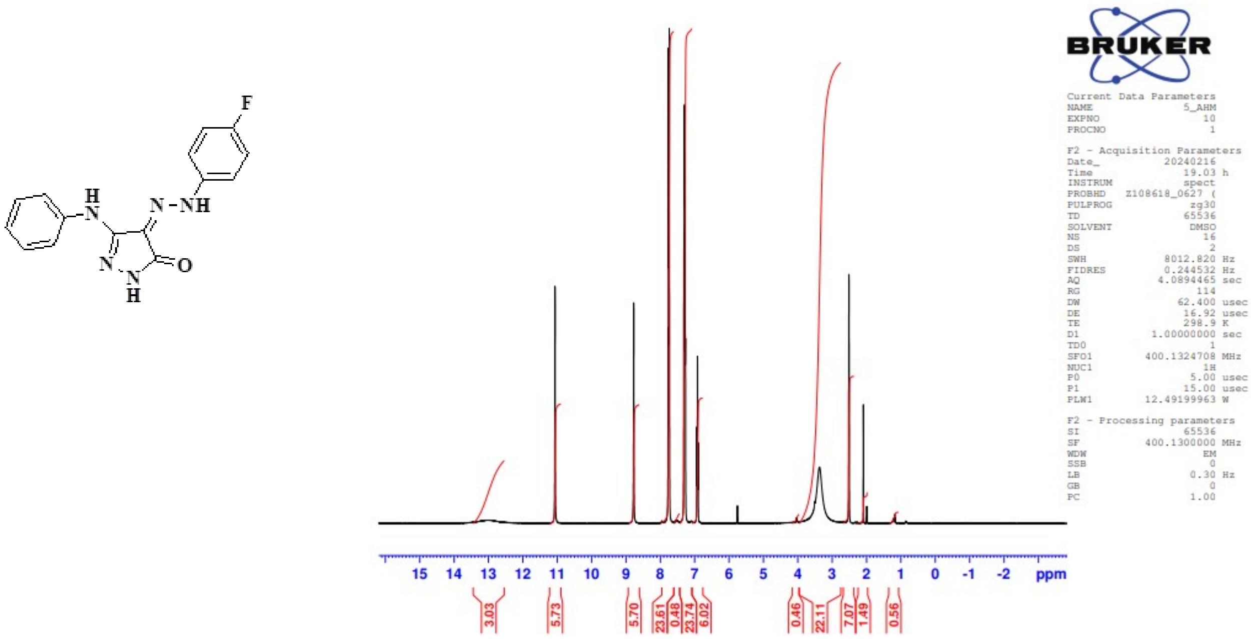

S13: ^{13}C-NMR of compound **2b**

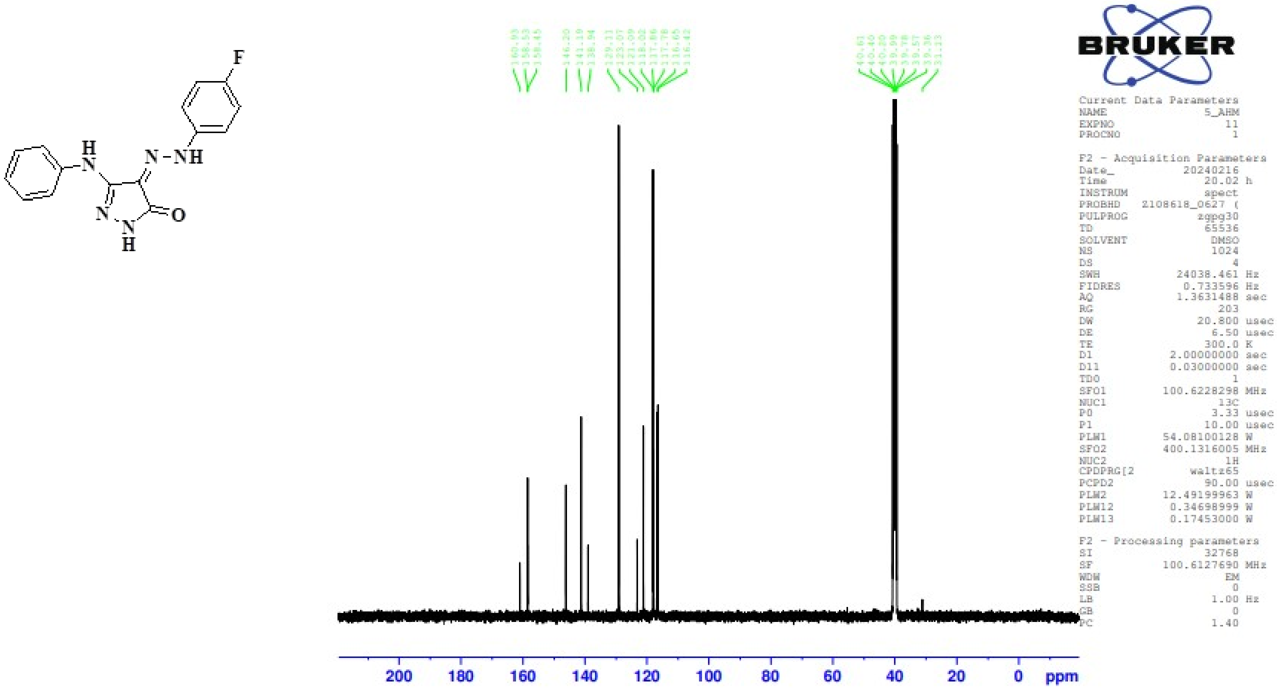

S14: ^{1}H-NMR of compound **2c**

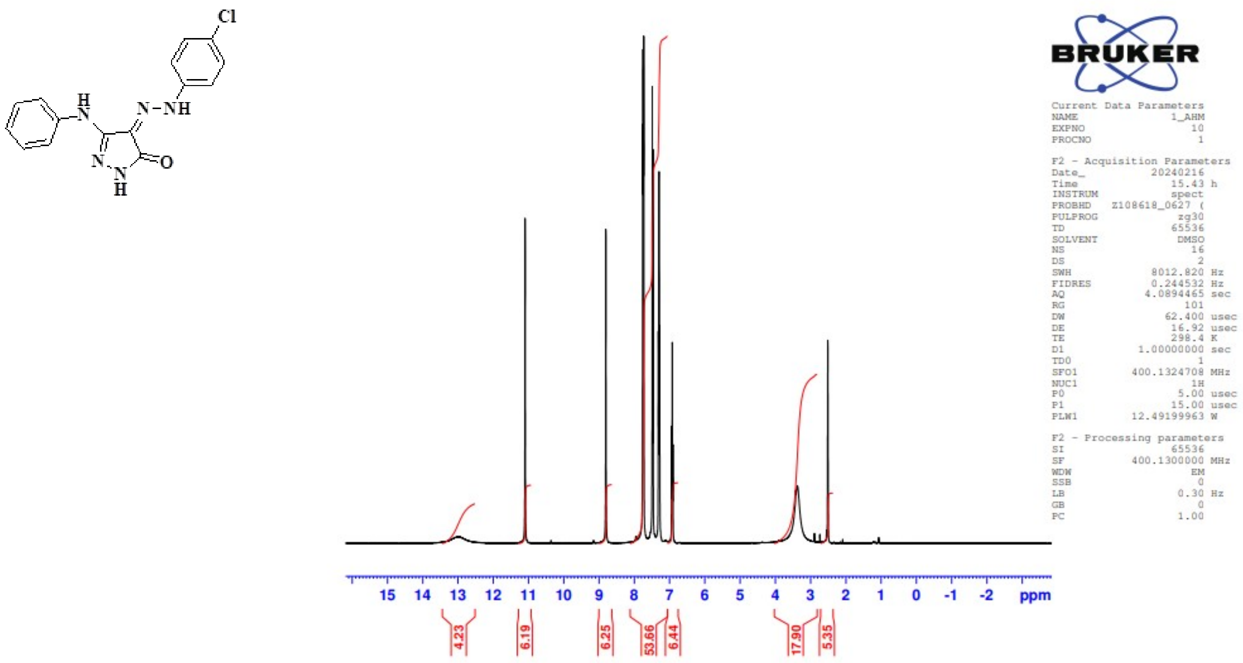

S15: ^{13}C-NMR of compound **2c**

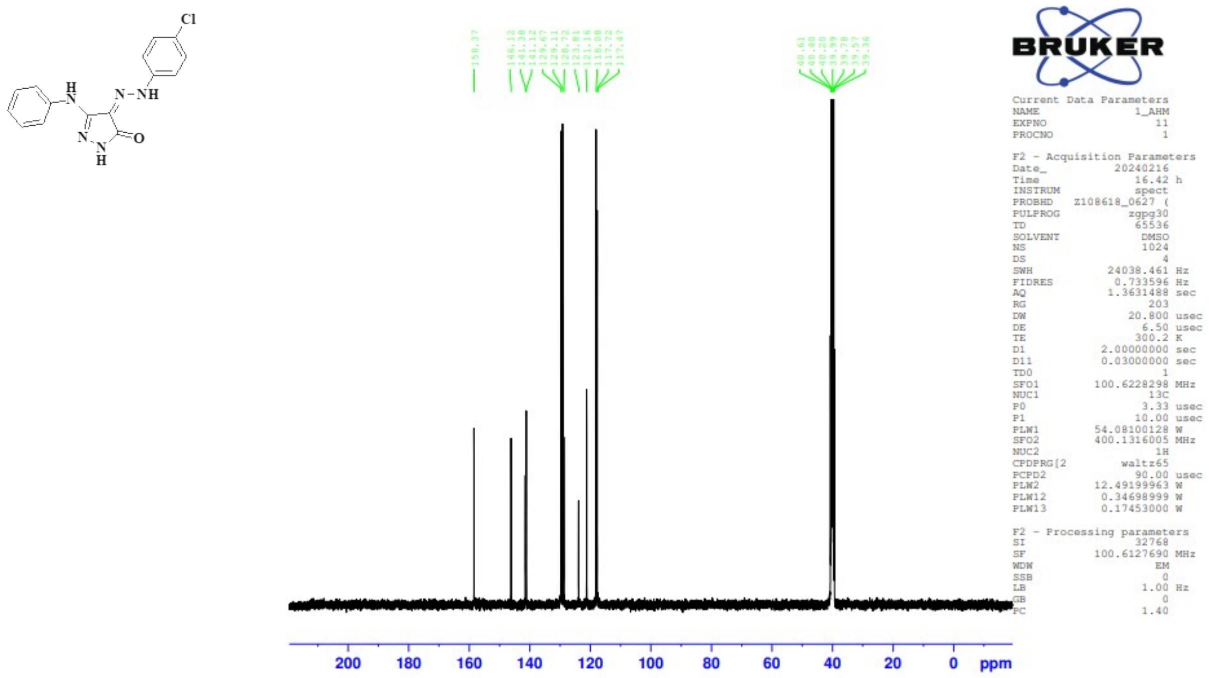

S16: ^{1}H-NMR of compound **2d**

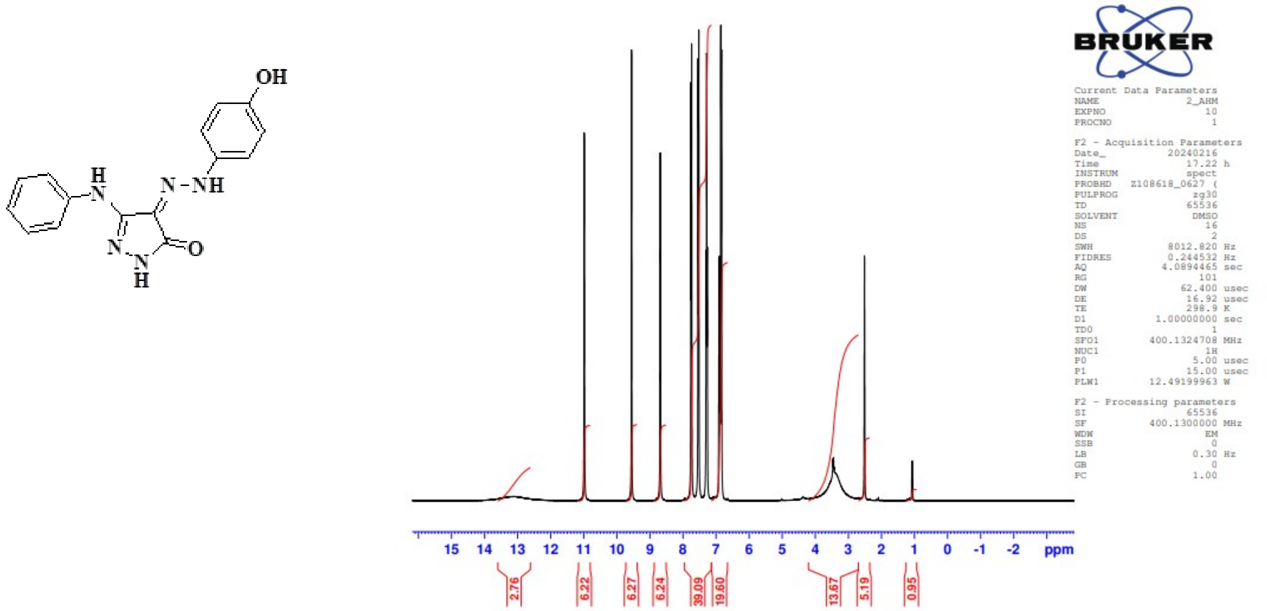

S17: ^{13}C-NMR of compound **2d**

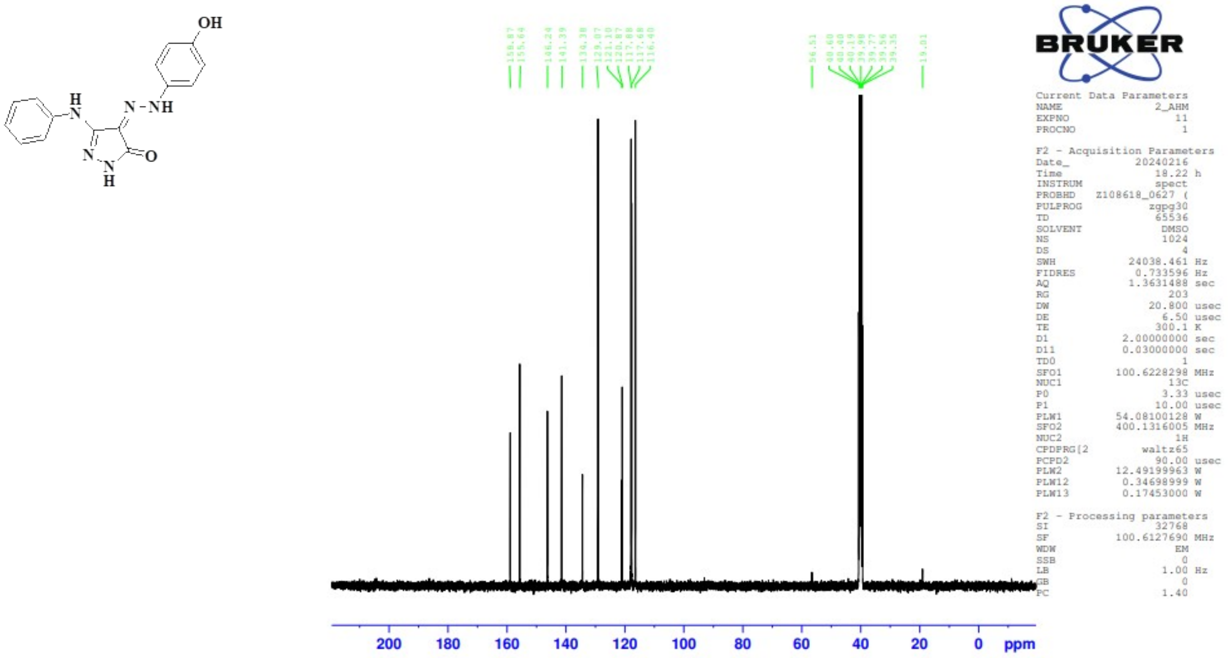

S18: ^{1}H-NMR of compound **2e**

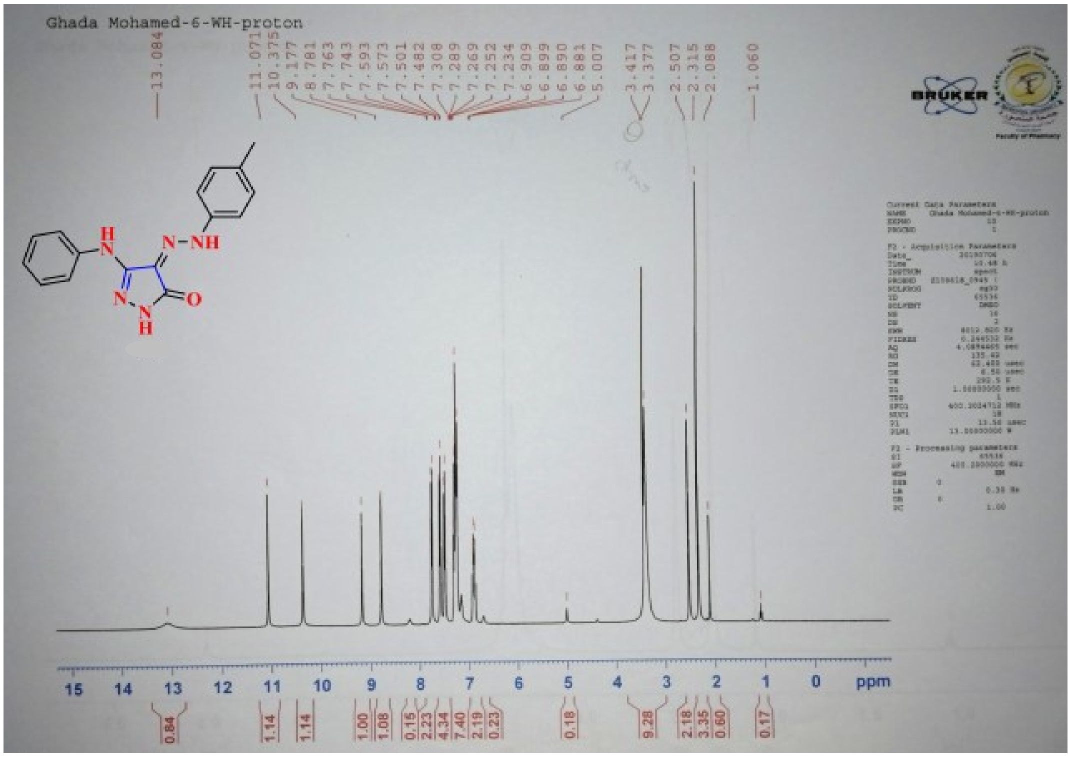

S19: ^{13}C-NMR of compound **2e**

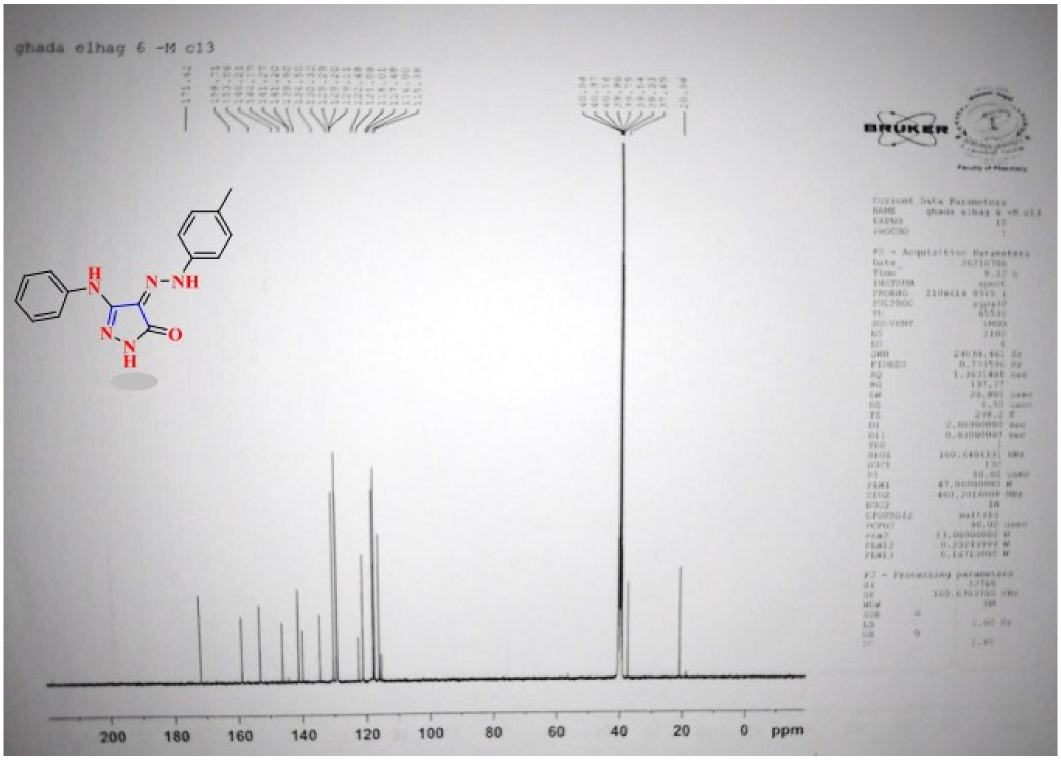

S20: ^{1}H-NMR of compound **2f**

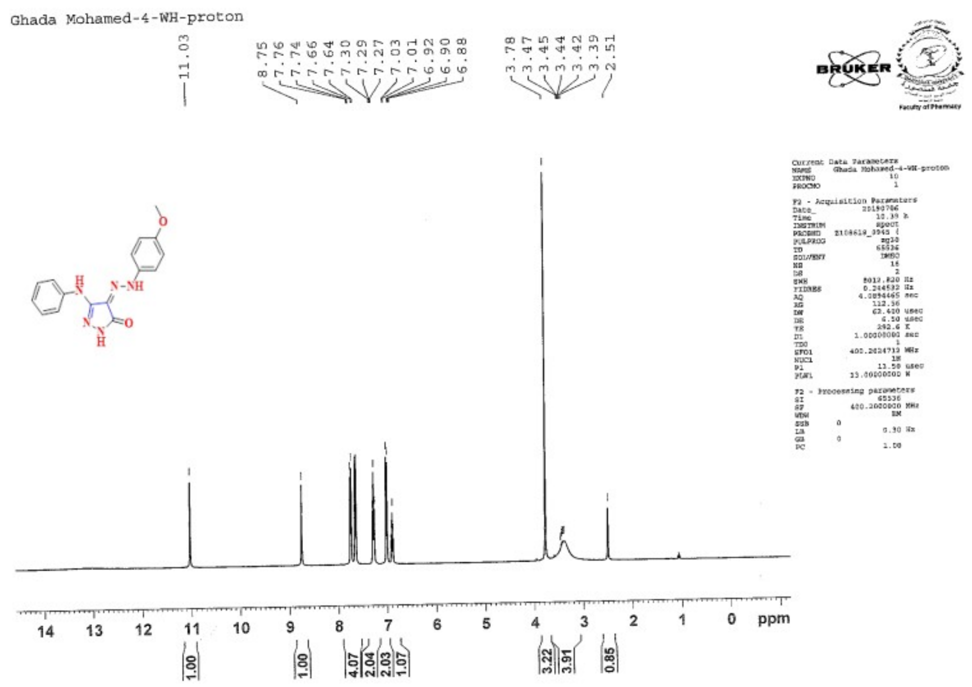

S21: ^{13}C-NMR of compound **2f**

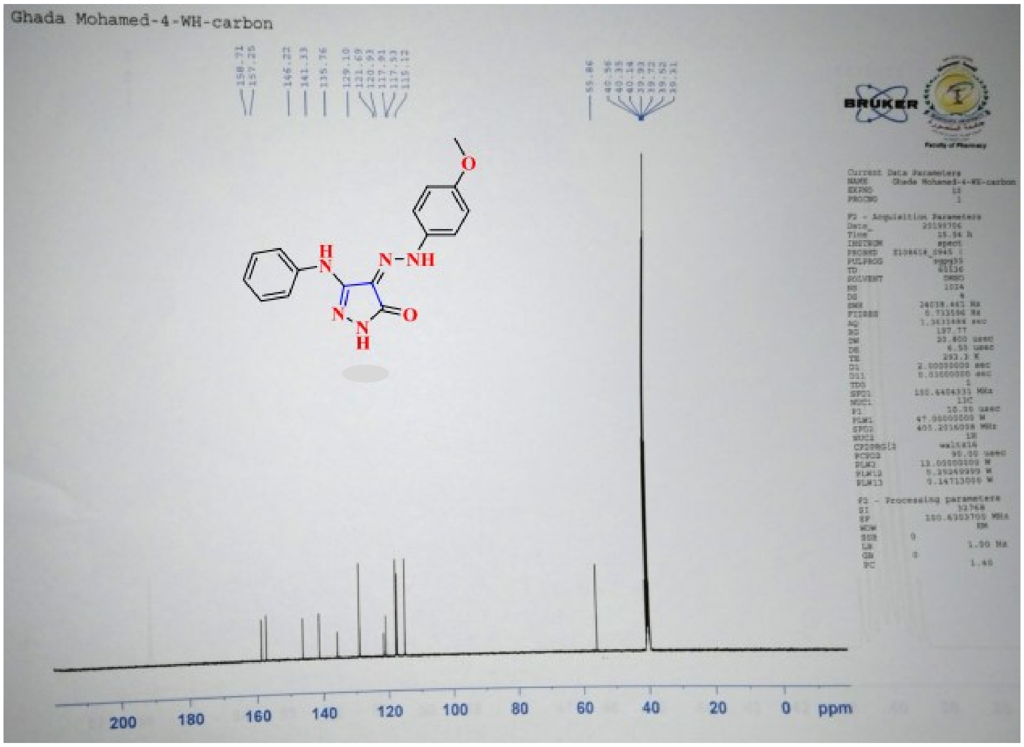

S22: ^{1}H-NMR of compound **2g**

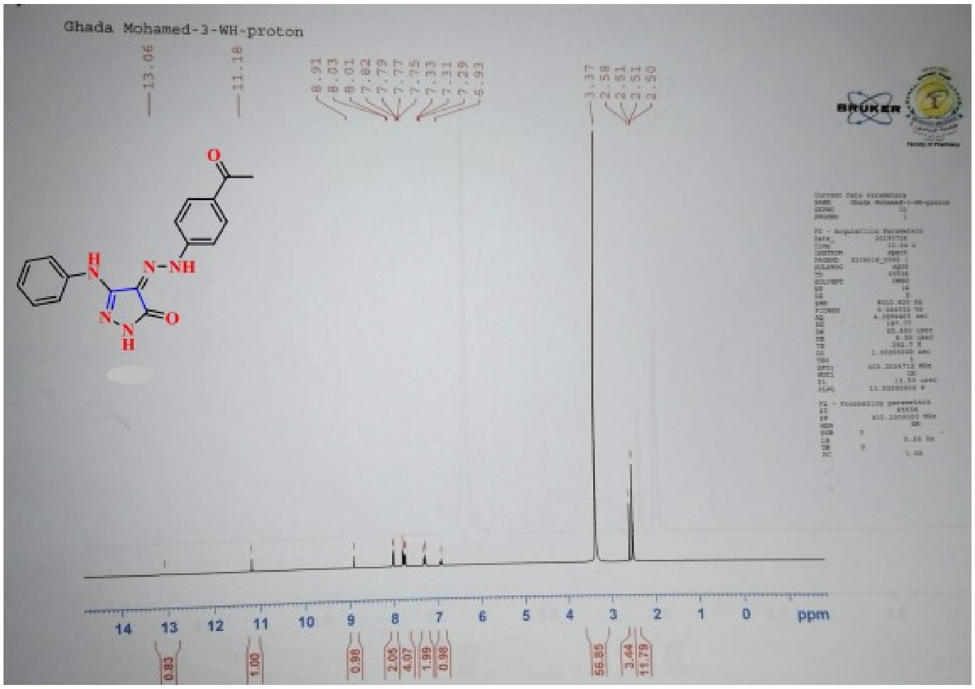

S23: ^{13}C-NMR of compound **2g**

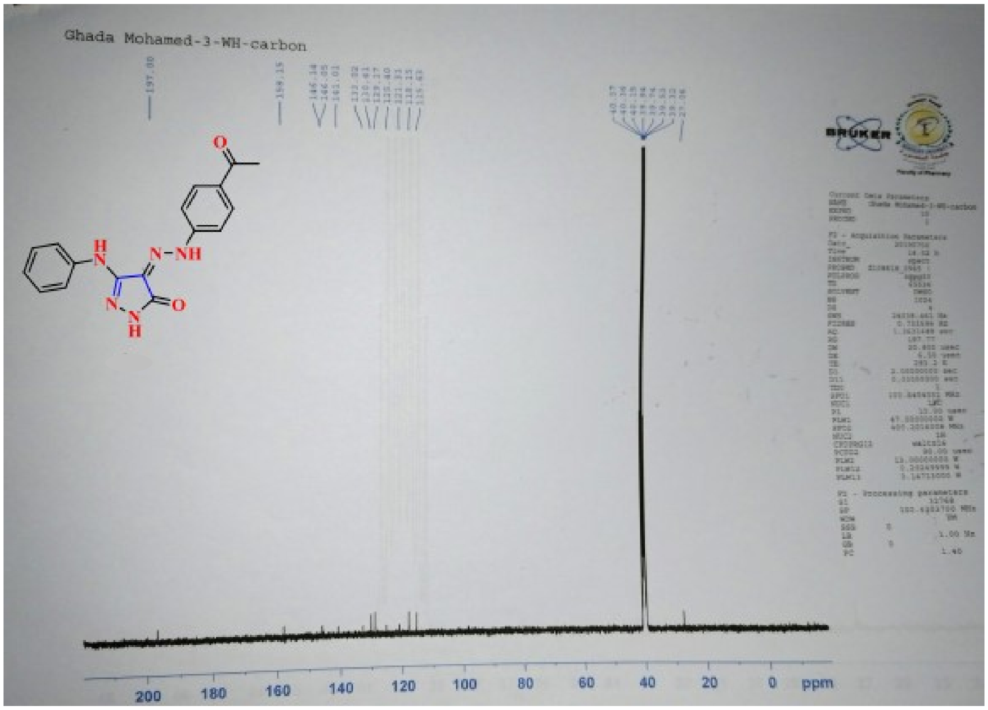

S24: ^{1}H-NMR of compound **4**

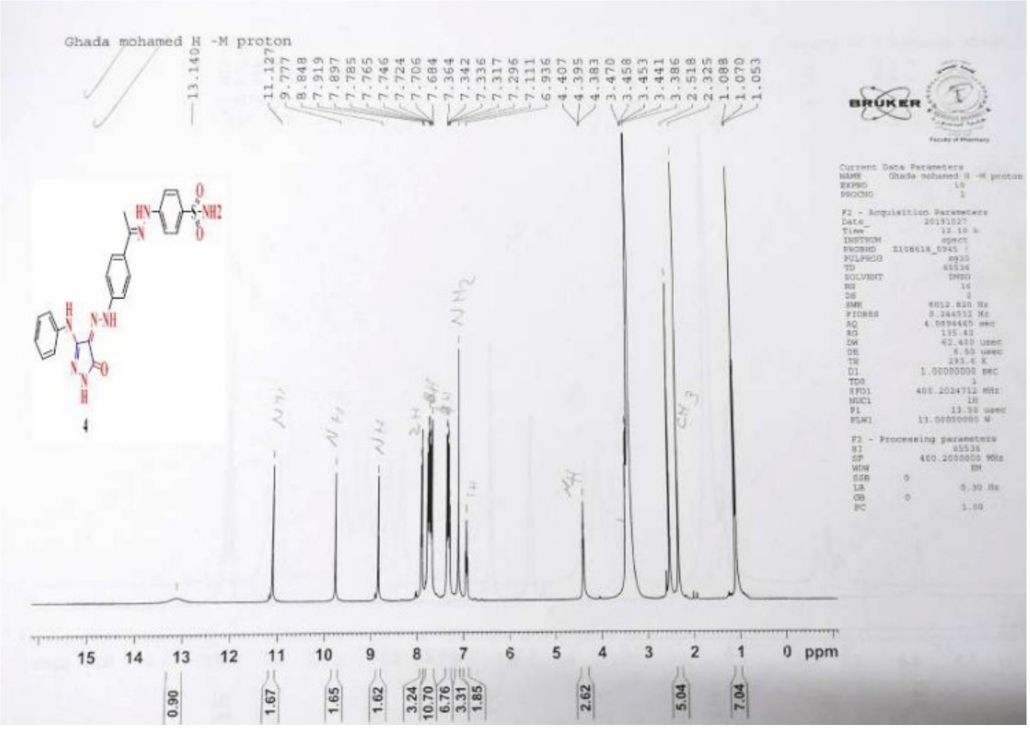

S25: ^{13}C-NMR of compound **4**

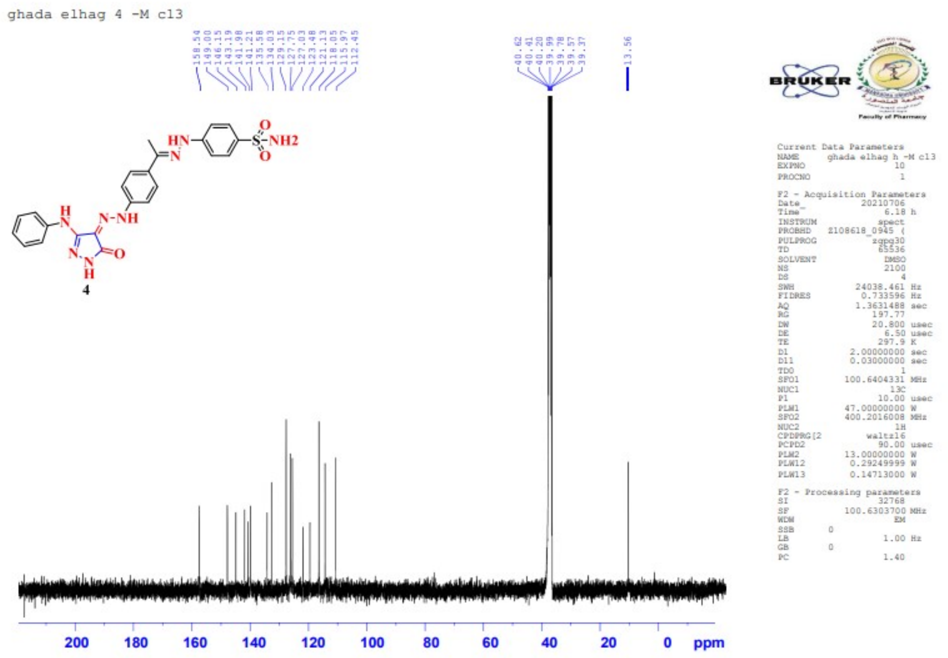

S26: ^{1}H-NMR of compound **7a**

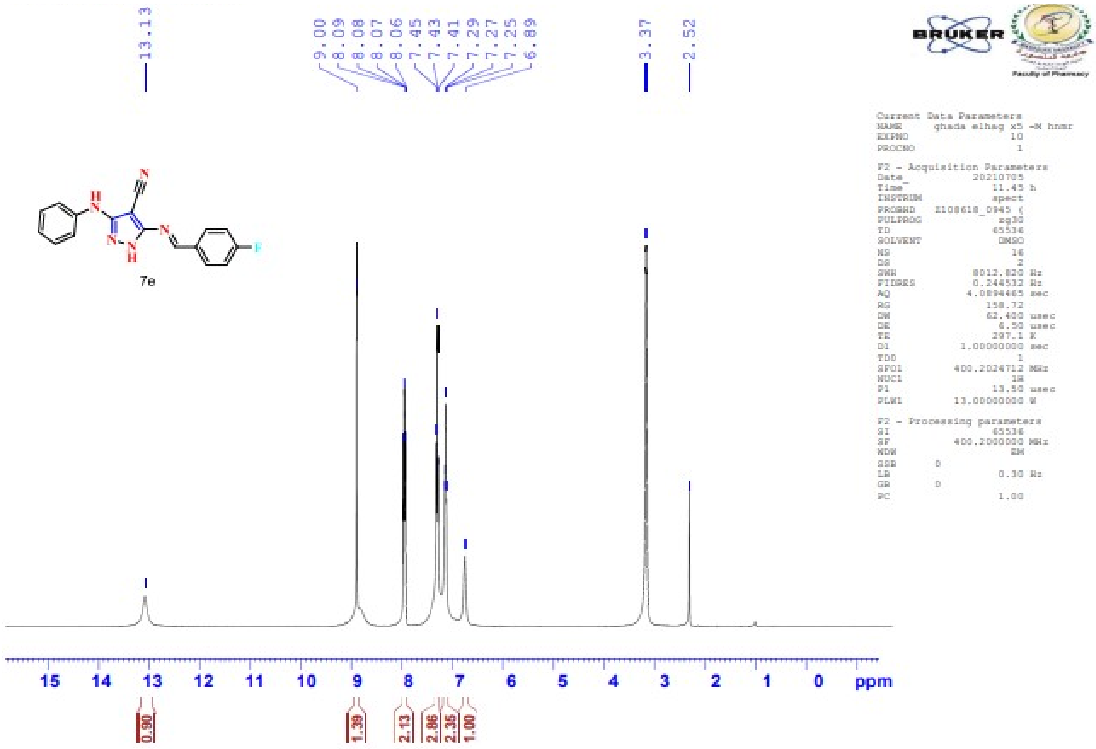

S27: ^{13}C-NMR of compound **7a**

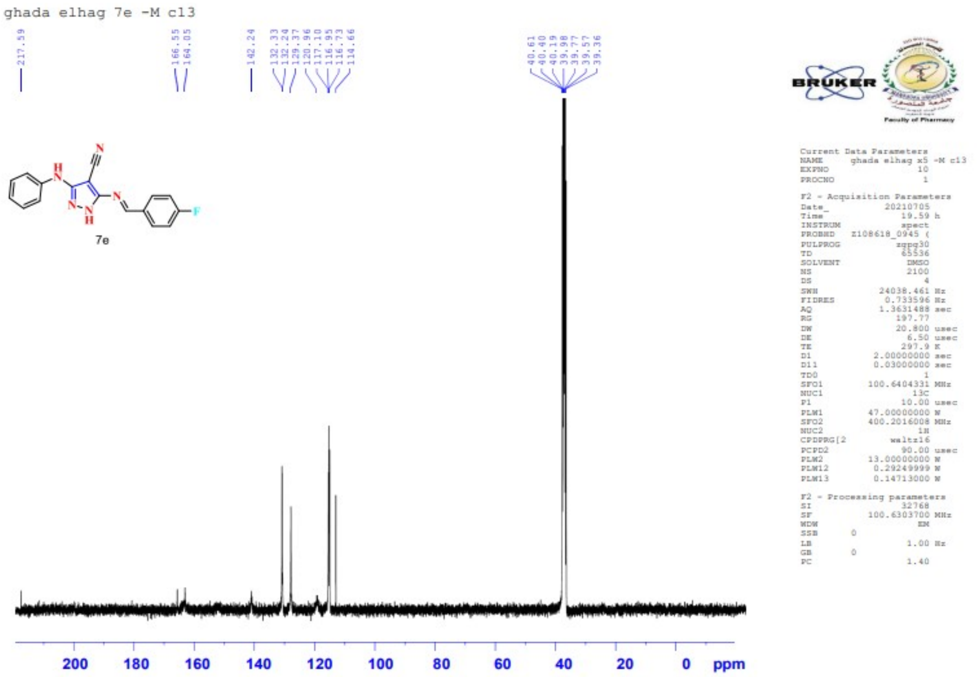

S28: ^{1}H-NMR of compound **7b**

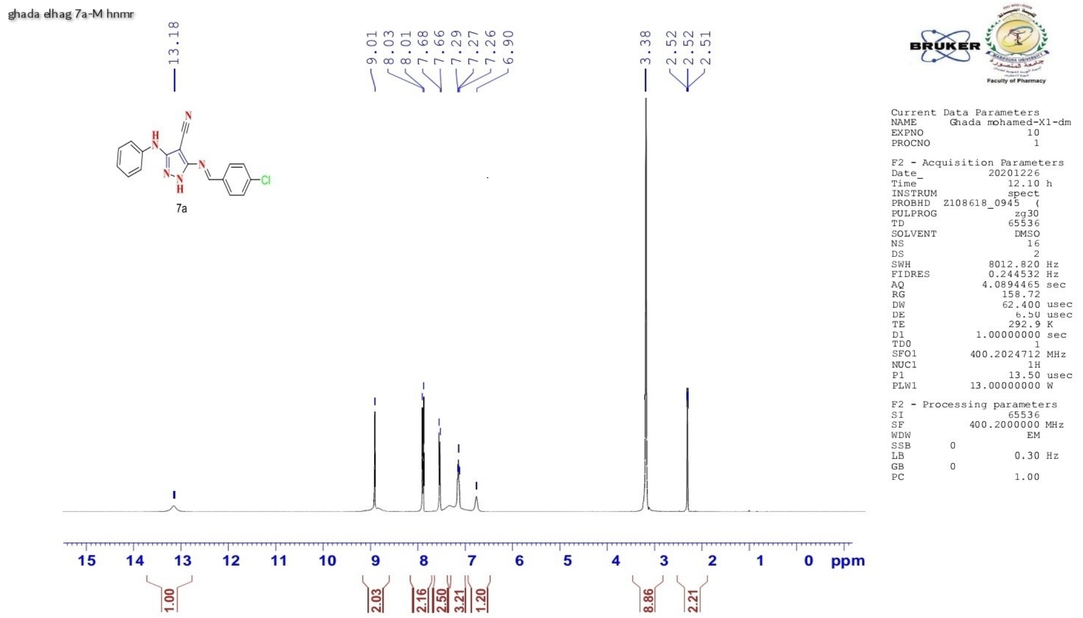

S29: ^{13}C-NMR of compound **7b**

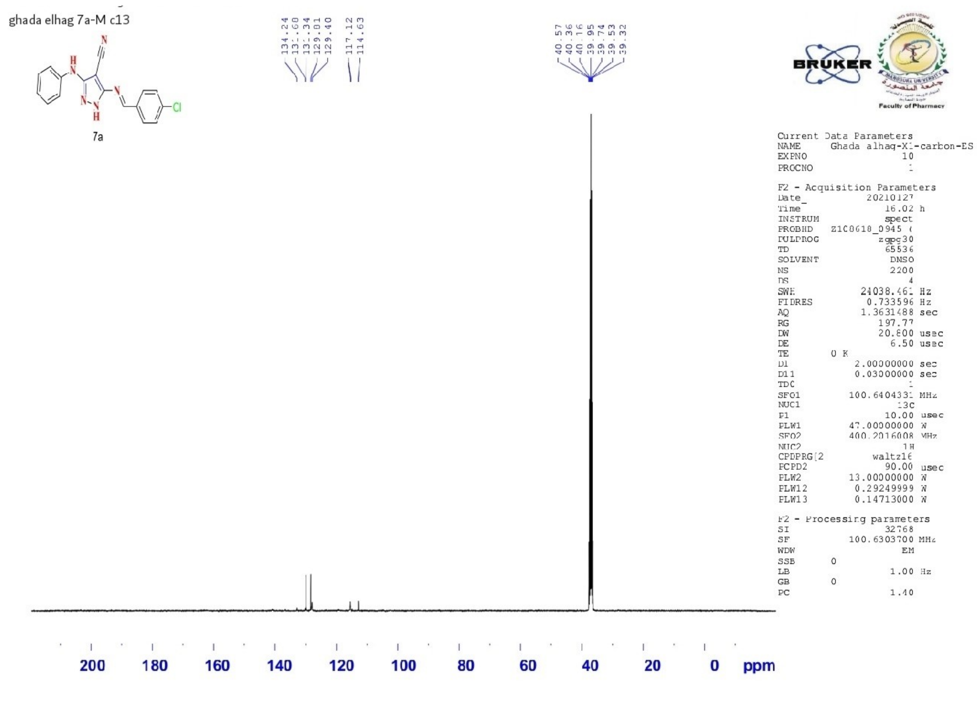

S30: ^{1}H-NMR of compound **7c**

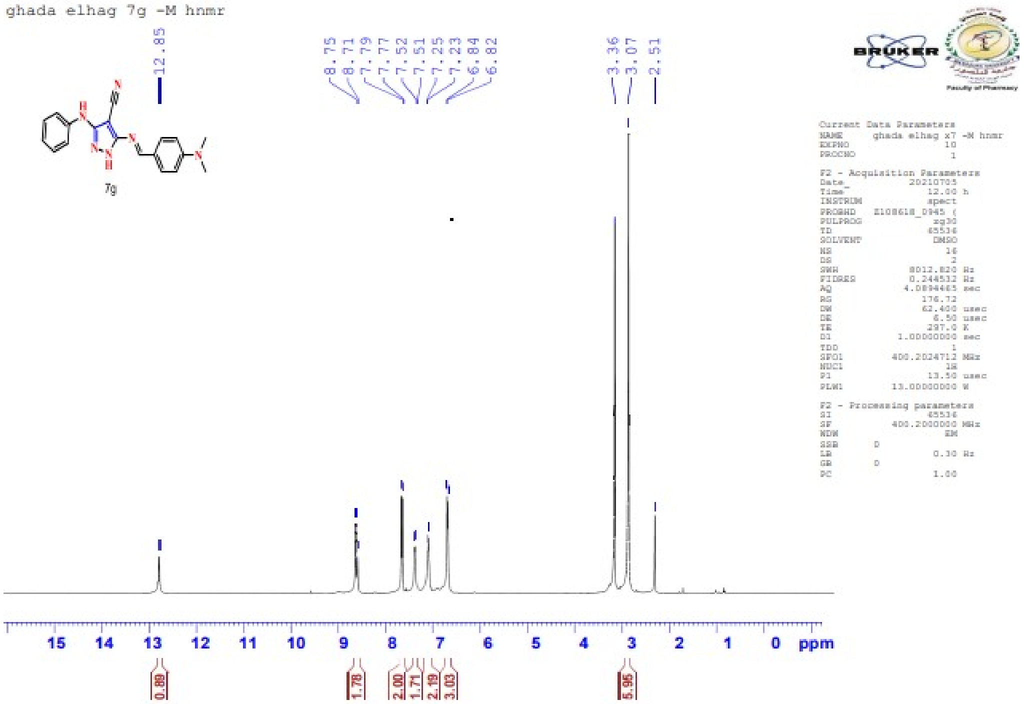

S31: ^{13}C-NMR of compound **7c**

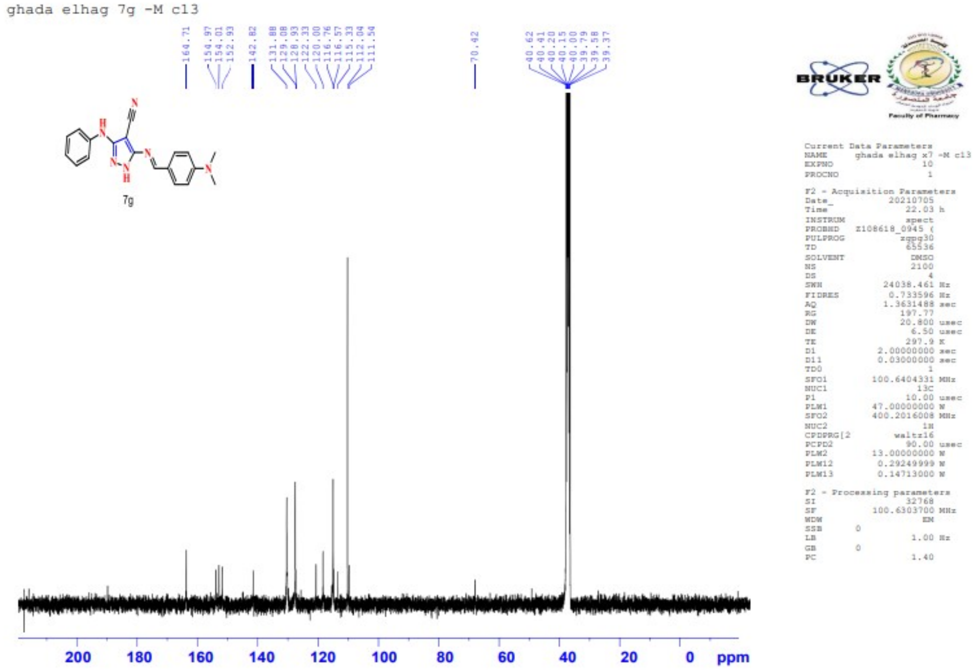

S32: ^{1}H-NMR of compound **7d**

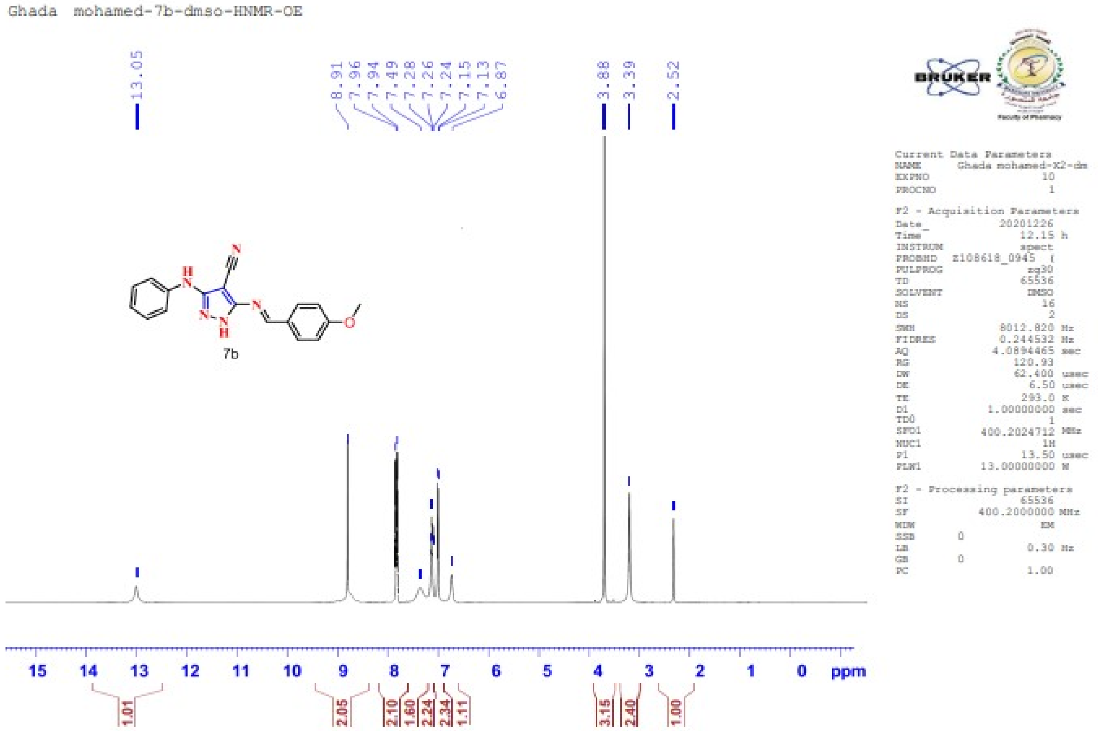

S33: ^{13}C-NMR of compound **7d**

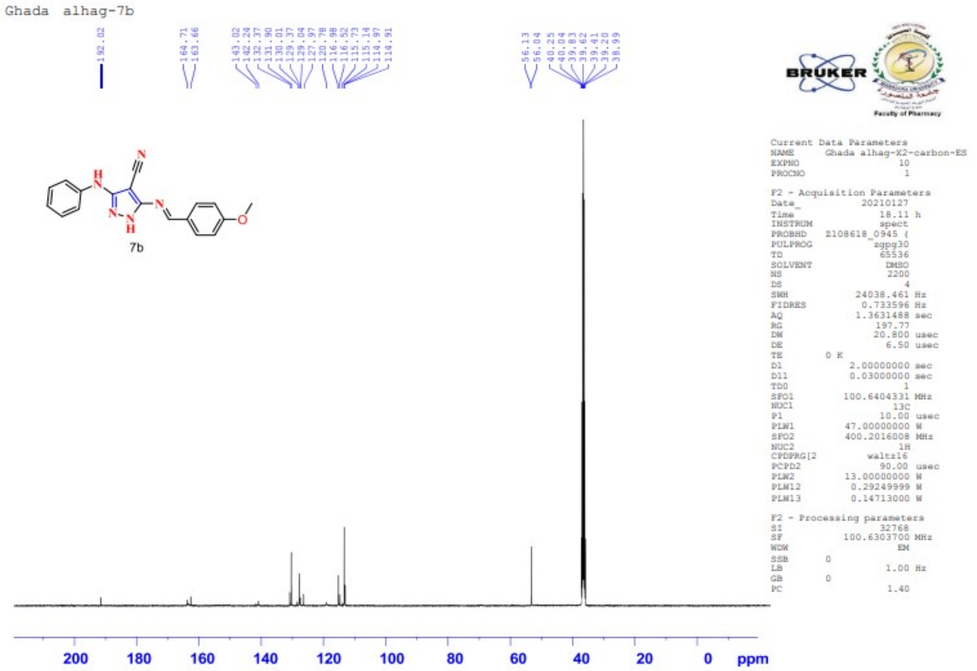

S34: ^{1}H-NMR of compound **8a**

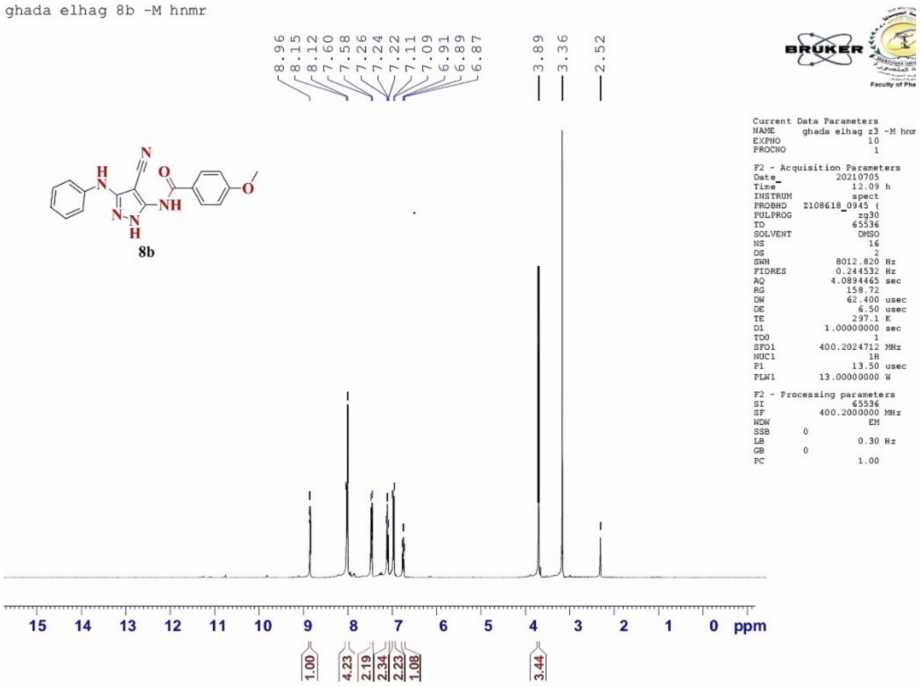

S35: ^{13}C-NMR of compound **8a**

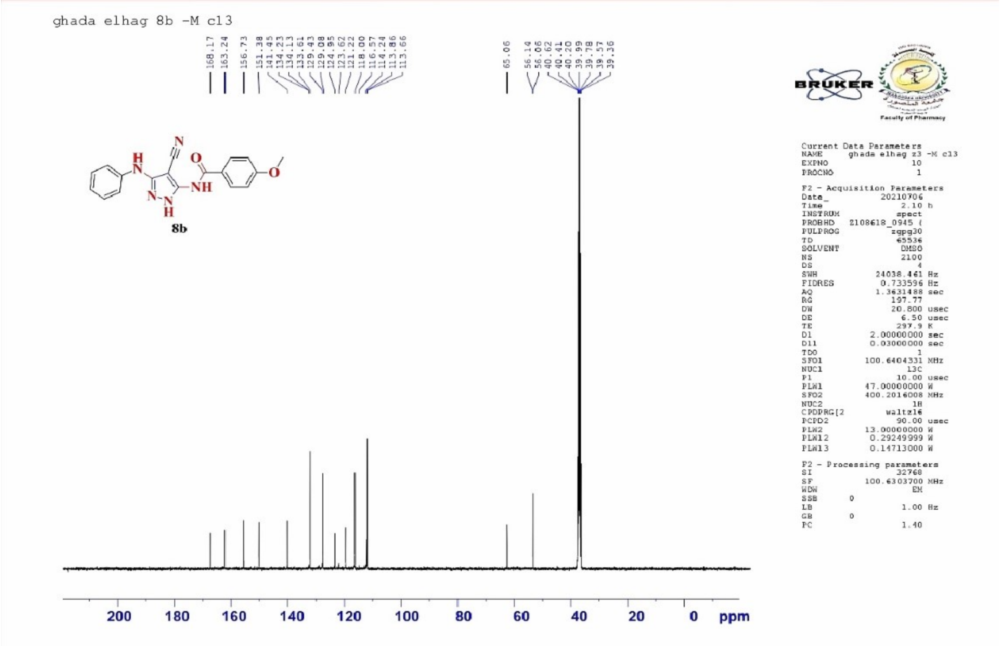

S36: ^{1}H-NMR of compound **8b**

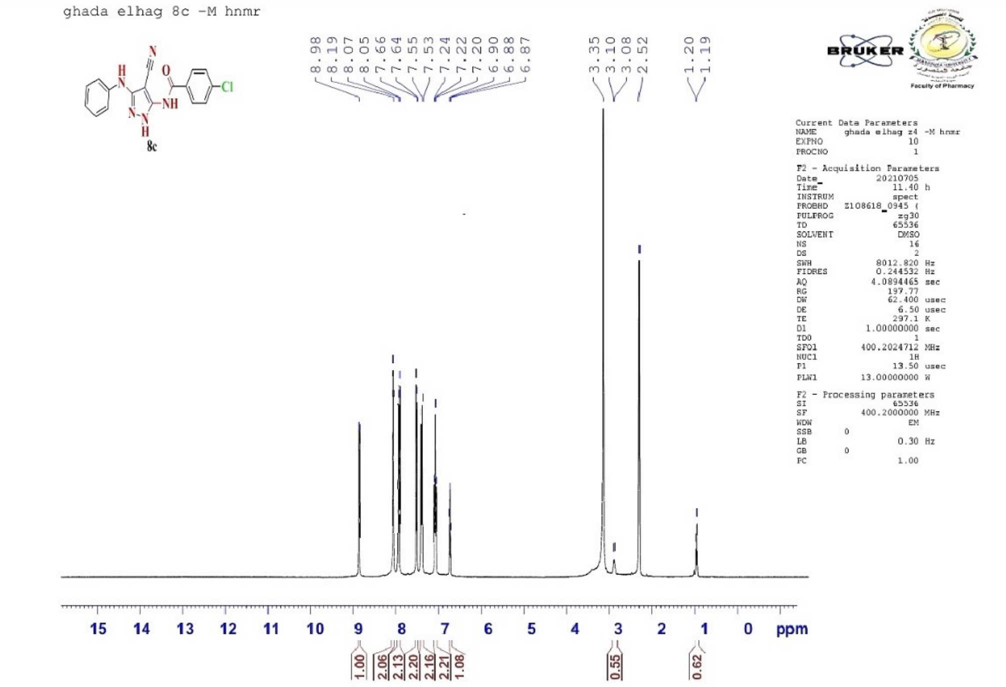

S37: ^{1}H-NMR of compound **9**

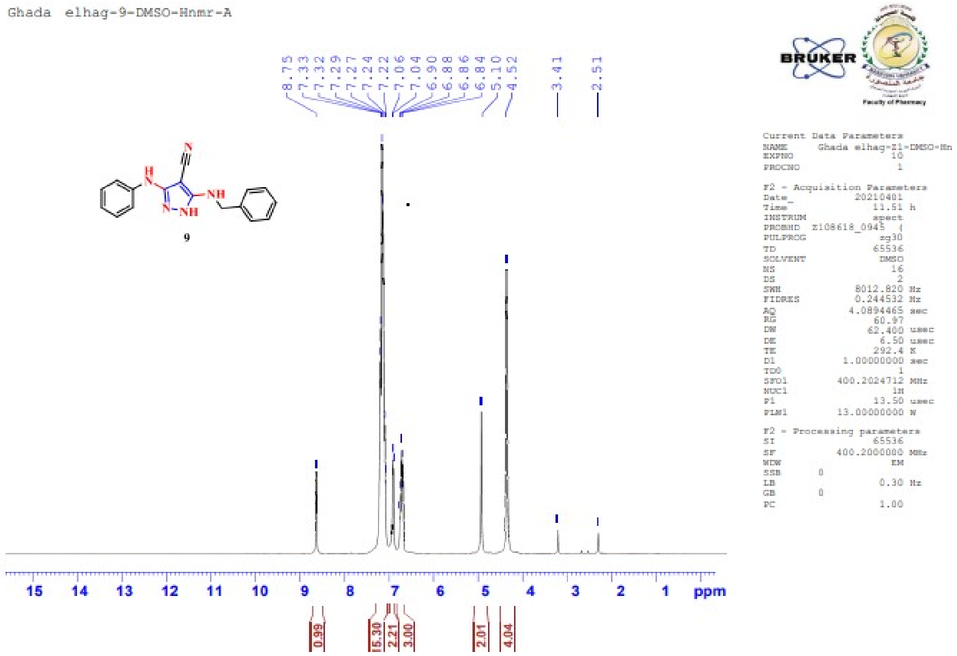

S38: ^{13}C-NMR of compound **9**

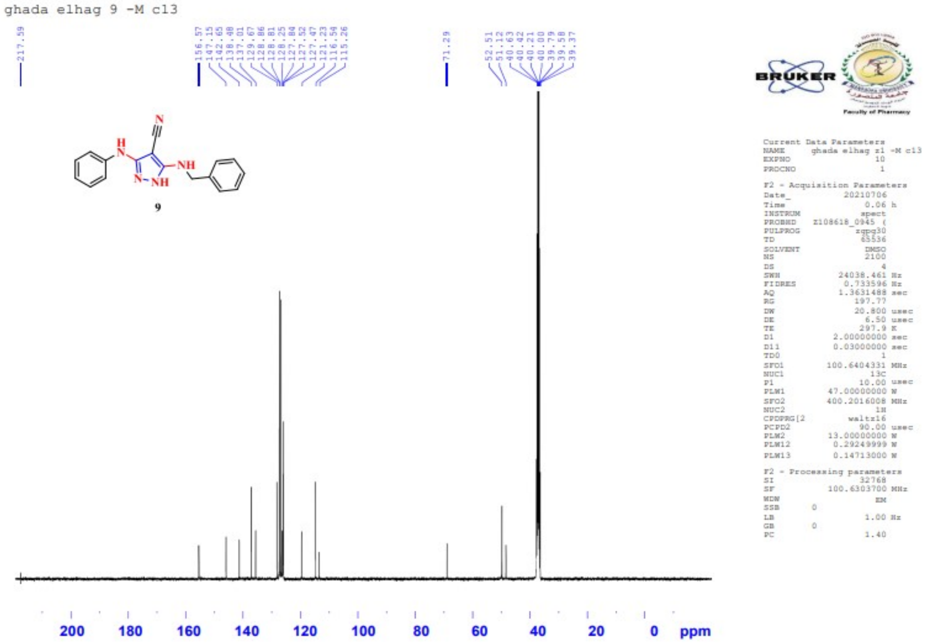

S39: ^{1}H-NMR of compound **10**

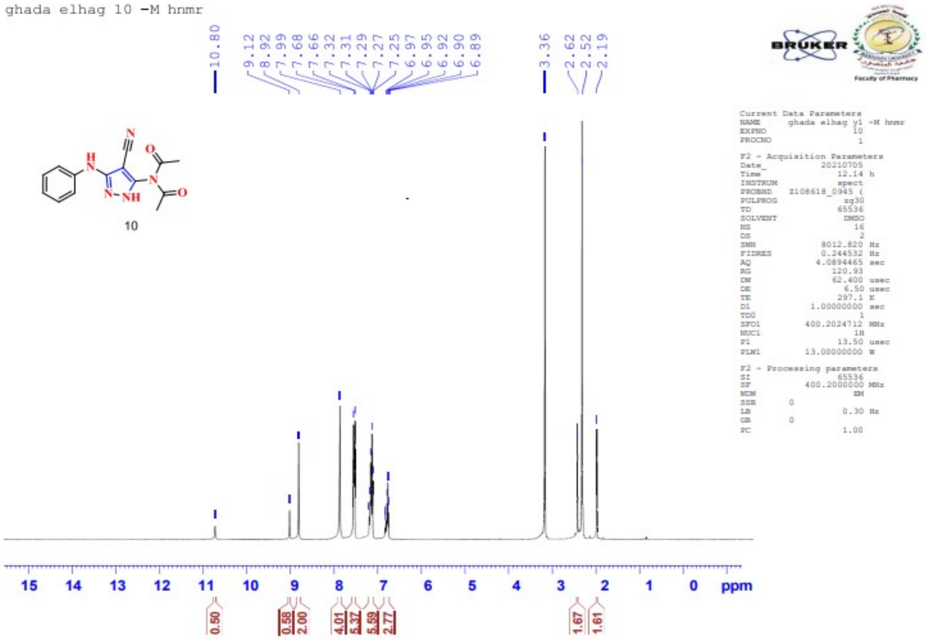

S40: ^{13}C-NMR of compound **10**

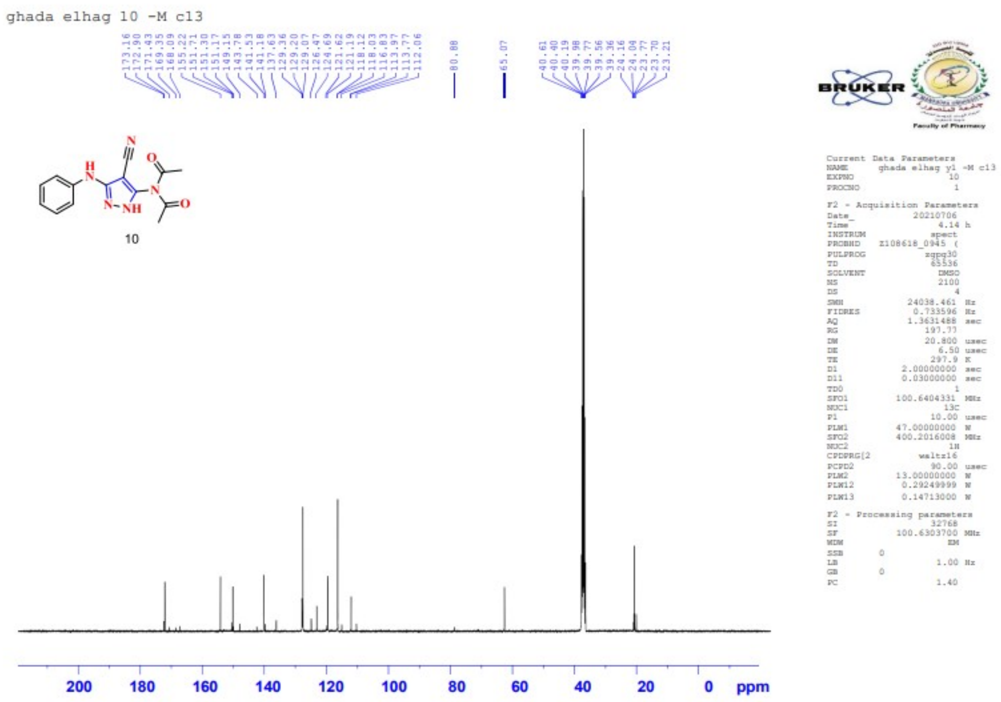

S41: IR of compound **2a**

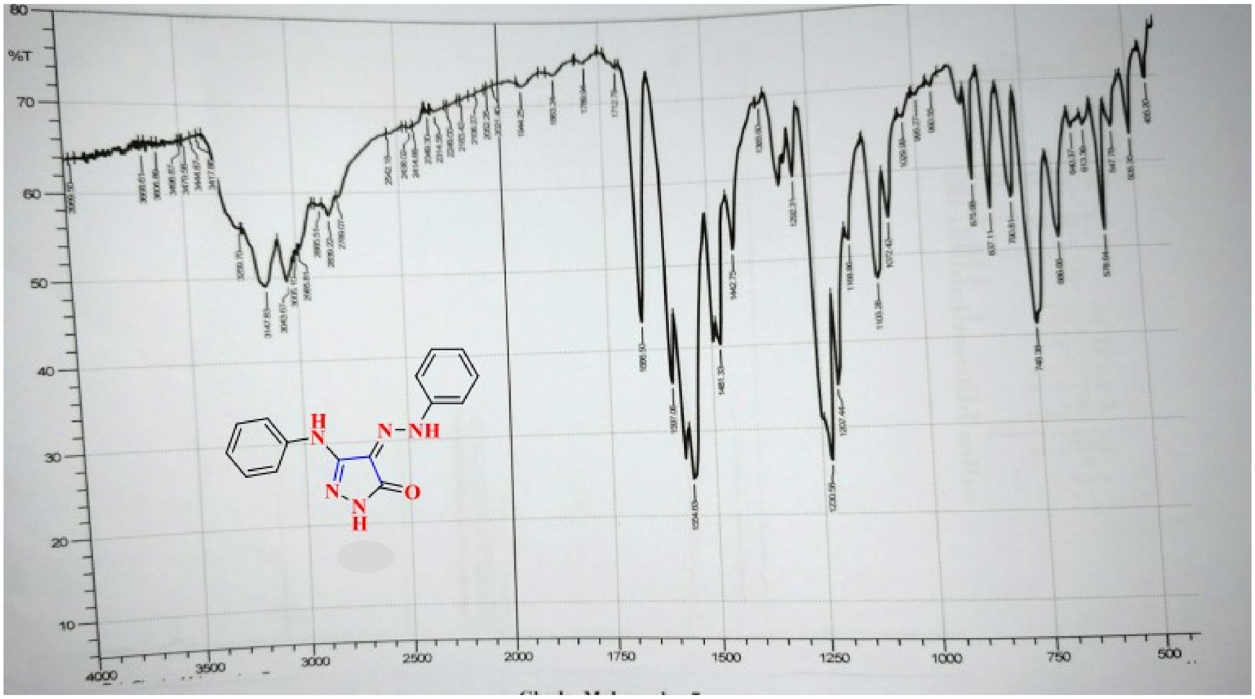

S42: IR of compound **2b**

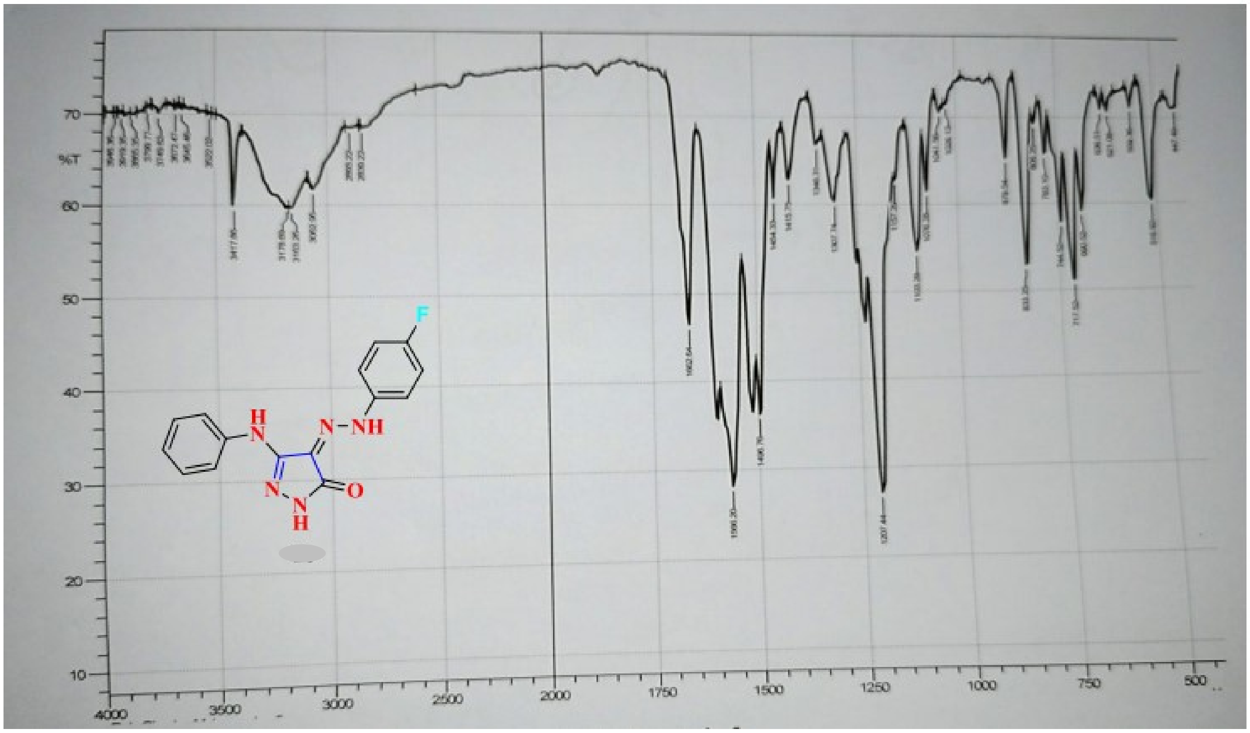

S43: IR of compound **2e**

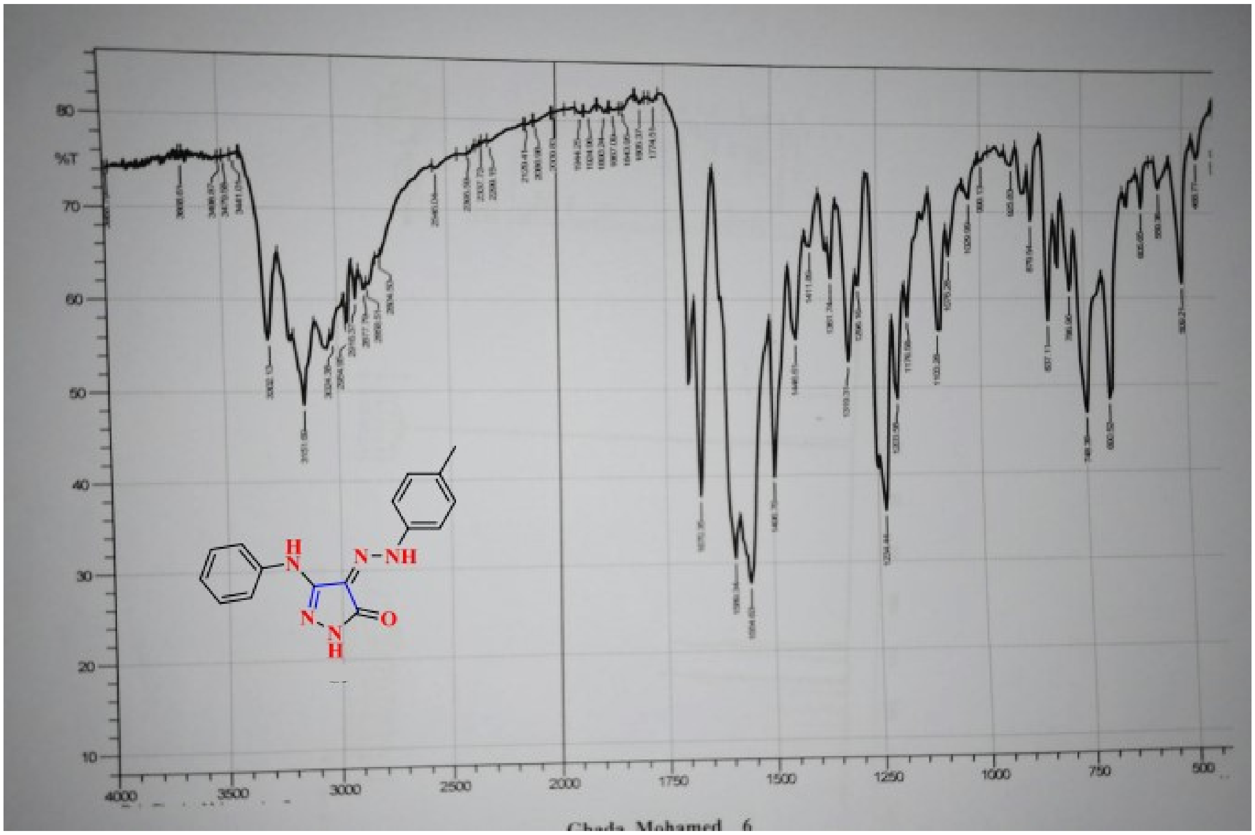

S44: IR of compound **2f**

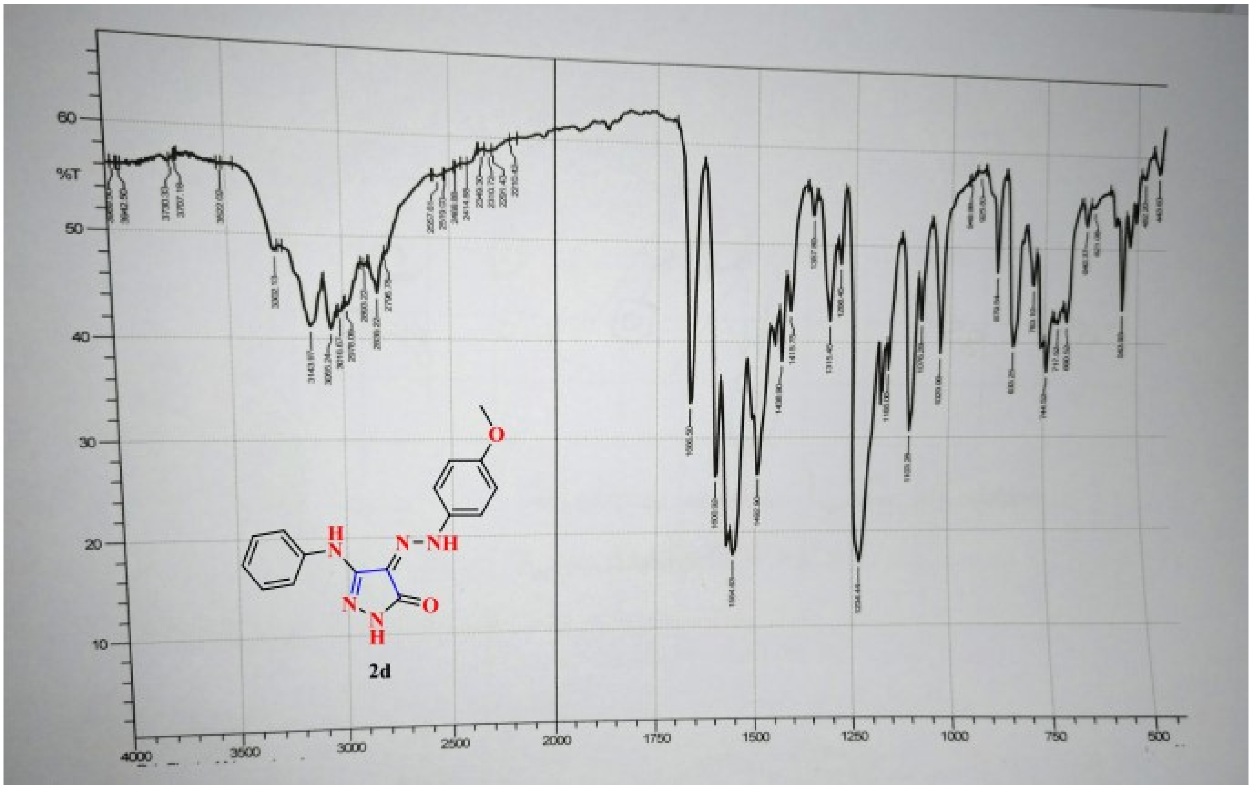

S45: IR of compound **2g**

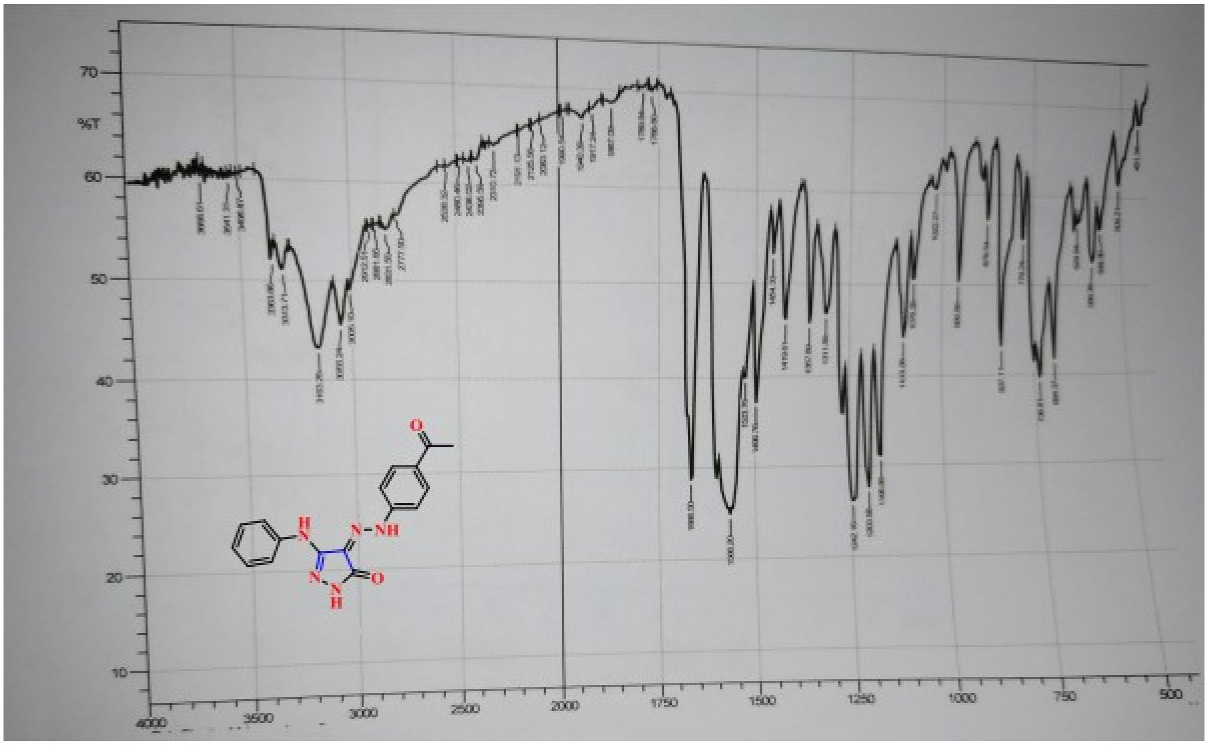

S46: Inhibition of CDK2/CyclinA2 by compounds **2a-g, 4, 7a-d, 8a-b, 9 &10** at 50 μM.

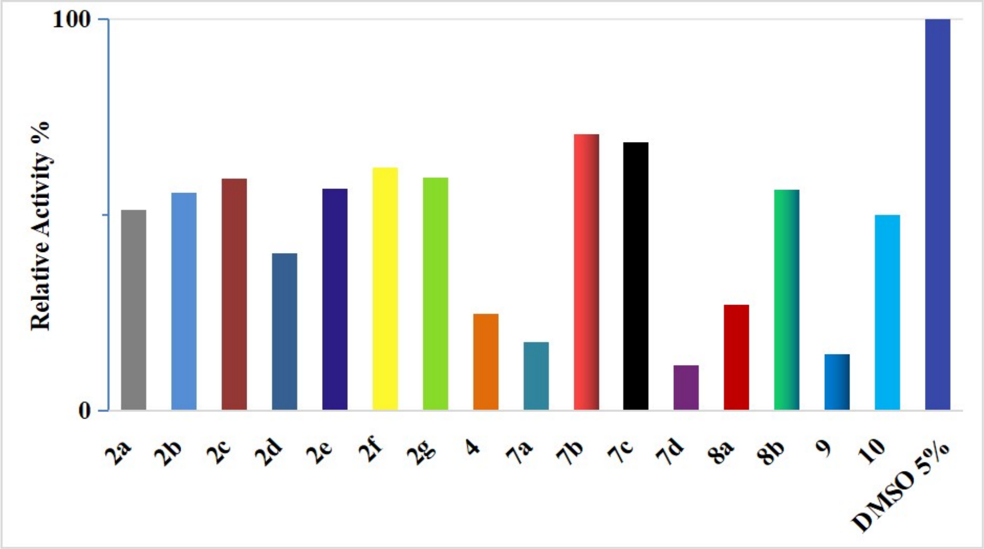

S47: The dose–response curves show the Inhibitory Concentration at 50% (IC_{50}) curves of compounds **4**, **7c**, **7d**, **8b and 9** in CDK2/Cyclin A2 protein kinase activity assay.

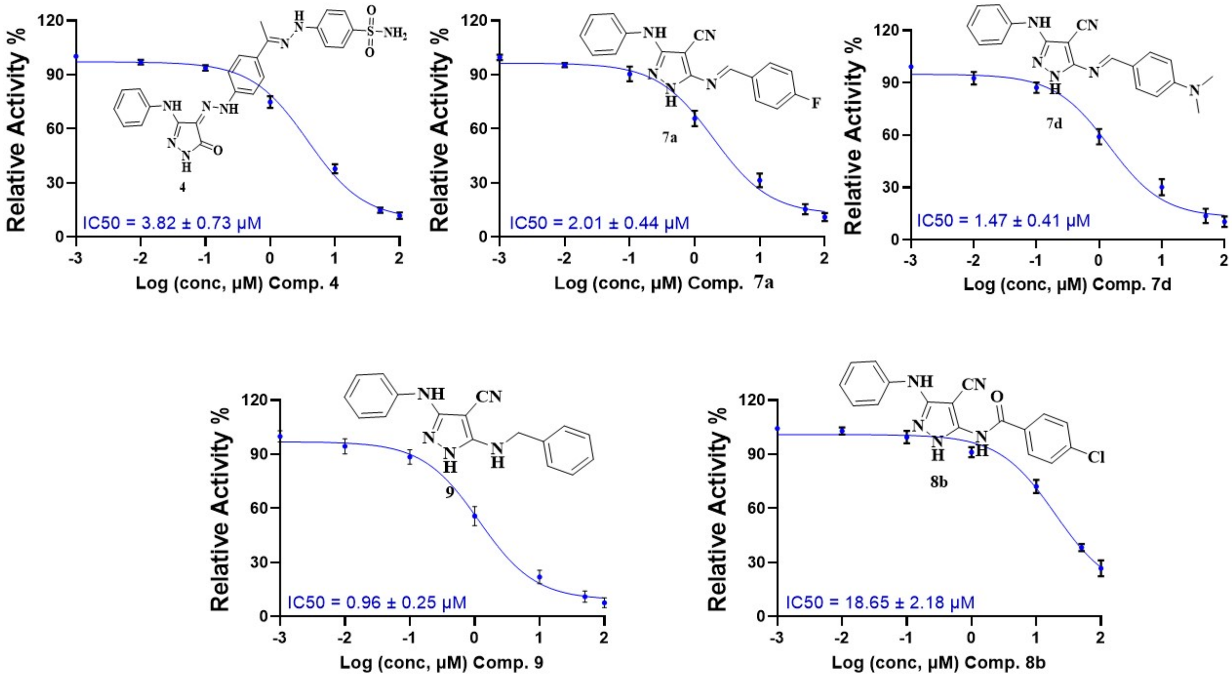

S48: Flow cytometric analysis for cell cycle distribution. (A) Control HCT-116, (B) Compound **4**, and (C) graphical representation for cell cycle distribution analysis among differently treated cells.

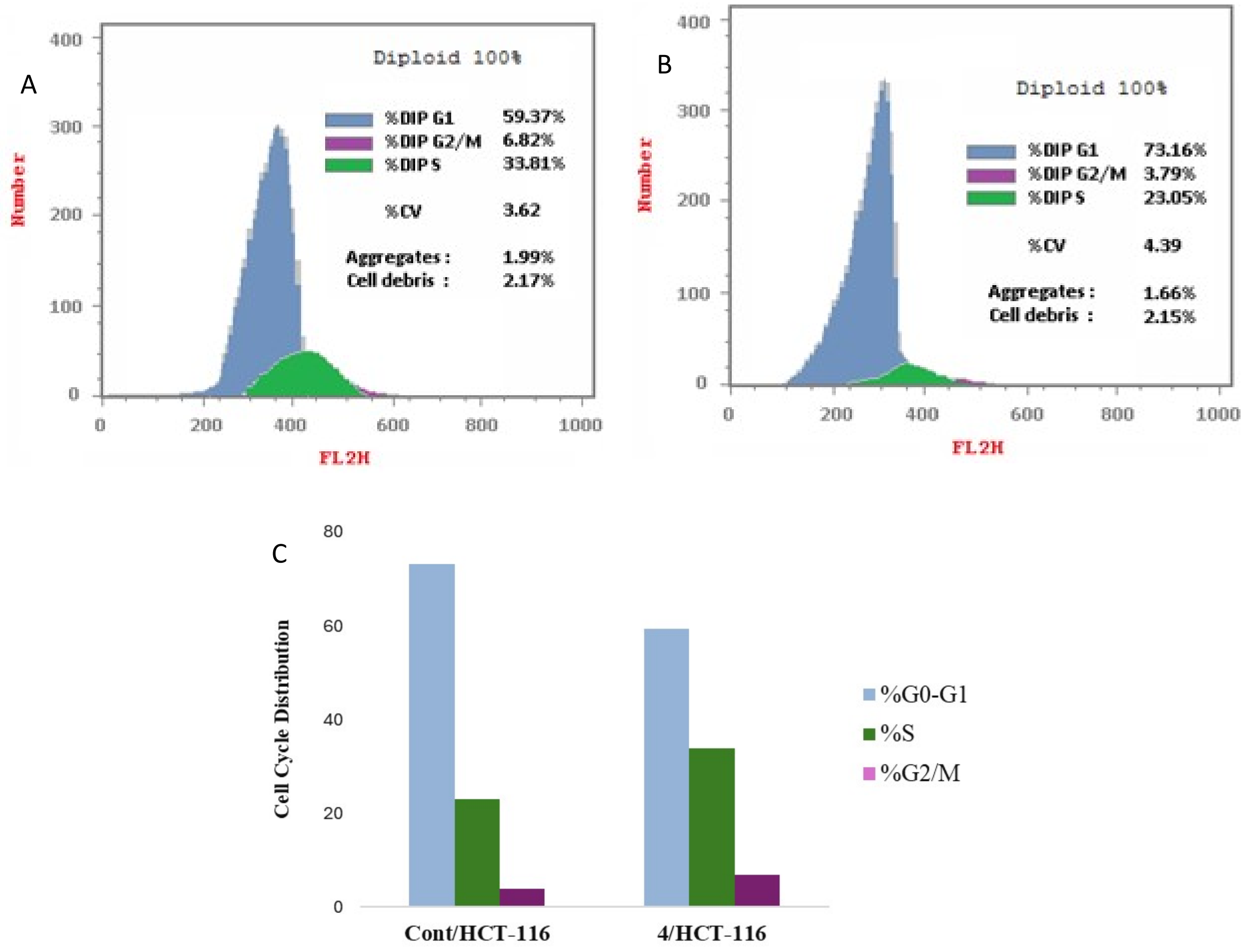

S49: Flow cytometric analysis of apoptosis among treated cells. (A) Control HCT-116, (B) Compound **4**, (C) Graphical illustration of apoptosis % among differently treated cells.

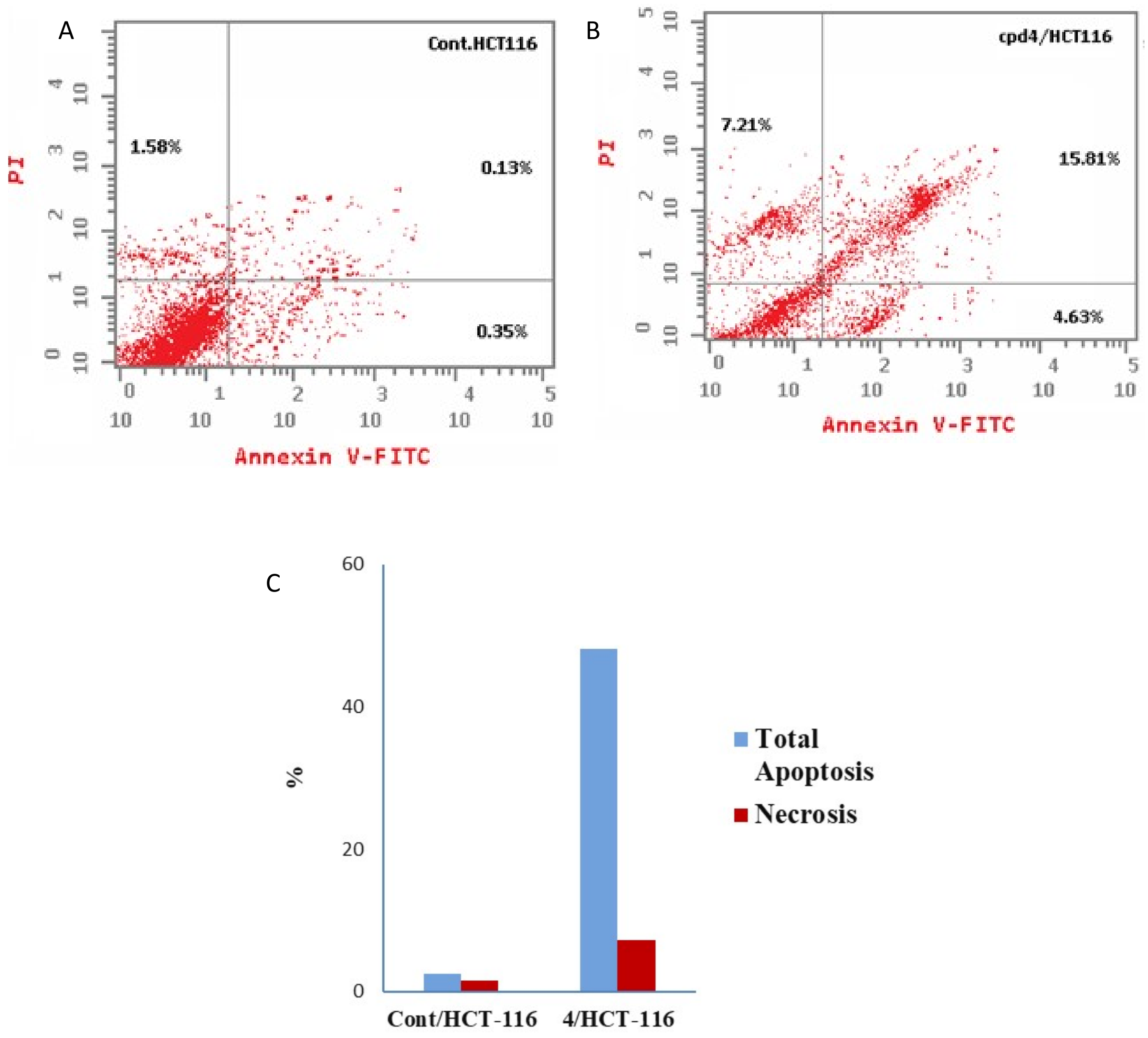

S50: Cell cycle and apoptosis raw data

Ser	Sample			Cytotoxicity IC50 uM	SD
	code	M.W g/mol	cells	HCT116	±
1	**cpd4**	490.54	---	**1.806**	0.07
2	**cpd9**	289.33	---	**10.003**	0.39
3	**Staurosporine**	466.54	---	**7.022**	0.27

ser	Sample		DNA content			
	code	IC50 uM	%G0-G1	%S	%G2/M	Comment
**1**	cpd4/**HCT116**	---	73.16	23.05	3.79	cell growth arrest@ G1
**2**	**Cont.HCT116**	---	59.37	33.81	6.82	---

s	code	conc	Apoptosis			Necrosis
			Total	Early	Late	
**1**	cpd4/**HCT116**	---	**27.65**	**4.63**	**15.81**	**7.21**
**2**	**Cont.HCT116**	---	**2.06**	**0.35**	**0.13**	**1.58**

S51: Western Blot assay for compounds **4**, **9**

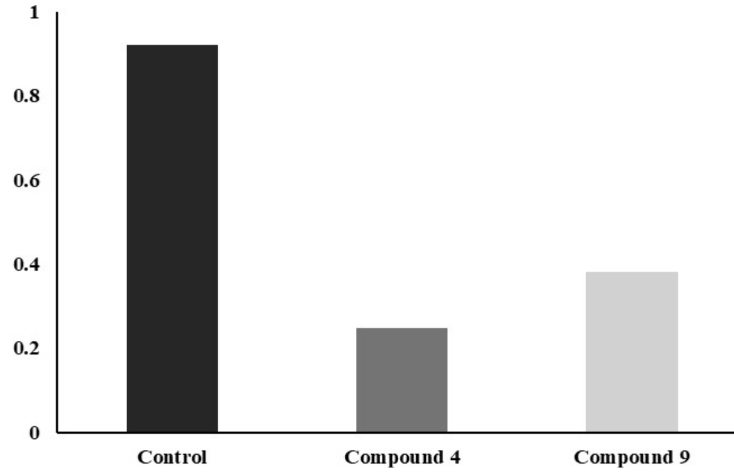

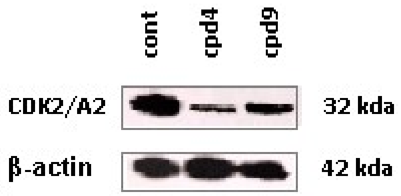

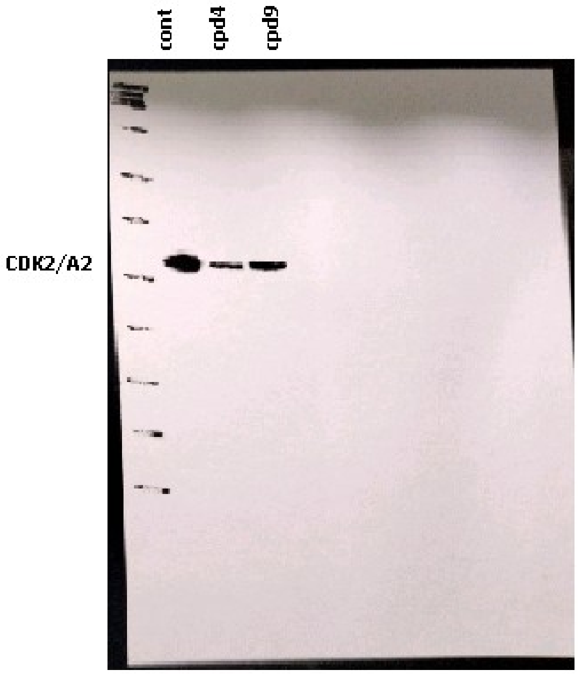

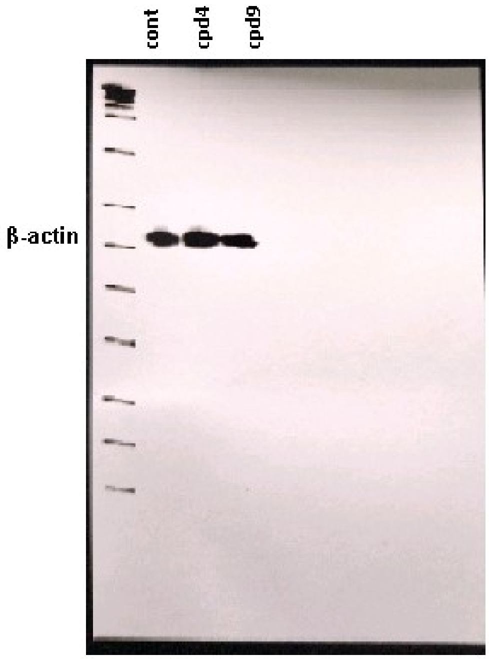

S52: Mass Results compound **4**

The Regional Center For mycology And Biotechnology
RCMB

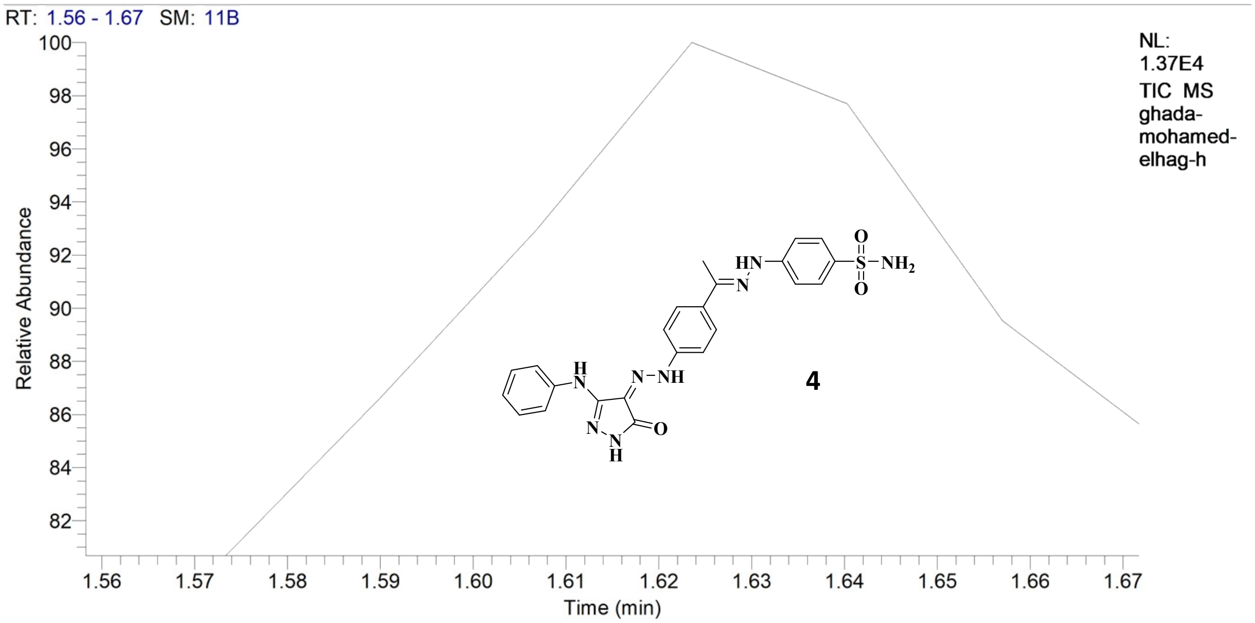

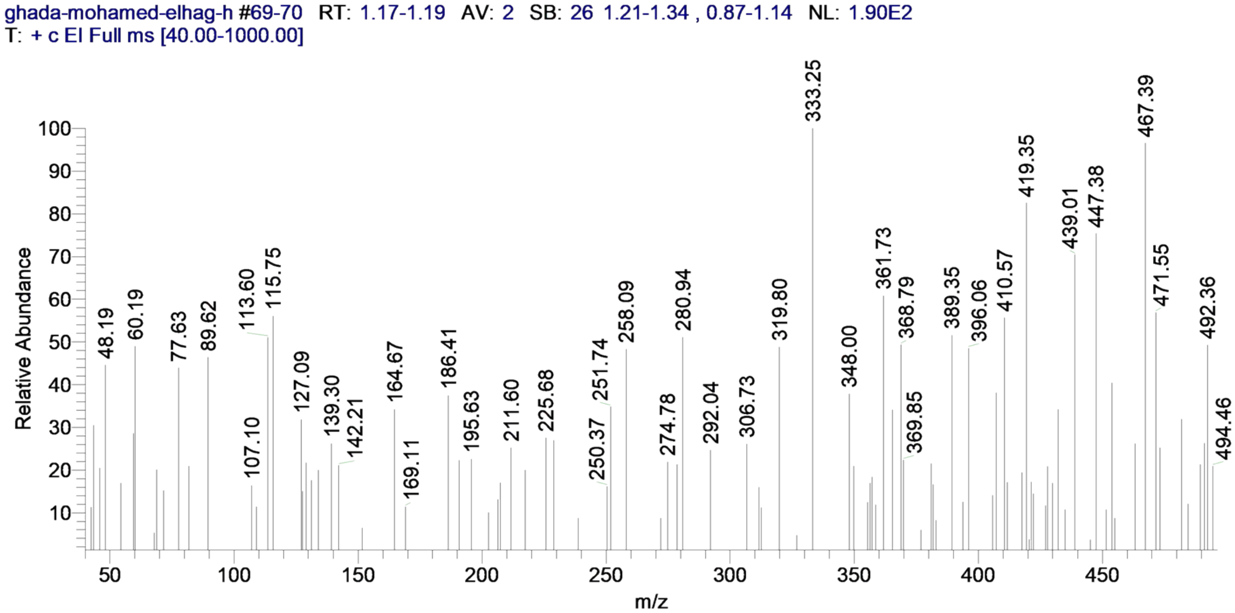

S53: Mass Results compound **7d**

The Regional Center For mycology And Biotechnology
RCMB

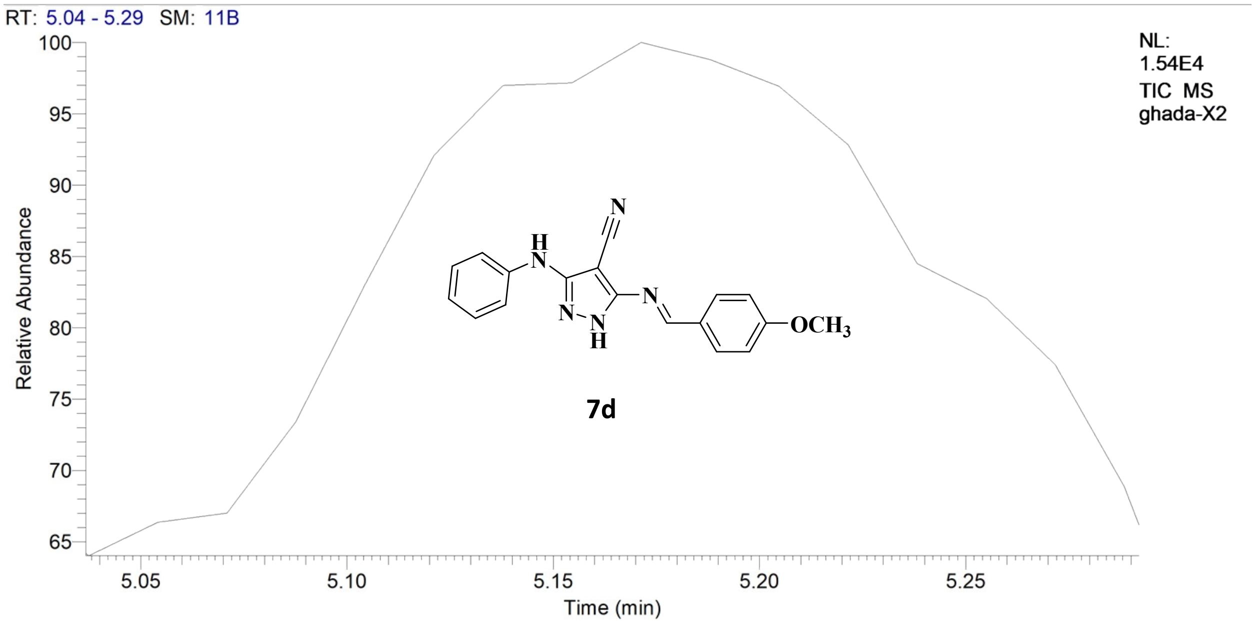

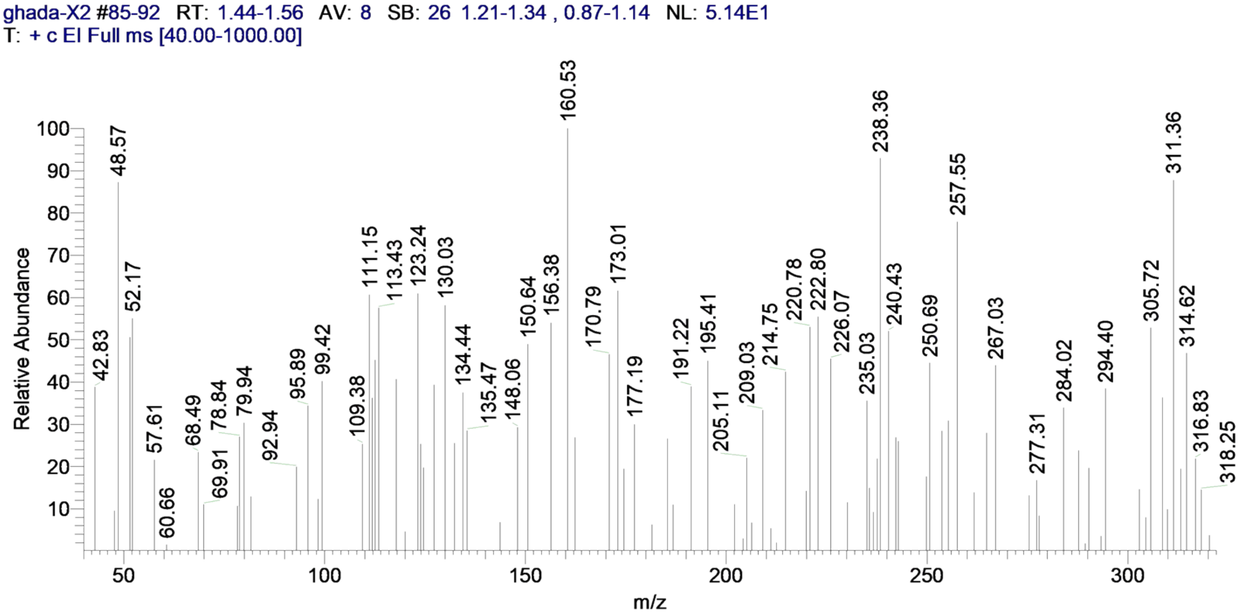

S54: Mass results compound **8a**

The Regional Center For mycology And Biotechnology
RCMB

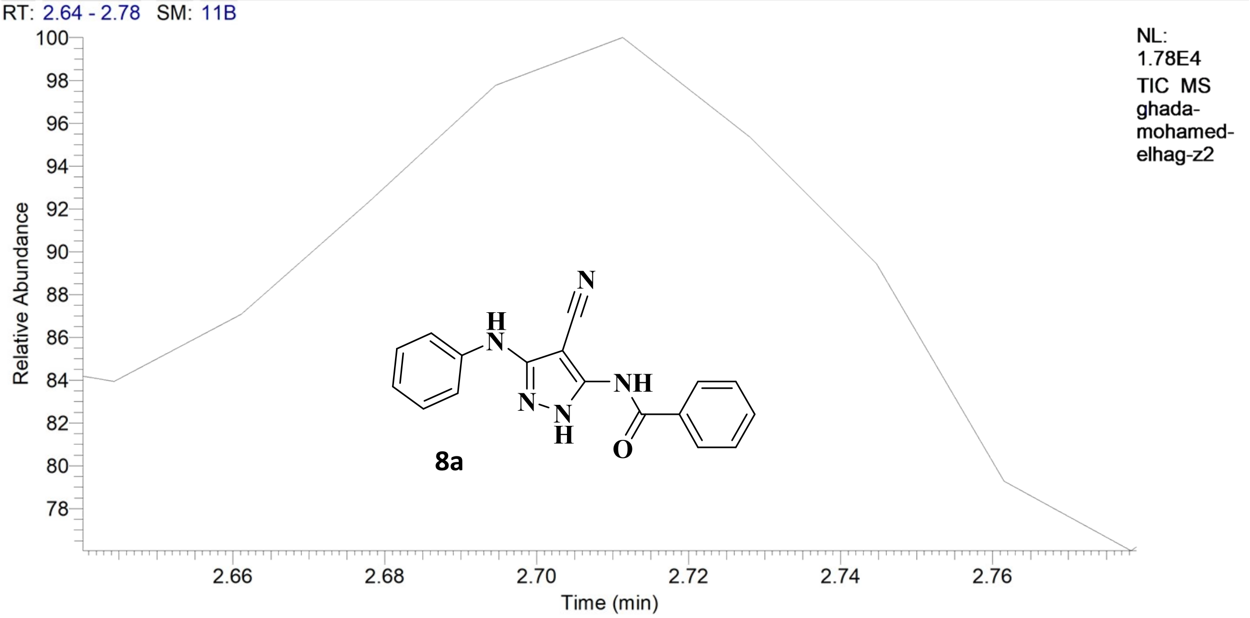

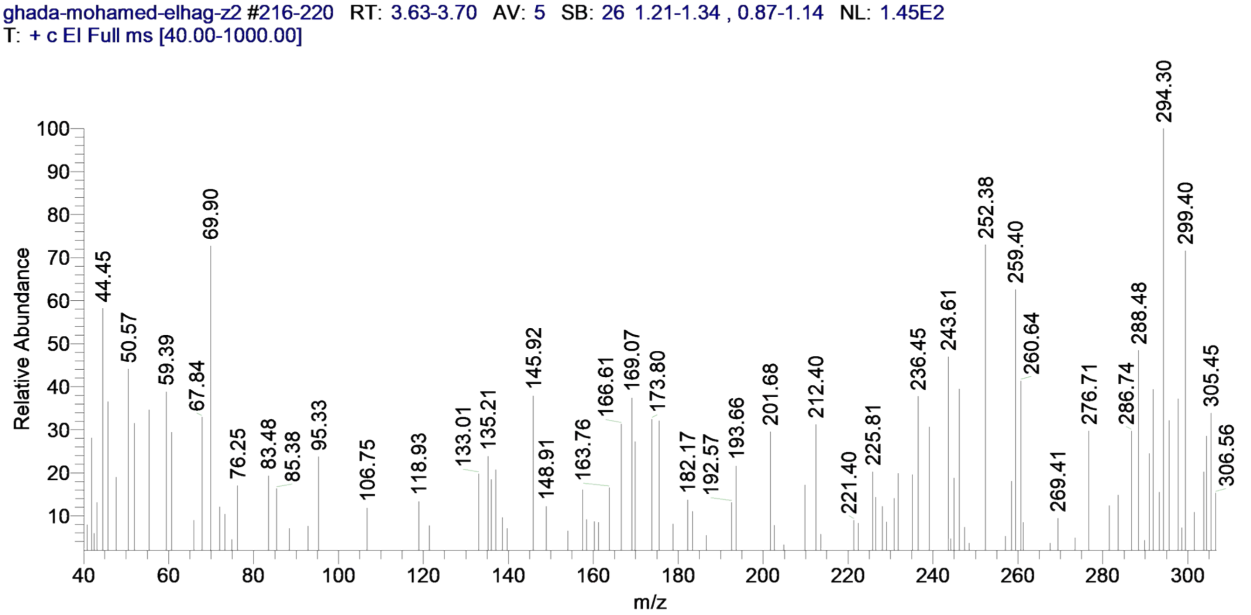

S55: Mass Results compound **9**

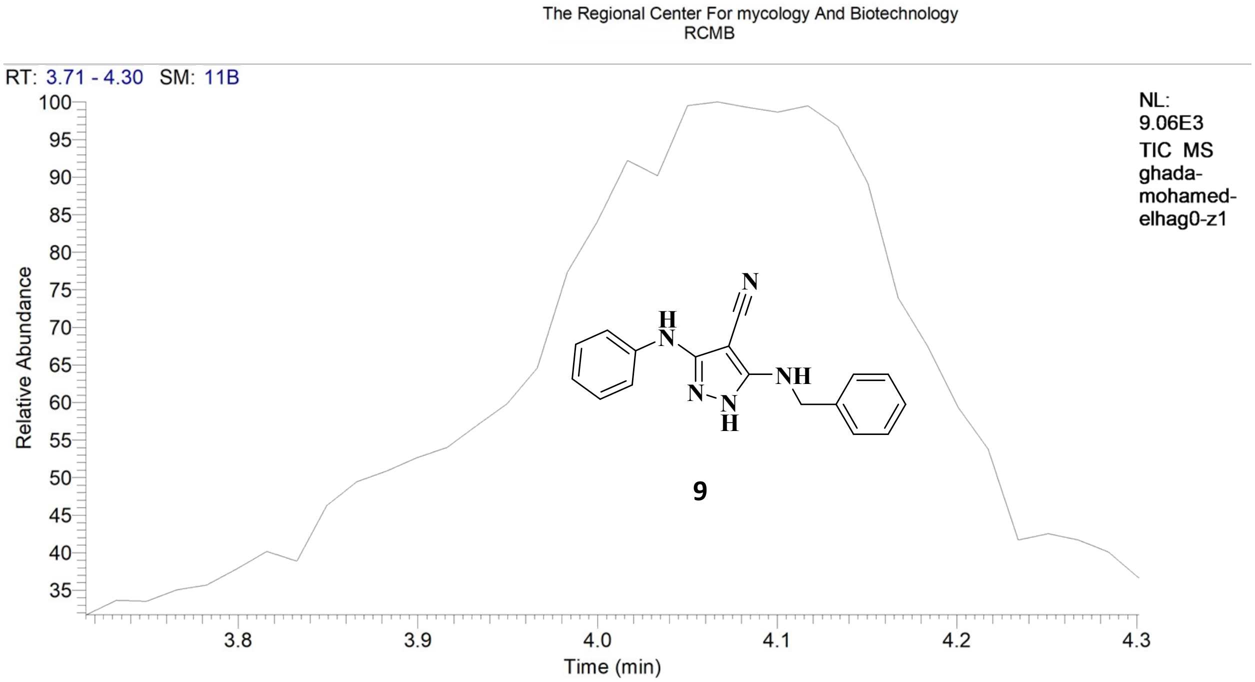

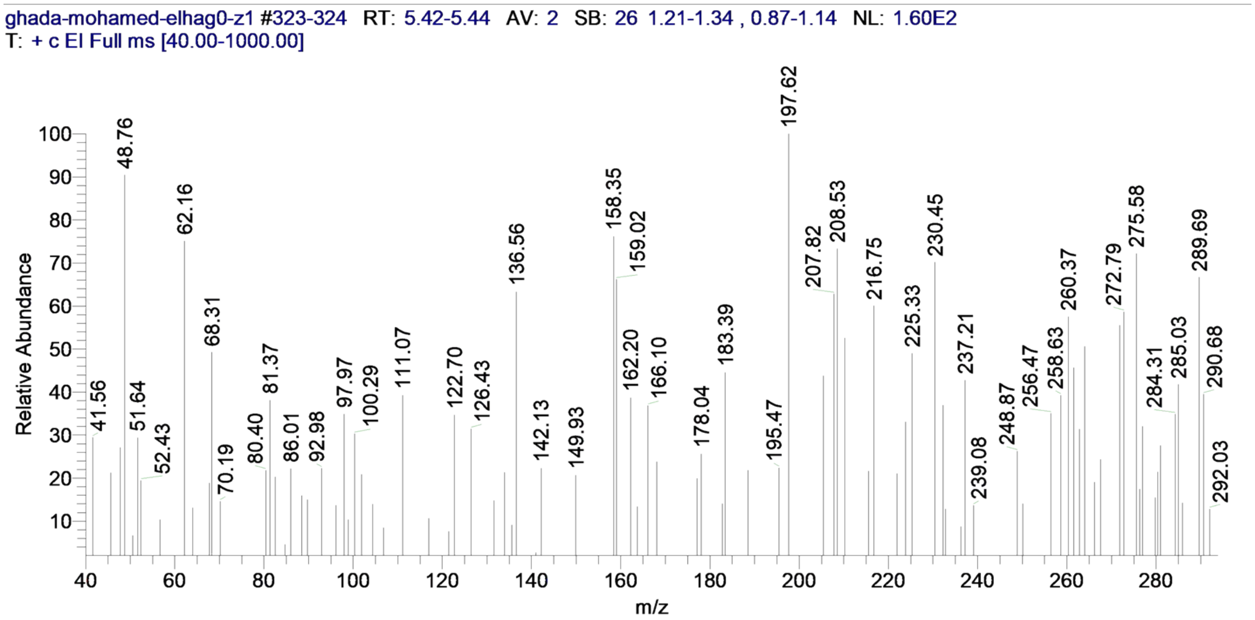

S56: Mass Results compound **10**

The Regional Center For mycology And Biotechnology
RCMB

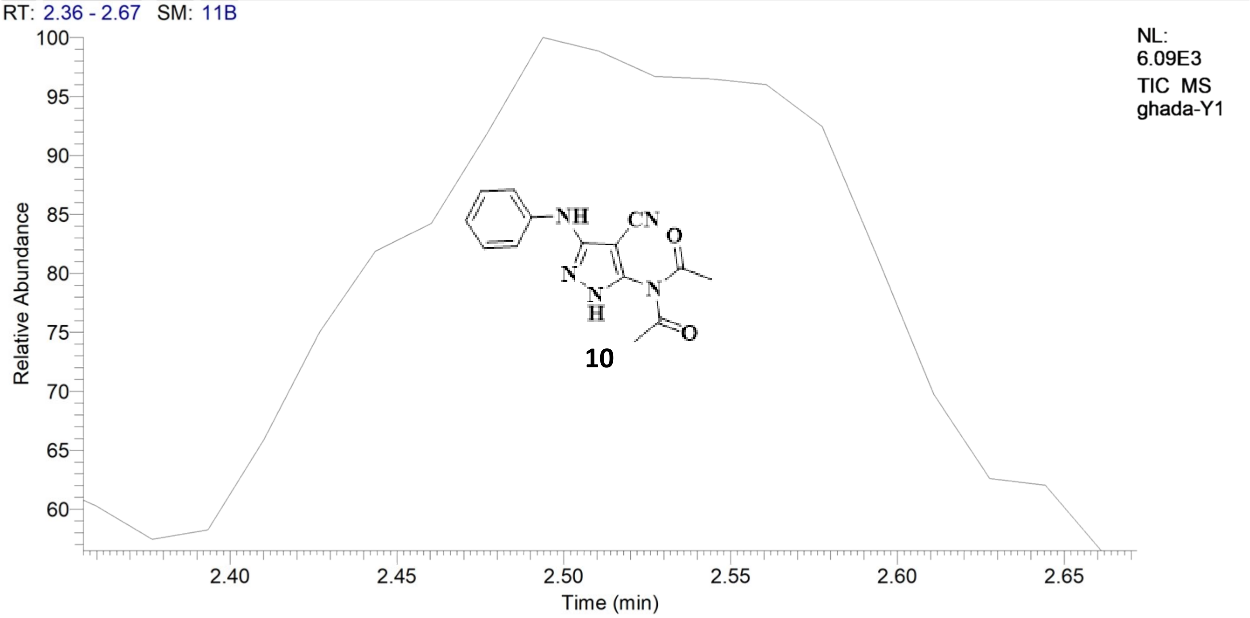

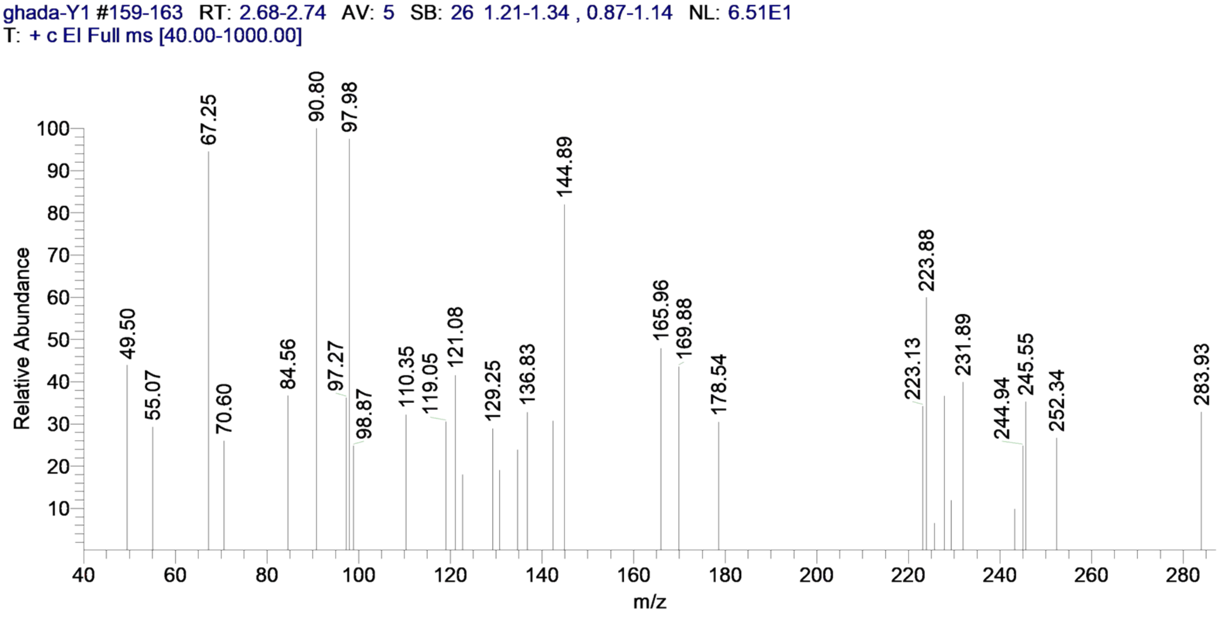

Supplement: RA-014-D4RA06500J-s001 [file RA-014-D4RA06500J-s001.pdf]
